# Estimating the health impact of vaccination against ten pathogens in 98 low-income and middle-income countries from 2000 to 2030: a modelling study

**DOI:** 10.1016/S0140-6736(20)32657-X

**Published:** 2021-01-30

**Authors:** Xiang Li, Christinah Mukandavire, Zulma M Cucunubá, Susy Echeverria Londono, Kaja Abbas, Hannah E Clapham, Mark Jit, Hope L Johnson, Timos Papadopoulos, Emilia Vynnycky, Marc Brisson, Emily D Carter, Andrew Clark, Margaret J de Villiers, Kirsten Eilertson, Matthew J Ferrari, Ivane Gamkrelidze, Katy A M Gaythorpe, Nicholas C Grassly, Timothy B Hallett, Wes Hinsley, Michael L Jackson, Kévin Jean, Andromachi Karachaliou, Petra Klepac, Justin Lessler, Xi Li, Sean M Moore, Shevanthi Nayagam, Duy Manh Nguyen, Homie Razavi, Devin Razavi-Shearer, Stephen Resch, Colin Sanderson, Steven Sweet, Stephen Sy, Yvonne Tam, Hira Tanvir, Quan Minh Tran, Caroline L Trotter, Shaun Truelove, Kevin van Zandvoort, Stéphane Verguet, Neff Walker, Amy Winter, Kim Woodruff, Neil M Ferguson, Tini Garske

**Affiliations:** a MRC Centre for Global Infectious Disease Analysis, Abdul Latif Jameel Institute for Disease and Emergency Analytics (J-IDEA), School of Public Health, Imperial College London, London, UK; b Section of Hepatology and Gastroenterology, Department of Metabolism, Digestion and Reproduction, Imperial College London, London, UK; c London School of Hygiene & Tropical Medicine; d Saw Swee Hock School of Public Health, National University of Singapore, Singapore; e Oxford University Clinical Research Unit, Ho Chi Minh City, Vietnam; f Nuffield Department of Medicine, Oxford University, Oxford, UK; g University of Hong Kong, Hong Kong Special Administrative Region, China; h Public Health England, London, UK; i Gavi, the Vaccine Alliance, Geneva, Switzerland; j University of Southampton, Southampton, UK; k Laval University, Quebec, QC, Canada; l Department of International Health, Bloomberg School of Public Health, Johns Hopkins University, Baltimore, MD, USA; m Department of Epidemiology, Bloomberg School of Public Health, Johns Hopkins University, Baltimore, MD, USA; n Colorado State University, Fort Collins, CO, USA; o Pennsylvania State University, Pennsylvania, PA, USA; p Center for Disease Analysis Foundation, Lafayette, CO, USA; q Kaiser Permanente Washington, Seattle, WA, USA; r Laboratoire MESuRS, Conservatoire National des Arts et Métiers, Paris, France; s Unité PACRI, Institut Pasteur, Conservatoire National des Arts et Métiers, Paris, France; t University of Cambridge, Cambridge, UK; u Atlanta, GA, USA; v Department of Biological Sciences, University of Notre Dame, Notre Dame, IN, USA; w School of Computing, Dublin City University, Dublin, Ireland; x Center for Health Decision Science, Harvard T H Chan School of Public Health, Harvard University, Cambridge, MA, USA; y Department of Global Health and Population, Harvard T H Chan School of Public Health, Harvard University, Cambridge, MA, USA

## Abstract

**Background:**

The past two decades have seen expansion of childhood vaccination programmes in low-income and middle-income countries (LMICs). We quantify the health impact of these programmes by estimating the deaths and disability-adjusted life-years (DALYs) averted by vaccination against ten pathogens in 98 LMICs between 2000 and 2030.

**Methods:**

16 independent research groups provided model-based disease burden estimates under a range of vaccination coverage scenarios for ten pathogens: hepatitis B virus, *Haemophilus influenzae* type B, human papillomavirus, Japanese encephalitis, measles, *Neisseria meningitidis* serogroup A, *Streptococcus pneumoniae*, rotavirus, rubella, and yellow fever. Using standardised demographic data and vaccine coverage, the impact of vaccination programmes was determined by comparing model estimates from a no-vaccination counterfactual scenario with those from a reported and projected vaccination scenario. We present deaths and DALYs averted between 2000 and 2030 by calendar year and by annual birth cohort.

**Findings:**

We estimate that vaccination of the ten selected pathogens will have averted 69 million (95% credible interval 52–88) deaths between 2000 and 2030, of which 37 million (30–48) were averted between 2000 and 2019. From 2000 to 2019, this represents a 45% (36–58) reduction in deaths compared with the counterfactual scenario of no vaccination. Most of this impact is concentrated in a reduction in mortality among children younger than 5 years (57% reduction [52–66]), most notably from measles. Over the lifetime of birth cohorts born between 2000 and 2030, we predict that 120 million (93–150) deaths will be averted by vaccination, of which 58 million (39–76) are due to measles vaccination and 38 million (25–52) are due to hepatitis B vaccination. We estimate that increases in vaccine coverage and introductions of additional vaccines will result in a 72% (59–81) reduction in lifetime mortality in the 2019 birth cohort.

**Interpretation:**

Increases in vaccine coverage and the introduction of new vaccines into LMICs have had a major impact in reducing mortality. These public health gains are predicted to increase in coming decades if progress in increasing coverage is sustained.

**Funding:**

Gavi, the Vaccine Alliance and the Bill & Melinda Gates Foundation.

## Introduction

Vaccines have been responsible for substantial reductions in mortality^1^, ^2^, ^3^, ^4^, ^5^ and are among the most cost-effective health interventions.^6^, ^7^, ^8^ In addition to direct protection provided to vaccinated individuals, high levels of vaccination coverage offer indirect protection (herd immunity) to the remaining unvaccinated individuals in a population. The timescale of vaccine impact varies considerably; for some childhood diseases (such as measles, rotavirus, and pneumococcal disease), impact is seen rapidly, whereas for human papillomavirus (HPV) and hepatitis B, vaccine impact is commonly seen over a much longer timescale in the reduction of adult morbidity and mortality.

WHO introduced the Expanded Programme on Immunization in 1974.^9^ This programme, which was supported by UNICEF and global donors, succeeded in delivering substantial increases in coverage of routine childhood vaccines; for example, global coverage of three doses of diphtheria-tetanus-pertussis vaccine (DTP3) increased from just over 20% in 1980 to more than 75% in 1990.^10^ However, as coverage plateaued in the 1990s, concerns grew around the sustainability of these gains, eventually leading to the formation of Gavi, the Vaccine Alliance, in 1999.^11^ Gavi's mission is to sustain and increase coverage and improve access to new vaccines in low-income and middle-income countries (LMICs).^12^ Since its founding, it has supported the immunisation of more than 700 million children in LMICs.^13^ Global targets for vaccination have also continued to grow in ambition; the Global Vaccine Action Plan framework was launched in 2012 by WHO with the aim of preventing millions of deaths by 2020 through access to vaccines in all countries. This was further reinforced by target 3.8 of the Sustainable Development Goals calling for access to vaccines for all by 2030.^14^

**Research in context**

**Evidence before this study**

Immunisation has been a key tool for reducing childhood mortality in low-income and middle-income countries (LMICs) in the past few decades. However, quantitative assessment of the impact of recent expansion of vaccination programmes on mortality and morbidity has been scarce. We searched PubMed up to Sept 25, 2019, without date limits or language restrictions, using the search terms “vaccine* AND impact AND estimate* AND (mortality or morbidity)”. We found 15 studies that estimated population-level mortality impact of past vaccination against at least one of the pathogens considered in our study, in at least one LMIC. Of these, three studies considered impact in more than one country, one of which assessed the impact of multiple vaccines in multiple countries. The latter study was published by the predecessor of the Vaccine Impact Modelling Consortium and reported the previous estimates of the impacts of vaccination on mortality in the 73 countries supported by Gavi.

**Added value of this study**

The current study advances previous work in terms of scale (number of countries, number of pathogens, and time period), in its emphasis on standardising model inputs (vaccine coverage and demography) and outputs (mortality and disability-adjusted life-years averted), and in assessing uncertainty in estimates of vaccine impact. Standardisation allowed impacts to be combined across and compared between vaccines. Uncertainty was assessed via probabilistic sensitivity analysis and by combining outputs from multiple models for each disease.

**Implications of all the available evidence**

Rigorous estimates of the impact of childhood vaccination programmes on morbidity and mortality inform public health investment decisions made by countries and global donors. The results highlight the importance of maintaining and increasing vaccine coverage to sustain gains made in reducing infectious disease-related mortality in LMICs.

Due to incompleteness and the inconsistent quality of death registration and disease surveillance systems in many LMICs, directly measuring the impact of vaccination programmes on mortality and morbidity is not always possible. Therefore, mathematical models are a valuable tool for extrapolating from available disease burden and pathogen surveillance data to generate impact estimates and to generate projections of the impact of future vaccine coverage to inform investment planning.

To improve the quality and coordination of vaccine impact assessment, the Vaccine Impact Modelling Consortium (VIMC) was formed in late 2016, with the support of Gavi and the Bill & Melinda Gates Foundation. VIMC currently comprises 18 modelling groups coordinated by a secretariat at Imperial College London (London, UK), and it models vaccination impact against diseases caused by ten different pathogens across 98 countries (about 69% of the world's population in 2018), including the 73 countries currently eligible for Gavi support.

This study presents the first complete set of vaccine impact estimates generated since VIMC was formed, quantifying impact over calendar year and annual birth cohorts between 2000 and 2030, in terms of deaths and disability-adjusted life-years (DALYs) averted. The pathogens included were hepatitis B virus, *Haemophilus influenzae* type B, HPV, Japanese encephalitis, measles, *Neisseria meningitidis* serogroup A, *Streptococcus pneumoniae* (prevented by the pneumococcal conjugate vaccine [PCV]), rotavirus, rubella virus, and yellow fever virus. VIMC does not currently assess the impacts of DTP vaccine,^1^ cholera vaccine, or polio vaccines. The vaccine impact estimates from VIMC are used to support the monitoring of existing vaccination programmes and inform future investment strategy.

## Methods

### Models

Modelling groups from different institutions with disease-specific expertise provided pathogen-specific vaccine impact estimates for hepatitis B virus, *H influenzae* type B, HPV, Japanese encephalitis, measles, *N meningitidis* serogroup A, *S pneumoniae* (prevented by PCV), rotavirus, rubella virus, and yellow fever virus (table 1). Two mathematical models were used for each pathogen other than hepatitis B virus, which had three, and yellow fever, which had one. Including multiple models for each pathogen facilitates assessment of structural uncertainty in models. Each model represents the impact of vaccine coverage and efficacy on national-level disease burden (and in some cases disease transmission dynamics) to estimate vaccine impact. Model descriptions and the list of 98 LMICs included in the analysis are provided in appendix 2 (pp 14–35, 45–48). Static models estimate only the direct effect of vaccination on vaccinated cohorts, assuming that pathogen transmission intensity is not modified by vaccination coverage. Dynamic models simulate infectious disease transmission dynamics and model both the direct effect of vaccination on vaccinated cohorts and the indirect effect of vaccination on unvaccinated populations.

**Table 1:** Vaccination schedules and model information for the ten considered pathogens

	**Vaccination schedule**	**Lead institution for model (model type)**
Hepatitis B virus	Birth dose and three infant doses (<1 year)	Center for Disease Analysis (dynamic); Imperial College London (dynamic); independent model developed by Goldstein and colleagues^15^ (static)
Human papillomavirus	Two doses for adolescent girls (9–14 years)	Harvard School of Public Health (static); LSHTM (static)
*Haemophilus influenzae* type B	Three infant doses (<1 year)	Johns Hopkins University (static); LSHTM (static)
Japanese encephalitis	Infant dose (<1 year)	Oxford University (dynamic); University of Notre Dame (dynamic)
Measles	First dose at ≤1 year, second dose at <2 years	LSHTM (dynamic); Pennsylvania State University (dynamic)
*Neisseria meningitidis* serogroup A	Infant dose (<1 year)	University of Cambridge (dynamic); Kaiser Permanente Washington (dynamic)
*Streptococcus pneumoniae* (PCV)	Three infant doses (<1 year)	Johns Hopkins University (static); LSHTM (static)
Rotavirus	Two infant doses (<1 year)	Johns Hopkins University (static); LSHTM (static)
Rubella	First dose at <1 year, second dose at <2 years	Johns Hopkins University (dynamic); Public Health England (dynamic)
Yellow fever	Infant dose (<1 year)	Imperial College London (static)

Detailed descriptions of the models are provided in appendix 2 (pp 14–35). LSHTM=London School of Hygiene & Tropical Medicine. PCV=pneumococcal conjugate vaccine.

Standardised demographic data (live births per year and death rates) based on the UN World Population Prospects (UNWPP) 2017 was used for all countries.^16^ Similarly, standardised national-level estimates of vaccination coverage for each vaccine considered were provided by the VIMC secretariat to each group. Past coverage in all countries for 1980–2016 was obtained from WHO–UNICEF Estimates of National Immunization Coverage, as published in July, 2017.^17^ Future coverage estimates from 2017 to 2030 were based on Gavi's operational forecast as of October, 2017, for the countries eligible for Gavi support. Gavi's operational forecasts assume likely dates of vaccine introduction on the basis of non-binding expressions of interest from eligible countries, applications to Gavi for vaccine support, intended introductions as reported to WHO, and assessment of country capacity to introduce a specific vaccine in a specific timeframe. After introduction, coverage of new vaccines was typically assumed to reach coverage of a reference vaccine (eg, coverage of DTP3) within 2–3 years, after which coverage was assumed to increase by 1% per year up to a maximum of 90% or 95%, depending on the vaccine.^1^ For the 25 countries considered not supported by Gavi, and for years after 2030 for countries in Gavi's portfolio, an annual 1% increase in coverage was assumed from 2017 up to a maximum of 90% or the historic high coverage achieved (if >90%). For newly introduced vaccines with only an introduction date and no Gavi coverage forecast, we assumed that coverage would increase to the same coverage as pentavalent (hepatitis B, *H influenzae* type B, DTP3) vaccine in that country in the first 3 years and subsequently increase by 1% per year. Estimates of the number of vaccines received per child by year were generated from these coverage estimates and projections, assuming independence of coverage between vaccines.

### Procedures and outcomes

Disease burden was quantified by deaths and DALYs stratified by age. DALYs measure the years of healthy life lost due to premature death and disability from the disease. They are the sum of years of life lost through premature mortality and years lived with disability. No discounting or weighting was applied in the calculation of DALYs.

Age-stratified pathogen-specific disease burden estimates (deaths and DALYs) for annual birth cohorts between 2000 and 2030 were generated by each modelling group. Corresponding estimates for the counterfactual scenario, assuming no vaccination had occurred after 2000, were also produced. Supplementary immunisation activities, other immunisation campaigns (such as yellow fever reactive campaigns to outbreaks), and second doses were modelled when relevant. For rubella, only disease burden from congenital rubella syndrome^18^ was assessed, because rubella usually causes only mild disease in infected individuals.

The impact of vaccination was assessed by comparing the counterfactual scenario (no vaccination) with the reported and projected vaccination scenarios. Two forms of aggregation were used to present the results: by calendar year and by year of birth. The estimates by calendar year were used to assess the difference in burden between the reported and projected vaccination and no-vaccination scenarios for a specific year and to give a cross-sectional view of impact. The estimates by year of birth were used to sum disease burden across every year of life for each yearly birth cohort (born between 2000 and 2030), and also to assess the difference between reported and projected vaccination and no-vaccination scenarios, and therefore gives a lifetime view of vaccine impact. The population-attributable benefit of vaccination for each pathogen was estimated as the proportion of annual deaths due to each pathogen that would be prevented by vaccination.

We used model averaging to derive impact estimates, with each model for a pathogen given equal weighting. Uncertainty in model estimates was assessed by generating 200 sets of estimates from each model, probabilistically sampling over model parameter uncertainty. The same randomly sampled sets of parameters were used for both vaccination and no-vaccination model runs, allowing uncertainty in vaccine impact to be assessed. Central pathogen-specific estimates presented here represent averages over all such samples from every model available for each pathogen. The 95% credible intervals (CrIs; 2·5% and 97·5% quantiles) presented for each pathogen were derived by combining the probabilistic distributions of estimated impact from all the models available for that pathogen.

Estimates involving aggregation across pathogens were generated via a bootstrap approach under the simplifying assumption that the drivers of uncertainty in each model are independent of those in any other model. For each model, a random sample of the statistic of interest was drawn from the 200 probabilistic runs. These model-specific samples were combined by averaging them across models of the same pathogen and then summing the resulting pathogen-specific estimates across pathogens. Means and 2·5% and 97·5% quantiles of 100 000 such bootstrap samples were calculated to derive central estimates and 95% CrIs.

The main text of this Article focuses on presenting vaccine impacts on mortality; more detailed estimates of mortality impacts and estimates of DALYs averted by vaccination are provided in appendix 2 (pp 49–58).

### Role of the funding source

This report was compiled by all coauthors, including one coauthor from Gavi. Other funders had no role in study design, data collection, data analysis, data interpretation, or writing of the report. All authors had full access to all the data in the study and had final responsibility for the decision to submit for publication.

## Results

**Figure 1:**
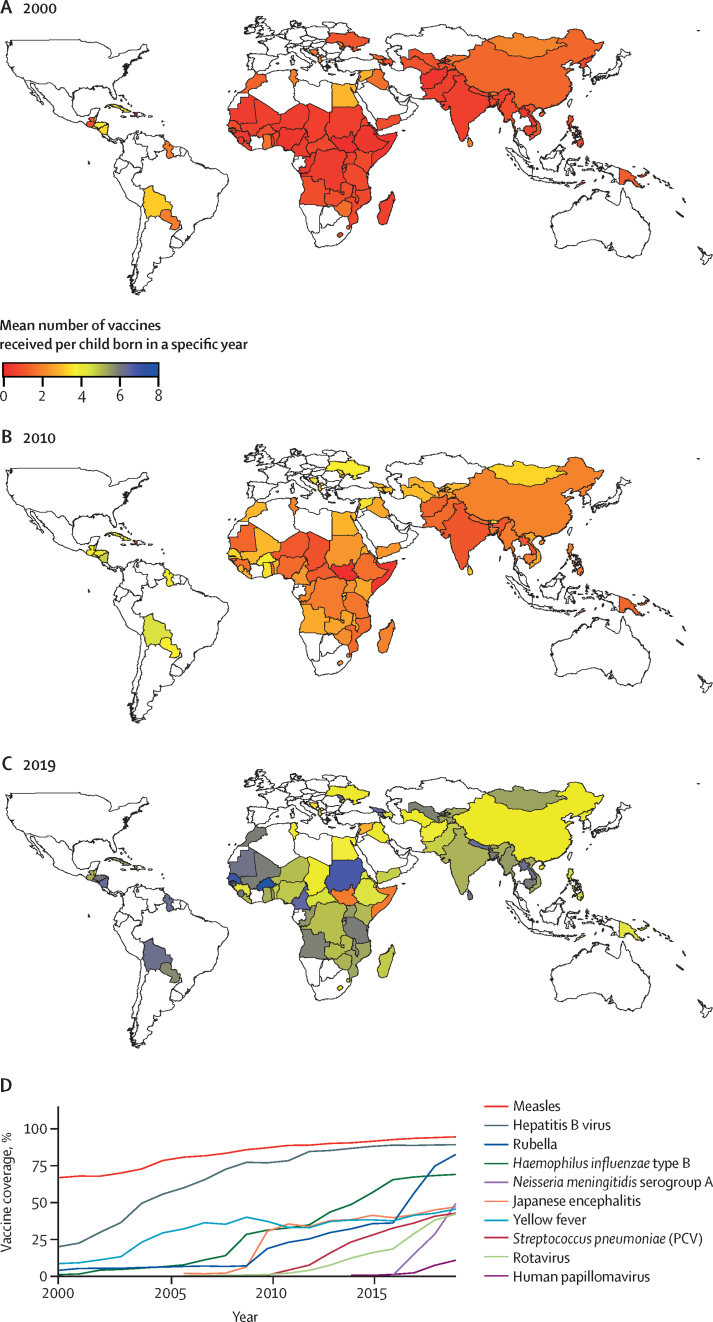
Vaccine coverage across the ten pathogens considered Vaccine coverage, calculated as the mean number of vaccines received per child born in a specific year, is shown for 2000 (A), 2010 (B), and 2019 (C). The colour scale shows the expected number of vaccines received per child in each country. (D) Routine vaccine coverage for each pathogen, from 2000 to 2019, averaged across all 98 countries, except for Japanese encephalitis, *Neisseria meningitidis* serogroup A, and yellow fever, which were averaged across the 16, 26, and 32 endemic countries for those pathogens, respectively. The average was obtained by dividing total vaccine doses by the total eligible population. PCV=pneumococcal conjugate vaccine.

In this study of the health impact of vaccination against ten pathogens, the average number of vaccines received per child increased across the majority of the 98 LMICs (appendix 2 pp 45–48) between 2000 and 2019 (figure 1A–C). Both increases in the coverage of existing vaccines (eg, measles-containing vaccines) and the introduction of new vaccines (eg, rotavirus vaccine) contribute to this overall trend (figure 1D). Routine vaccination against Japanese encephalitis and rotavirus began to be introduced from 2006, *S pneumoniae* (PCV) in 2010, HPV in 2014, and *N meningitidis* serogroup A in 2016. In addition, coverage increases also reflect the increase in countries eligible for Gavi support (appendix 2 p 48). The increase in vaccination is also reflected in the proportion of unvaccinated children (appendix 2 p 9) for the 2019 cohort. For different assumptions of the correlation of vaccine doses within and between different vaccines, the proportion of unvaccinated children in most of the countries was less than 0·1 (appendix 2 pp 5–9).

**Figure 2:**
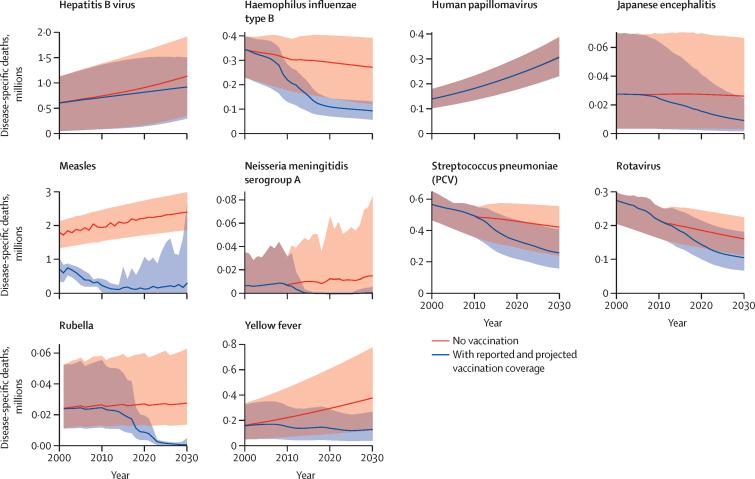
Estimates of disease-specific deaths by calendar year, from 2000 to 2030, across 98 countries, for reported and projected vaccine coverage and counterfactual (no vaccination) coverage Lines show estimates of deaths for the two scenarios, for all ages. The corresponding shaded areas show the 95% credible intervals (2·5% and 97·5% quantiles). The grey shaded parts show the area where the 95% credible intervals for the two scenarios overlap. PCV=pneumococcal conjugate vaccine.

To assess the impact of vaccination on mortality, we quantified the expected burden of disease in the counterfactual scenario of no vaccination and estimated the impact of observed and projected vaccination coverage on that baseline (figure 2). Depending on the pathogen considered, long-term trends in disease prevalence interact with global population growth and ageing, resulting in a variety of projected trends in disease-specific mortality in the counterfactual, no-vaccination scenario. However, for all except two pathogens (hepatitis B virus and HPV), vaccination between 2000 and 2030 was estimated to cause substantial reductions in mortality in the same time period. For hepatitis B virus and HPV, most mortality was due to infections that have already occurred, due to the typically long time delay between infection and severe outcomes for those infections. Although hepatitis B vaccination coverage is now relatively high and projected to increase further, most of the impact of this coverage will be seen only after 2030. For HPV, most countries have yet to introduce vaccination.

The age distribution of mortality varied considerably across the ten pathogens because of differences in their epidemiology. Mortality attributable to hepatitis B and HPV primarily affects those older than 40 years; yellow fever, *N meningitidis* serogroup A, and Japanese encephalitis are epidemic diseases that mostly affect those younger than 30 years (due to natural immunity acquired with age in older adults); mortality for all other pathogens was almost entirely focused in children younger than 5 years. Most of the mortality reduction from measles is attributable to the measles-containing vaccine, the first dose of which is not subject to Gavi funding.

**Figure 3:**
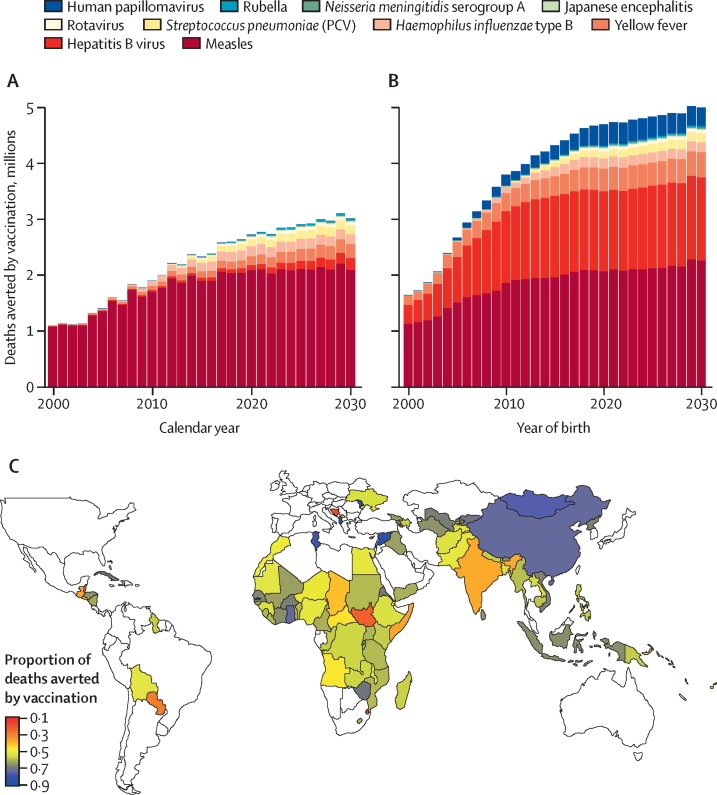
Estimates of deaths averted by vaccination in 98 countries (A) Estimates of death averted by calendar year (summing across all ages) and pathogen. (B) Estimates of deaths averted by year of birth (summing across lifetime) and pathogen. (C) Proportion of lifetime deaths due to the ten pathogens considered in the no-vaccination counterfactual scenario that are predicted to be averted by vaccination, by country, across 2000–19 birth cohorts. PCV=pneumococcal conjugate vaccine.

The estimated numbers of deaths averted by vaccination in the 98 countries are presented in figure 3 and appendix 3 (p 1). Estimates of DALYs averted are presented in appendix 2 (p 65). Estimated numbers of deaths and DALYs averted among children younger than 5 years are also presented in appendix 2 (p 66).

In terms of deaths averted by calendar year (figure 3A), 69 million (95% CrI 52–88) deaths were estimated to be averted between 2000 and 2030, of which 37 million (30–48) were averted between 2000 and 2019 (table 2). In the 73 Gavi countries, the corresponding values were 66 million (49–83) deaths averted between 2000 and 2030, 35 million (28–45) of which were averted between 2000 and 2019 (appendix 2 p 53). Of the ten pathogens, vaccination against measles has the largest impact, with 56 million (39–74) deaths averted between 2000 and 2030 (54 million [37–70] in the 73 Gavi countries).

**Table 2:** Estimated total deaths averted by vaccination and deaths averted per thousand individuals vaccinated in 98 countries, by time period and pathogen

	**Deaths averted, 1000s**	**Deaths averted per 1000 vaccinated individuals**	**Deaths averted in children <5 years, 1000s**	**Deaths averted per 1000 vaccinated children <5 years**
**Hepatitis B virus**				
2000–19	640 (190–1500)	0·4 (0·1–0·9)	270 (51–1100)	0·2 (0–0·6)
2020–30	1600 (460–2900)	1·0 (0·3–1·8)	230 (59–810)	0·1 (0–0·5)
2000–30	2200 (660–3700)	0·7 (0·2–1·1)	500 (110–1900)	0·1 (0–0·5)
Haemophilus influenzae **type B**				
2000–19	1600 (720–2400)	2·5 (1·1–3·7)	1600 (720–2400)	2·5 (1·1–3·7)
2020–30	2000 (730–3200)	2·4 (0·9–3·7)	2000 (730–3200)	2·4 (0·9–3·7)
2000–30	3600 (1400–5500)	2·4 (1·0–3·7)	3600 (1400–5500)	2·4 (1·0–3·7)
**Human papillomavirus**				
2000–19	0·013 (0–0·031)	0	0	0
2020–30	7·4 (1·4–15·0)	0	0	0
2000–30	7·4 (1·4–15·0)	0	0	0
**Japanese encephalitis**				
2000–19	75 (6·1–190)	0·2 (0–0·4)	52 (4·2–130)	0·2 (0–0·4)
2020–30	160 (16–420)	0·4 (0–0·9)	86 (8·1–230)	0·2 (0–0·5)
2000–30	240 (22–600)	0·3 (0–0·6)	140 (12–350)	0·2 (0–0·5)
**Measles**				
2000–19	33 000 (26 000–44 000)	6·9 (5·5–9·2)	32 000 (26 000–43 000)	7·9 (6·4–11)
2020–30	23 000 (9400–31 000)	9·1 (3·7–12·0)	23 000 (10 000–31 000)	9 (4–12)
2000–30	56 000 (39 000–74 000)	7·7 (5·4–11·0)	55 000 (40 000–73 000)	8·3 (6–11)
Neisseria meningitidis **serogroup A**				
2000–19	73 (12–160)	0·3 (0–0·6)	19 (2–38)	0·1 (0–0·3)
2020–30	140 (52–270)	0·5 (0·2–1·1)	33 (11–59)	0·1 (0–0·2)
2000–30	210 (130–360)	0·4 (0·3–0·7)	52 (32–81)	0·1 (0·1–0·2)
Streptococcus pneumoniae **(PCV)**				
2000–19	610 (320–1100)	2·5 (1·3–4·2)	610 (320–1100)	2·5 (1·3–4·2)
2020–30	1600 (730–2900)	2·3 (1·0–4·1)	1600 (730–2900)	2·3 (1·0–4·1)
2000–30	2200 (1000–4000)	2·3 (1·1–4·1)	2200 (1000–4000)	2·3 (1·1–4·1)
**Rotavirus**				
2000–19	150 (100–200)	0·8 (0·6–1·1)	150 (100–200)	0·8 (0·6–1·1)
2020–30	590 (370–820)	0·8 (0·5–1·1)	590 (370–820)	0·8 (0·5–1·1)
2000–30	740 (470–1100)	0·8 (0·5–1·1)	740 (470–1100)	0·8 (0·5–1·1)
**Rubella**				
2000–19	80 (38–200)	0·1 (0–0·1)	80 (38–200)	0·1 (0–0·2)
2020–30	260 (130–590)	0·1 (0·1–0·3)	260 (130–590)	0·1 (0·1–0·3)
2000–30	340 (180–780)	0·1 (0–0·2)	340 (180–780)	0·1 (0·1–0·2)
**Yellow fever**				
2000–19	1300 (450–2800)	3·5 (1·1–7·1)	500 (150–1100)	2·3 (0·7–4·9)
2020–30	2300 (740–4800)	9·0 (2·8–19·0)	510 (150–1100)	2 (0·6–4·2)
2000–30	3600 (1100–7500)	5·7 (1·8–12·0)	1000 (310–2200)	2·1 (0·7–4·5)
**Total**				
2000–19	37 000 (30 000–48 000)	3·7 (3·0–4·7)	36 000 (29 000–46 000)	4·1 (3·2–5·2)
2020–30	32 000 (17 000–41 000)	3·2 (1·8–4·0)	28 000 (15 000–36 000)	2·9 (1·6–3·7)
2000–30	69 000 (52 000–88 000)	3·4 (2·6–4·3)	64 000 (48 000–82 000)	3·5 (2·7–4·4)

Values are mean estimates (95% credible intervals). Corresponding estimates for disability-adjusted life-years averted are presented in appendix 2 (p 49). PCV=pneumococcal conjugate vaccine.

Considering deaths averted by birth cohort (figure 3B, appendix 2 p 50), the longer-term impact of increasing coverage of hepatitis B vaccination becomes clearer. Because the majority of hepatitis B deaths (due to liver disease) occur in those older than 45 years, the impact of vaccination will not be seen in the 2000–30 time period (figure 3A) but will start to be seen from 2040 onwards. Similar arguments apply to HPV vaccination (although coverage is substantially lower than for hepatitis B); cervical cancer deaths largely occur in women older than 50 years, leading to a delay of nearly 40 years between vaccination and its direct impact on mortality. Thus, summing vaccination impact over the full lifetimes of the 2000–30 birth cohorts gives a total of 120 million (95% CrI 93–150) estimated deaths averted, of which 65 million (48–83) are in children younger than 5 years. In the 73 Gavi countries, the corresponding values are 100 million (78–130) deaths averted in the 2000–30 birth cohorts, of which 62 million (46–78) of which are in children younger than 5 years (appendix 2 p 55). In estimates of deaths averted by pathogen in the 2000–30 birth cohorts, more than 53% (45–66) of deaths averted are in children under 5 years (appendix 2 p 50).

The extent to which vaccination reduces overall mortality due to the ten pathogens varies substantially by country (figure 3C), largely due to historical variations in vaccination coverage, but also due to variation in the epidemiology of some pathogens by country. We estimate vaccination will prevent 72% (95% CrI 59–81) of the mortality associated with these ten pathogens in the 2019 annual birth cohort across the countries considered. This proportion rises to 76% (54–84) if only mortality of children younger than 5 years is considered. In the 73 Gavi countries, the corresponding values are a 72% (57–79) reduction in all-age mortality and a 76% (54–84) reduction in mortality among children younger than 5 years.

In the period 2000–19, we estimate that vaccination in the 98 countries reduced overall mortality due to these pathogens by 45% (95% CrI 36–58) and mortality among children younger than 5 years by 57% (52–66). In the 73 Gavi countries, these reductions were 48% (39–59) and 57% (53–66), respectively. For the period 2020–30, we project these reductions will increase to 60% (33–74) for overall mortality and 77% (44–86) for mortality among children younger than 5 years in all 98 countries (64% [35–76] and 78% [44–86], respectively, in the 73 Gavi countries).

It is informative to place these estimates in their demographic context. Taking the 2019 birth cohort as an example, and using UNWPP demographic estimates, we estimate that mortality among children younger than 5 years in the 98 countries would be 45% (95% CrI 31–57) higher in the absence of any vaccination against the ten pathogens. The total number of deaths (occurring at any age) averted in the 2019 birth cohort represent 4·1% (3·2–5·0) of the live births in that cohort.

Total impact reflects vaccination coverage as well as underlying disease burden and vaccine effectiveness. We therefore examine the relative impact of each vaccine, by quantifying deaths averted per vaccinated individual for each pathogen (table 2, appendix 2 p 50, appendix 3 p 2). Although vaccines against measles, *H influenzae* type B, and *S pneumoniae* have the largest relative impact on mortality of children younger than 5 years, vaccines against HPV, hepatitis B virus, and yellow fever have the largest impact per person vaccinated by year of birth. Vaccines against measles and yellow fever have the largest impact per person vaccinated by calendar year. Yellow fever and HPV vaccination have the largest relative impact of all the vaccines considered, with central estimates for both of over 16 (95% CrI 5–32) deaths averted per 1000 persons vaccinated for the 2000–19 birth cohorts.

Because most of the pathogens considered (*H influenzae* type B, Japanese encephalitis, measles, *S pneumoniae,* rotavirus, and rubella) result in mortality of children younger than 5 years, the impact of vaccination on DALYs largely mirrors the impact on mortality (appendix 2 p 66). Because deaths related to hepatitis B and HPV are focused in individuals older than 50 years, mortality contributes fewer years of life lost for these pathogens, but morbidity contributes higher years lived with disability for both infections (particularly hepatitis B). Estimates of vaccine impact in DALYs averted are presented in appendix 2 (pp 49–58).

## Discussion

In this study, the health impact of immunisation in LMICs is estimated on the largest scale to date, covering vaccination programmes against ten pathogens and evaluating impact in 98 countries. It represents an advance on previous work^1^ in terms of scale (the number of pathogens and countries considered) and in the emphasis of VIMC on standardising model inputs (vaccine coverage and demography) and outputs (age-specific mortality and DALYs by year of birth cohort and age). Such standardisation allows impacts to be combined across, and compared between, vaccines.

Our analysis highlights where the greatest gains from future investments in improving vaccine coverage are to be made. We predict increasing HPV coverage in girls will avert more deaths per person vaccinated than any other immunisation activity, whereas increasing PCV coverage will give the largest reductions in mortality among children younger than 5 years.

We find that immunisation programmes in the 98 countries considered will result in individuals born in 2019 experiencing 72% lower mortality due to those ten pathogens over their lifetime than they would with no immunisation. Furthermore, in the absence of vaccination, we estimate that all-cause mortality among children younger 5 years would be 45% higher than currently observed. These impacts are a testament to both the public health benefit of vaccines overall and the sustained investment in increasing global vaccination coverage in the past two decades. They also highlight what might be lost if current vaccination programmes are not sustained, and thus provide quantitative evidence supporting both donor and country investments in vaccination programmes.

When compared with mortality estimates for measles from the Global Burden of Disease (GBD) Study 2017, we find some differences between 2000 and 2010. These are likely to be driven by differences in source data and model types. For example, the GBD measles estimates are based on static models, whereas the two VIMC models are both dynamic. Further comparison is provided in appendix 2 (pp 10–12).

Deriving these impact estimates is far from straightforward. Cause-specific mortality data in the LMICs considered is not widely available, making direct observational assessment of impact challenging. However, to inform monitoring and decision-making, countries and international organisations such as Gavi require projections of potential impact under a range of investment scenarios. To fill these gaps, mathematical and statistical models can be used to extrapolate data on levels of current infection (eg, case detection from active surveillance), past infection (eg, serosurveys), or both, to sites and countries without such data. These models can also be used to project future trends given information about vaccine coverage.

In addition, our study has focused on quantifying uncertainty in vaccine impact. Given the scarce explicit data on pathogen-specific disease burden available in many of the 98 countries considered, nearly all models need to extrapolate from settings where data are available to those where data are absent. This, together with imperfect knowledge of aspects of the epidemiology of each pathogen (eg, case-fatality ratios, transmissibility, disease progression rates) means that uncertainty in vaccine impact estimates from a single model can be substantial. Here, this uncertainty is quantified probabilistically, with each modelling group providing 200 model runs spanning the range of parametric uncertainty in their models.

A second source of uncertainty is structural; different modelling groups make different subjective choices about how to represent disease epidemiology and might use different data for model parameterisation. In addition, the models within VIMC vary substantially in their type (static cohort models *vs* transmission-dynamic models), in their complexity (eg, in the representation of age effects), and in their approaches to calibration and validation (from formal statistical likelihood approaches to more ad-hoc calibration). VIMC therefore includes at least two models for each pathogen (with the exception of yellow fever) and combines results from different models to derive central estimates of impact and to better quantify underlying uncertainty.

A limitation of our current analysis is that we do not currently evaluate uncertainty in demographic estimates and estimates of past and future vaccine coverage. Developing principled approaches to doing so is a topic of current research, but is made challenging by the paucity of information available on uncertainty in UNWPP demographic estimates and in WHO–UNICEF Estimates of National Immunization Coverage and Gavi operational forecast vaccine coverage estimates.

In addition, our study has only focused on 98 LMICs. The countries considered here have the highest burden from the ten pathogens considered (appendix 3 p 5). Therefore, there has been a greater focus on supporting vaccine introduction and implementation in these countries, mainly through Gavi (appendix 3 p 4). These 98 countries include the 73 countries eligible for Gavi support^1^ and 25 other countries that are of interest to the funders.

For most vaccines not yet introduced in some countries, we assume that once the vaccine were to be introduced in that country, the coverage would reach the same coverage of a reference vaccine (eg, DTP3) in 2–3 years. However, in some countries, there have been substantial delays in implementing the vaccine due to shortages in supply,^19^ and some countries, such as India, have initially introduced the vaccine in a few states to assess the feasibility of a new oral vaccine into their programme.^20^ Therefore, the use of a reference vaccine for rotavirus vaccination coverage for countries that have had problems or delays in introduction and scale-up might have led to an overestimation of impact.

The majority of models within VIMC adopt a bottom-up approach to modelling disease burden and thus the impact of vaccination. These models represent time-varying and age-varying pathogen-specific infection or disease rates in each country, and model mortality as affecting a fraction of those infected by applying a case-fatality ratio (generally estimated from a combination of longitudinal epidemiological studies and surveillance data) to resulting case incidence estimates. Because the disease burden attributed to each pathogen is modelled separately, there is a theoretical risk of overestimating deaths due to a failure to account for competing causes of mortality, particularly in children younger than 5 years, in whom mortality is concentrated. However, in the 2019 annual birth cohort, UNWPP projections estimate all-cause mortality among children younger than 5 years to be 4·9% for the 98 countries considered. We estimate the ten pathogens considered in this study will cause approximately a seventh of this (0·7% [95% CI 0·5–1·4%] of all-cause mortality). With such low absolute proportions, the effect of competing hazards of death on overall mortality estimates is negligible. Conversely, when assessing deaths averted, we consider the counterfactual scenario of no vaccination for each vaccine antigen separately, subsequently summing across all vaccines, because one child's life can be saved multiple times.

For most pathogens, we currently model infection risk as homogeneous within individual countries (with the exceptions of the yellow fever, *N meningitidis* serogroup A, and Japanese encephalitis models). Furthermore, no models in this study account for geographic or socioeconomic clustering of vaccine coverage, or for any potential correlation between access to health care (including vaccines) and disease risk. Thus, we may not be accounting for disadvantaged subpopulations in countries with lower than average access to vaccines, higher than average intrinsic exposure to infection, or both. Subnational stratification of vaccine impact estimates is a priority for future work but require similarly fine-grained estimates of vaccine coverage^21^ and disease burden.

Last, in making long-term projections of disease burden and intervention impact, it is necessary to make assumptions about the likely improvements in treatment and disease outcomes in future decades. This is particularly relevant for hepatitis B and HPV, for which cancer screening and treatment services can make a substantial difference to disease-related mortality,^22^ and for measles, for which decreasing background mortality among children younger than 5 years can significantly reduce case-fatality ratios.^23^ The HPV and hepatitis B models included in VIMC currently make conservative (ie, relatively pessimistic) assumptions about improvements in cancer screening and treatment in low-income countries.

More generally, the estimates provided here should not be viewed as immutable; our understanding of the epidemiology and disease burden caused by all ten pathogens continues to improve, and models of those diseases should likewise continue to be refined. In addition, future vaccine coverage is unlikely to precisely match the coverage projections used here. Thus, the estimates of the impact of past immunisation activities and projections of future impact will also change. However, the results in this paper provide the most comprehensive and definitive assessment to date of the impact of the dramatic advances in immunisation coverage in LMICs in the last two decades.

Finally, it is crucial to increase vaccine coverage and maintain high coverage levels in all countries to avoid the coverage gains achieved since 2000 being undone. This effort requires continued political commitment, funding, civil society engagement (in promoting vaccine benefits and countering vaccine hesitancy), improving public trust and confidence in the safety and efficacy of vaccines,^24^ and strengthening immunisation programmes through education, training, and supervision.^25^

## Data sharing

This study does not involve any patient data or participant data. Data collected for the study consisted of demographic data from UNWPP and coverage data from WHO–UNICEF Estimates of National Immunization Coverage, which are freely accessible. Further coverage projections were obtained from the Gavi operational forecast, which is prepared to provide an aggregate, long-term strategic picture of the portfolio of Gavi vaccines and, as such, is highly uncertain at the country level. Due to programmatic reasons, the operational forecast is sensitive. As such, interested parties should contact Hope L Johnson (hjohnson@gavi.org) for more information. Estimates of vaccine impact and disease burden for 2000–19 (specified as deaths, deaths averted, DALYs, and DALYs averted) are presented in a publicly available data visualisation tool.

## Supplementary Materials

French translation of the abstract
