# Department of Error

**DOI:** 10.1016/S0140-6736(21)00398-6

**Published:** 2021-02-20

*Li X, Mukandavire C, Cucunubà ZM, et al. Estimating the health impact of vaccination against ten pathogens in 98 low-income and middle-income countries from 2000 to 2030: a modelling study.* Lancet *2021; **397:** 398–408*—In appendix 2, the table legends for appendix 3 p 1 (d–f) and p 2 (d–f) should have been for under-5s by calendar view, and the table legends for appendix 3 p 1 (g–i) and p 2 (g–i) should have been for all ages by cohort view. The titles of the tables have also been corrected. These corrections have been made as of Feb 18, 2021.

