# Supplementary material for: Estimating the health impact of vaccination against ten pathogens in 98 low-income and middle-income countries from 2000 to 2030: a modelling study
Source: Lancet. 2021 Jan 30;397(10272):398–408. doi: 10.1016/S0140-6736(20)32657-X (PMC7846814; doi:10.1016/S0140-6736(20)32657-X)
Supplement: French translation of the abstract [file mmc1.pdf]

THE LANCET

Supplementary appendix

This appendix formed part of the original submission and has been peer reviewed. We post it as supplied by the authors.

Supplement to: Li X, Mukandavire C, Cucunubá ZM, et al. Estimating the health impact of vaccination against ten pathogens in 98 low-income and middle-income countries from 2000 to 2030: a modelling study. *Lancet* 2021; **397:** 398–408.

Estimation de l’impact sanitaire de la vaccination contre dix agents pathogènes dans 98 pays à revenu faible et intermédiaire entre 2000 et 2030

Résumé

Contexte

Au cours des deux dernières décennies, les programmes de vaccination infantile se sont généralisés dans les pays à revenu faible ou intermédiaire (PRFI). Nous quantifions ici l’impact sanitaire de ces programmes en estimant les décès et le nombre d'années de vie corrigées de l’incapacité (AVCI) évités grâce à la vaccination contre dix agents pathogènes dans 98 PRFI entre 2000 et 2030.

Méthodes

16 groupes de modélisation indépendants ont fourni des estimations des conséquences des maladies causées par dix agents pathogènes dans le cadre de plusieurs scénarios de couverture vaccinale : virus de l’hépatite B (HepB), *Haemophilus influenzae* type b, papillomavirus humain, encéphalite japonaise, rougeole, *Neisseria meningitidis* de sérogroupe A, *Streptococcus pneumoniae*, rotavirus, rubéole, et fièvre jaune. À l’aide de données standardisées de démographie et de couverture vaccinale, l'impact des programmes de vaccination a été déterminé en comparant les estimations modélisées à partir d’un scénario contrefactuel sans vaccination avec celles provenant d’un scénario basé sur des niveaux de vaccination rapportés et prévus. Les décès et AVCI évités de 2000 à 2030 sont présentés par année calendaire et par cohorte de naissance annuelle.

Résultats

Nous estimons que la vaccination pour les 10 pathogènes étudiés aura permis d’éviter 69 (intervalle de crédibilité à 95% : 52–88) millions de décès de 2000 à 2030, dont 37 millions (30–48) évités entre 2000 et 2019. Sur la période 2000-2019, cela représente une baisse de 45% (36–58) des décès par rapport à un scénario contrefactuel sans vaccination. L’essentiel de ces bénéfices est concentré dans la réduction de mortalité des enfants de moins de 5 ans (57% de réduction [52–66]), en particulier due à la rougeole. Au cours de la vie des cohortes nées entre 2000 et 2030, nous prévoyons que 120 millions (93 - 150) de décès seront évités grâce à la vaccination, dont 58 millions (39–76) grâce à la vaccination contre la rougeole et 38 millions (25–52) grâce à la vaccination contre l’hépatite B, respectivement. Nous estimons que les augmentations de la couverture vaccinale et l’introduction d’autres vaccins entraîneront une baisse de 72 % (59–81) de la mortalité tout au long de la vie pour la cohorte née en 2019.

Interprétation

Les augmentations de la couverture vaccinale et l’introduction de nouveaux vaccins dans les PRFI ont eu un impact majeur sur la baisse de la mortalité. Ces bénéfices de santé publique devraient s'accroître dans les décennies à venir si la progression de la couverture vaccinale se poursuit.
