# Supplementary material for: Estimating the health impact of vaccination against ten pathogens in 98 low-income and middle-income countries from 2000 to 2030: a modelling study
Source: Lancet. 2021 Jan 30;397(10272):398–408. doi: 10.1016/S0140-6736(20)32657-X (PMC7846814; doi:10.1016/S0140-6736(20)32657-X)
Supplement: Supplementary appendix 2 [file mmc2.pdf]

THE LANCET

Supplementary appendix

This appendix formed part of the original submission and has been peer reviewed. We post it as supplied by the authors.

Supplement to: Li X, Mukandavire C, Cucunubá ZM, et al. Estimating the health impact of vaccination against ten pathogens in 98 low-income and middle-income countries from 2000 to 2030: a modelling study. *Lancet* 2021; **397:** 398–408.

Supplementary Information

Estimating the health impact of vaccination against 10 pathogens in 98 low and middle income countries from 2000 to 2030

Authors

Xiang Li[1], Christinah Mukandavire[1], Zulma M Cucunubá, Susy Echeverria Londono, Kaja Abbas[2], Hannah E Clapham[2], Mark Jit[2], Hope L Johnson[2], Timos Papadopoulos[2], Emilia Vynnycky[2], Marc Brisson[3], Emily D Carter[3], Andrew Clark[3], Margaret J de Villiers[3], Kirsten Eilertson[3], Matthew J Ferrari[3], Ivane Gamkrelidze[3], Katy A M Gaythorpe[3], Nicholas C Grassly[3], Timothy B Hallett[3], Wes Hinsley, Michael L Jackson[3], Kévin Jean[3], Andromachi Karachaliou[3], Petra Klepac[3], Justin Lessler[3], Xi Li[3], Sean M Moore[3], Shevanthi Nayagam[3], Duy Manh Nguyen[3], Homie Razavi[3], Devin Razavi-Shearer[3], Stephen Resch[3], Colin Sanderson[3], Steven Sweet[3], Stephen Sy[3], Yvonne Tam[3], Hira Tanvir[3], Quan Minh Tran[3], Caroline L Trotter[3], Shaun Truelove[3], Kevin van Zandvoort[3], Stéphane Verguet[3], Neff Walker[3], Amy Winter[3], Kim Woodruff, Neil M Ferguson[4], Tini Garske (Vaccine Impact Modelling Consortium)

MRC Centre for Global Infectious Disease Analysis, Abdul Latif Jameel Institute for Disease and Emergency Analytics (J-IDEA), School of Public Health, (Xiang Li PhD, C Mukandavire PhD, Z M Cucunubá MD PhD, S Echeverria Londono PhD, M J de Villiers PhD, K A M Gaythorpe PhD, N C Grassly DPhil, T B Hallett PhD, W Hinsley PhD, K Jean PhD, S Nayagam PhD, K Woodruff, N M Ferguson DPhil, T Garske PhD), and Section of Hepatology & Gastroenterology, Department of Metabolism, Digestion and Reproduction (S Nayagam PhD), Imperial College London, London, UK; London School of Hygiene & Tropical Medicine (K Abbas PhD, M Jit PhD, A Clark PhD, P Klepac PhD, C Sanderson PhD, H Tanvir, K van Zandvoort, E Vynnycky PhD); Saw Swee Hock School of Public Health, National University of Singapore (H E Clapham PhD); Gavi, the Vaccine Alliance (H L Johnson PhD MPH); Public Health England (T Papadopoulos PhD, E Vynnycky PhD, M Jit PhD); Laval University (M Brisson PhD); Department of Epidemiology, Bloomberg School of Public Health (J Lessler PhD, S Truelove PhD, A Winter PhD), and Department of International Health, Bloomberg School of Public Health, (E D Carter PhD, Y Tam MHS, N Walker PhD), Johns Hopkins University; Colorado State University (K Eilertson PhD); The Pennsylvania State University (M J Ferrari PhD); Center for Disease Analysis Foundation, Lafayette, Colorado, USA (I Gamkrelidze, H Razavi PhD, D Razavi-Shearer); Kaiser Permanente Washington (M Jackson PhD); University of Cambridge (A Karachaliou Mmath, C L Trotter BA MSc PhD); Unaffiliated (Xi Li); Department of Biological Sciences, University of Notre Dame (S Moore PhD, Q M Tran MD); School of Computing, Dublin City University (D M Nguyen); Harvard T.H. Chan School of Public Health (S Resch PhD, S Sweet MSM, S Sy MS); Department of Global Health and Population, Harvard T.H. Chan School of Public Health (S Verguet PhD); Center for

[1] Contributed equally (current VIMC science team)
[2] Contributed equally (writing group)
[3] Contributed equally
[4] Corresponding author. Email: neil.ferguson@imperial.ac.uk; MRC Centre for Global Infectious Disease Analysis, Abdul Latif Jameel Institute for Disease and Emergency Analytics (J-IDEA), School of Public Health, Imperial College London, Norfolk Place, London, W2 1PG, UK

Health Decision Science, Harvard T.H. Chan School of Public Health, Harvard University (S Resch PhD, S Sweet MSM, S Sy MS); Oxford University Clinical Research Unit (H E Clapham PhD, D M Nguyen, Q M Tran MD); University of Southampton (T Papadopoulos PhD); Laboratoire MESuRS, Conservatoire National des Arts et Métiers, Paris, France (K Jean PhD); Unité PACRI, Institut Pasteur, Conservatoire National des Arts et Métiers, Paris, France (K Jean PhD); University of Hong Kong (M Jit PhD); Nuffield Department of Medicine (H E Clapham PhD)

Between and within variation of models in impact estimates

To measure the degree of divergence of impact estimates between models, we decompose the total variation of impact estimates into between model variation and within model variation using the sum of squares method.

Let s_o^2 represent the total variance of impact estimates from all models for a specific pathogen. Then, denote $\{x_{1m}, x_{2m}, \ldots, x_{Nm}\}$ as the sequence of impact estimates of stochastic samples in the model m. The total variance of impact estimates across stochastic samples N and across the different models M is therefore defined as

$$s_o^2 = \frac{1}{NM} \sum_i \sum_m (x_{im} - \overline{x})^2$$

We describe the between model variance s_B^2 as the amount of deviation of the model mean $\overline{x}_m$ from the total mean $\overline{x}$ (from all models of the same pathogen) which is denoted as

$$s_B^2 = \frac{1}{M} \sum_m (\overline{x}_m - \overline{x})^2$$

The within variance s_W^2 defined as the amount of deviation of the stochastic samples impact estimates x_{im} from the model mean $\overline{x}_m$ is therefore denoted as

$$s_W^2 = \frac{1}{NM} \sum_i \sum_m (x_{im} - \overline{x}_m)^2$$

Given that $s_o^2 \approx s_B^2 + s_W^2$, we calculate the proportion of between model variance P_B^2 as

$$P_B^2 = \frac{s_B^2}{s_o^2}$$

If the impact estimates between models do not differ greatly from each other, the proportion of between model variation P_B^2 will be small (i.e., the within model variation will approach the total variation of impact estimates). Since $s_o^2 \approx s_B^2 + s_W^2$, the proportion of within model variation can be calculated as 1-P_B^2. The proportions of between model variation for each pathogen are presented in Table S1.

A total of 20 pathogen-specific mathematical models were used by VIMC to produce the vaccine impact estimates presented here: two models for each pathogen other than HepB, which had three, and yellow fever, which had one. Including multiple models for each pathogen enables some assessment of the impact of structural uncertainty in models

Table (i): Proportion of between model variation of impact estimates across the 98 countries considered for calendar view and lifetime view. Both all ages and under-5s are shown. There are two models for each pathogen, except for HepB which has three and yellow fever which has one.

Disease	Period	P_B^2 Calendar view		P_B^2 lifetime view	
		All	<5	All	<5
HepB	2000-2019	0.87	0.85	0.73	0.85
	2020-2030	0.93	0.85	0.84	0.85
	2000-2030	0.91	0.85	0.75	0.85
Hib	2000-2019	0.97	0.97	0.98	0.98
	2020-2030	0.99	0.99	0.99	0.99
	2000-2030	0.98	0.98	0.98	0.98
HPV	2000-2019	0.98	NA	0.32	NA
	2020-2030	0.99	NA	0.03	NA
	2000-2030	0.99	NA	0.06	NA
JE	2000-2019	0.04	0.04	0.01	0.03
	2020-2030	0.01	0.01	0	0.01
	2000-2030	0.02	0.02	0.01	0.02
Measles	2000-2019	0.09	0.08	0.01	0.02
	2020-2030	0.03	0.02	0.03	0.02
	2000-2030	0	0	0.01	0
MenA	2000-2019	0.11	0.01	0.97	0.04
	2020-2030	0.34	0.08	0.98	0.09
	2000-2030	0.5	0.11	0.98	0.14
PCV	2000-2019	0.57	0.57	0.62	0.62
	2020-2030	0.72	0.72	0.73	0.73
	2000-2030	0.68	0.68	0.7	0.7
Rota	2000-2019	0.06	0.06	0.36	0.36
	2020-2030	0.25	0.25	0.3	0.3
	2000-2030	0.21	0.21	0.31	0.31
Rubella	2000-2019	0.39	0.39	0.39	0.39
	2020-2030	0.38	0.38	0.38	0.38
	2000-2030	0.38	0.38	0.38	0.38
YF	2000-2019	0	0	0	0
	2020-2030	0	0	0	0
	2000-2030	0	0	0	0

Method accounting for coverage clustering and double counting of disease burden

It is vital to account for double counting when measuring the total vaccine impact across all diseases modelled. The approach adopted in this paper is to account for double counting of mortality or morbidity by adjusting the estimated disease burden and the vaccine impact at population or cohort clustering according to the number of pathogens against which people are protected.

Absolute vaccine coverage

For any given country, we cluster the population into categories defined by the number of pathogens against which they are protected. We conduct this clustering based on the absolute vaccine coverage. Two aspects of absolute vaccine coverage levels are considered: absolute vaccine coverage by calendar year, and absolute vaccine coverage by year of birth.

Let T and C be time and birth cohort, $K = \{1, \ldots, 10\}$ be the set of the ten pathogens studied in this paper and let D_k be the vaccination schedule for pathogen k. Define $v_k^0(c, t, d_k)$ as coverage of any particular vaccination activity d_k against pathogen k in year t targeting birth cohort c, where $k \in K$, $t \in T$, $c \in C$ and $d_k \in D_k$.

The total vaccination coverage $v_k^0(c, t)$ is defined as a product of $v_k^0(c, t, d_k)$ depending on vaccination schedule D_k, i.e.

$$v_k^0(c,t) = 1 - \left(1 - max_{d_{k,i} \in routine} v_k^0(c, t, d_{k,i})\right) \prod_{d_{k,j} \in campaign} \{1 - v_k^0(c, t, d_{k,j})\}$$

Assuming 0% (no) correlation for vaccine doses against the same pathogen given at different times t, then, absolute vaccine coverage for birth cohort c in year t against pathogen k is derived as

$$v_k(c,t) = 1 - \prod_{i \leq t} \left(1 - v_k^0(c, i)\right) \qquad (1)$$

Let $P(c, t)$ be population size for of birth cohort c in year t. Then absolute vaccine coverage against pathogen k in year t is defined as

$$v_k(t) = \frac{\sum_{i \in C} v_k(i,t) P(i,t)}{\sum_{i \in C} P(i,t)},$$

and the absolute vaccine coverage against pathogen k for birth cohort c is defined as

$$v_k(c) = v_k\left(c, max(t)\right).$$

Clustering of population/cohort by absolute vaccine coverage

For any country, we clustered the population/cohort by the number of pathogens against which they are protected. Here we assumed that the correlation among vaccine doses against different pathogens is 100%. This means that people who have received more vaccines against different pathogens will be allocated the next vaccine against a different pathogen prior to other people who have received fewer vaccines.

Let $\mathring{v}_i$ represents the absolute vaccine coverage of people who have received i vaccines against i pathogens. Then, denote $\{\mathring{v}_1(t), \mathring{v}_2(t), \ldots, \mathring{v}_N(t)\}$ as the sequence of positive absolute vaccine coverage values in increasing order in year t; and $\{\mathring{v}_1(c), \mathring{v}_2(c), \ldots, \mathring{v}_N(c)\}$ as the sequence of positive absolute vaccine coverage values in increasing order for cohort c in year t. For simplicity of presentation, set $\mathring{v}_0 = 1$ and $\mathring{v}_{N+1} = 0$. Therefore, the proportion of people that has received i number of vaccines is

$$\rho_i = \mathring{v}_i - \mathring{v}_{i+1} \qquad (2)$$

Disease burden among vaccinated population

To measure vaccine impact, we use estimates of disease burden (deaths and disability-adjusted life years (DALYs)) from the counterfactual (no vaccination) scenario and the observed and projected vaccination scenario. However, mortality and morbidity from the observed and projected vaccination scenario include disease burden from both the vaccinated and unvaccinated populations. To account for double counting of disease burden, we therefore disentangle disease burden among the vaccinated and unvaccinated populations.

Denote burden (deaths or DALYs) from disease i from the counterfactual and the observed and projected vaccination scenarios as H_i^0 and H_i^d, respectively; and further denote as $P_{lb}(c)$ the number of live births, $P(a,t)$ the population size at age *a* and time t, $L_e(c)$ the life expectancy at birth for cohort c, and $L_y(a,t)$ the remaining life years by age and time.

The proportion of mortality (deaths) in the counterfactual scenario and that in the observed and projected vaccination scenario in year t are

$$M^0{}_i(t) = \frac{H_i^0(t)}{\sum P(a,t)} \text{ and } M^d{}_i(t) = \frac{H_i^d(t)}{\sum P(a,t)}.$$

The proportion of mortality in the counterfactual scenario and that in the vaccination scenario for cohort c are

$$M^0{}_i(c) = \frac{H_i^0(c)}{P_{lb}(c)} \text{ and } M^d{}_i(c) = \frac{H_i^d(c)}{P_{lb}(c)}.$$

Similarly, the proportion of morbidity (DALYs) in the counterfactual scenario and in the observed and projected vaccination scenario in year t are

$$M^0{}_i(t) = \frac{H_i^0(t)}{\sum P(a,t)} \text{ and } M^d{}_i(t) = \frac{H_i^d(t)}{\sum P(a,t) L_y(a,t)}.$$

The proportion of morbidity (DALYs) in the counterfactual scenario and in the observed and projected vaccination scenario for cohort c are

$$M^0{}_i(c) = \frac{H_i^0(c)}{P_{lb}(c)L_e(c)} \text{ and } M^d{}_i(c) = \frac{H_i^d(c)}{P_{lb}(c)L_e(c)}.$$

Let $M^0{}_i$ and $M^d{}_i$ be the proportion of disease burden in the counterfactual scenario and with-vaccination scenario from disease i, respectively. Denote the denominator in the above formulas (for $M^0{}_i(k)$ and $M^d{}_i(k), k = t, c$) by $D, i.e. \sum P\,(a,t), \sum P\,(a,t)L_y(a,t)$, or $P_{lb}(c)L_e(c)$. The proportion of mortality or morbidity can then be rewritten as

$$M^0{}_i = \frac{H_i^0}{D} \text{ and } M^d{}_i = \frac{H_i^d}{D}, \text{ respectively.}$$

Recalling the definition of absolute vaccine coverage v_i, one has

$$M^d{}_i = (1 - v_i)M^0{}_i + v_i M^v{}_i,$$

where $M^v{}_i$ is the proportion of disease burden among population vaccinated against pathogen i. Therefore, the mortality among the vaccinated individuals (M^v) is then obtained by

$$M^v{}_i = M^0{}_i - \frac{M^0{}_i - M^d{}_i}{v_i}$$

If $M^v{}_i < 0$, due to some herd effects, we adjust the proportion of disease burden in the counterfactual scenario by

$$M^0{}_i = \frac{M^d{}_i}{1 - v_i}$$

so that the proportion of disease burden among the vaccinated is zero ($M^v{}_i = 0$).

Disease burden accounting for population/coverage clustering and double counting

Assume that a population is vaccinated with N vaccines, and the population is clustered by the absolute vaccine coverage with the approach introduced in this context. Denote S_k as the proportion of disease burden among people who have received k vaccinesS_k. Then we have

$$S_0 = 1 - \prod_{j=1}^{N}\left(1 - M^0{}_j\right) \text{ if } k = 0, \text{ and}$$

$$S_k = 1 - \prod_{j=1}^{k}\left(1 - M^v{}_j\right)\prod_{i=k+1}^{N}(1 - M^0{}_i) \text{ if } k \neq 0.$$

The adjusted disease burden in the observed and projected vaccination scenario for people who have received j vaccines is given by

$$\hat{H}^d{}_j = \rho_j S_j D,$$

and the adjusted disease burden in the counterfactual scenario across all N diseases is estimated as

$$\hat{H}^0 = S_0 D.$$

Vaccine impact accounting for population/coverage clustering and double counting

Vaccine impact is obtained by calculating the difference in disease burden between the counterfactual and the observed and projected vaccination scenarios. Without consideration of vaccine clustering and double counting of disease burden averted, the aggregated vaccine impact across diseases is defined by

$$A = \sum_{j=1}^{n} \left(H_j^0 - H^d{}_j \right).$$

In contrast, when accounting for double counting and clustering, the aggregated vaccine impact across diseases is now defined by

$$B = \hat{H}^0 - \sum_{j=1}^{N} \hat{H}_j^d.$$

We therefore use the formula for B above to calculate the aggregated vaccine impact across all disease throughout the manuscript.

Uncertainty in total vaccine impact across diseases

This study involves a total number of 20 models in 10 different diseases. Each model produces 200 sets of pathogen-specific burden estimates, which are obtained by sampling over model parameter uncertainty and running the model for each sampled parameter set. Each model produced 200 sets of estimates to account for uncertainty inherent in model estimation and predictions. In addition, the number of sets per model were chosen to balance the need to quantify variation and computational feasibility in analysing such a quantity of projections. To measure the uncertainty in the aggregated vaccine impact across the 10 pathogens, we therefore make use of a bootstrap approach under the simplifying assumption that the drivers of uncertainty in each model are independent of those in any other model. In each bootstrap sample, we randomly select ten models (one model per disease) and for each model we randomly draw a set of estimates from their 200 model runs and calculate the statistic of interest. This procedure was repeated 100,000 times. The method accounting for population clusters and double counting of disease burden, was implemented on each of the 100,000 samples. Means, 2·5 and 97·5% quantiles from the 100 000 generated samples were calculated to get the central estimates and 95% credible intervals.

Proportion of unvaccinated children

Diseases such as measles with several vaccine doses are offered through routine first dose (e.g. measles-containing vaccine (MCV1), routine second dose (MCV2) and campaigns. ‘Within vaccine correlation’ refers to whether these doses for measles are independent or not. ‘Between vaccine correlation’ refers to the assumptions for vaccine doses against different pathogens. We explored how the proportions of unvaccinated children change for different correlation assumptions within and between vaccine doses. The methods of obtaining vaccine coverage in a cohort – for a) a particular vaccine with multiple doses and b) for multiple pathogens for multiple doses – by assuming 0% correlation and 100% correlation have been explained above by using equations (1)

and (2) respectively. The proportion of unvaccinated children in a particular cohort was obtained by using equation (2) but for $i = 0$.

Similar approaches were used for the cases of 0% correlation (as in equation (1)) for both within and between vaccines and for 100% (equation (2)) within and between vaccines.

Figures S5(a)-(c) show proportions of the 2019 birth cohort that have not been vaccinated in each country assuming different combinations of between and within vaccine correlations. Assuming 0% correlation for both within and between vaccine doses, the proportion of unvaccinated children is almost zero in all the countries (Figure S5(a)). The results in this paper are based on the assumption of 0% correlation for within vaccine and 100% correlation for between vaccines doses shown in Figure S5(b). The proportion of unvaccinated children for this scenario is presented in Figure S5(b).

Figures

Figure S5. Proportion of unvaccinated children for the 2019 birth cohort in each country, assuming doses for a vaccine against a particular pathogen and doses for vaccines against different pathogens are (A) both 0% (not) correlated; (B) 0% and 100% correlated, respectively; (C) both 100% correlated.

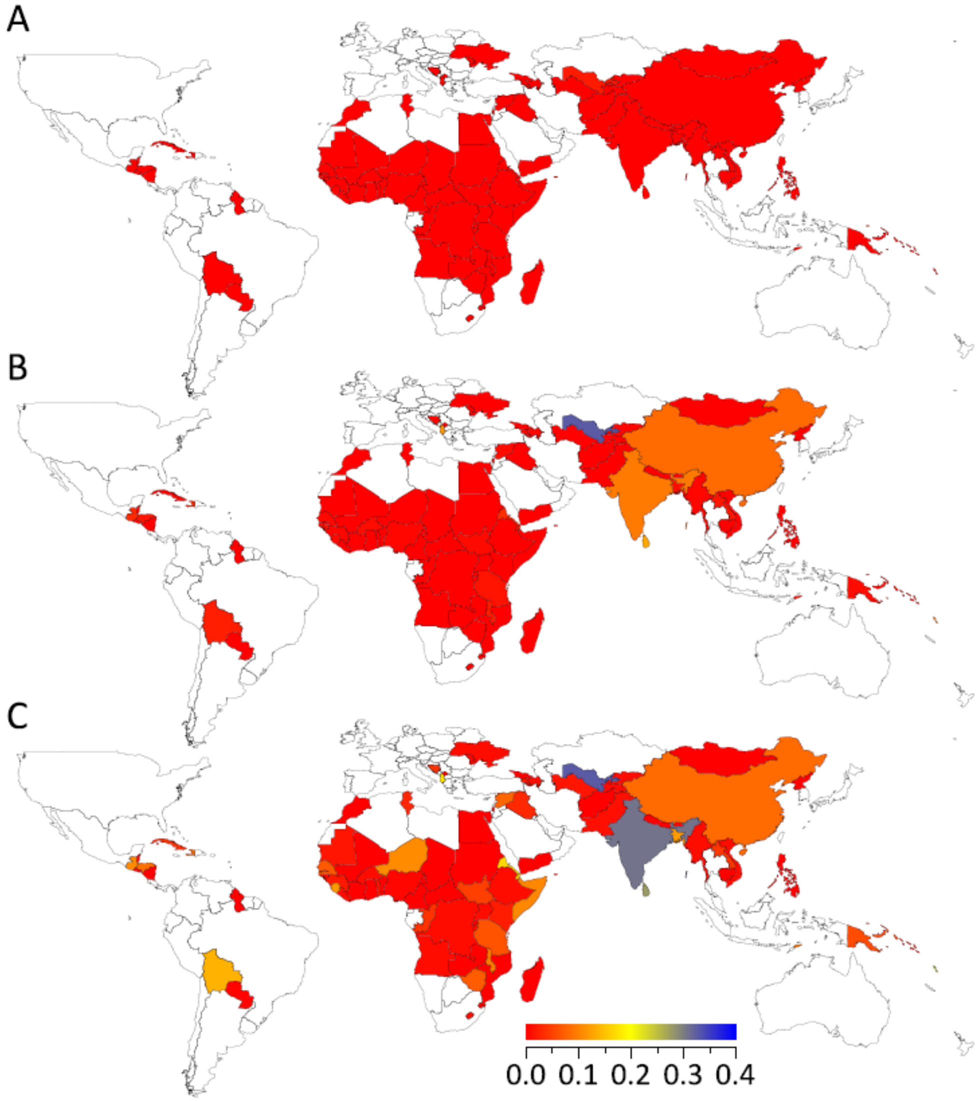

Measles burden estimates comparison among VIMC, IHME and WHO.

We compare burden estimates for measles generated from 98 countries in this analysis (VIMC), with those from Institute for Health Metrics and Evaluation (IHME) and World Health Organisation (WHO).

IHME measles burden estimates for all ages and under5s were obtained from the Global Burden Of Disease 2017 (GBD 2017) database[1]. WHO measles burden estimates for under5s were obtained from the Disease burden and mortality estimates (CHILD CAUSES OF DEATH, 2000–2017)[2] Burden estimates for all ages from WHO are not presented here since the total number of deaths are only available for the years 2000, 2010, 2015 and 2016.

We carried out two main comparisons:

- **Global:** We compare the total number of deaths attributed to measles for 91 low and middle-income countries shared in VIMC, IHME, and WHO for the years 2000-2017.
- **PINE countries:** We compare the total number of deaths attributed to measles per year from 2000-2017 in the PINE countries (i.e., Pakistan, India, Nigeria and Ethiopia) for the three organisations.

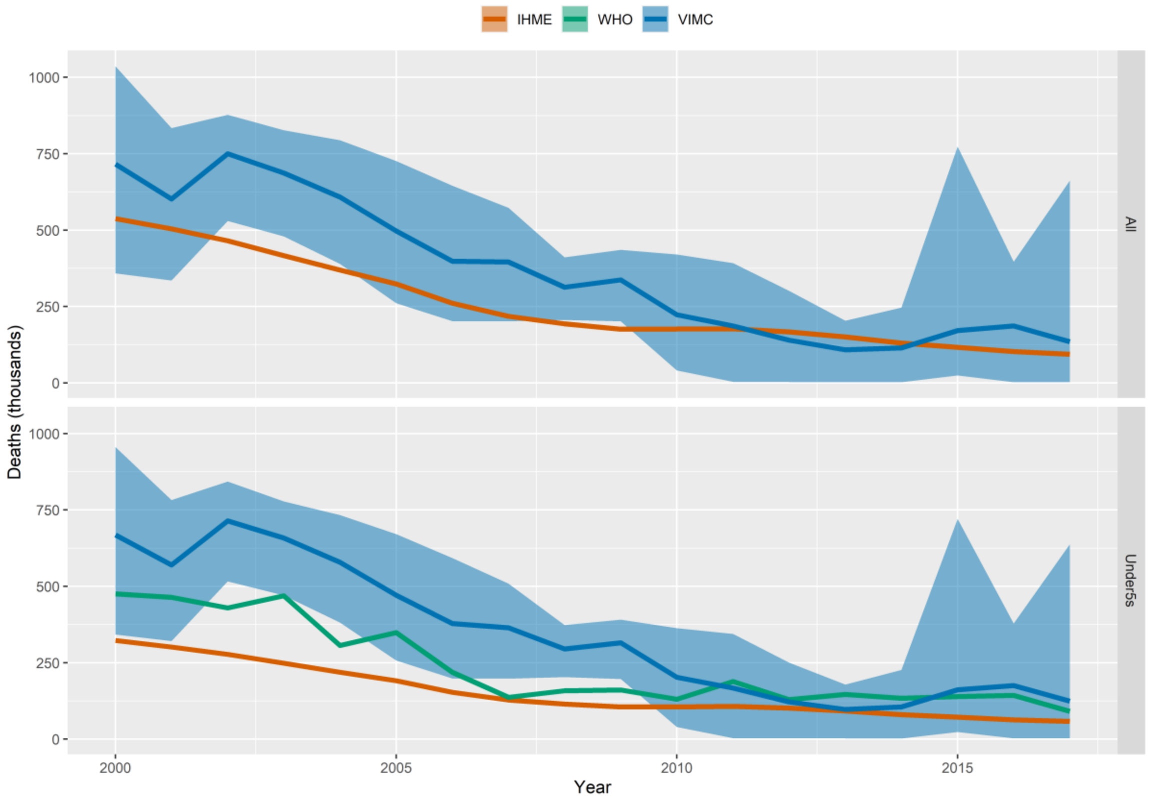

Figure i: Comparison of the total number of deaths attributed to measles among VIMC, IHME and WHO across 91 low and middle-income countries for both all ages and under5s for the years 2000-2017.

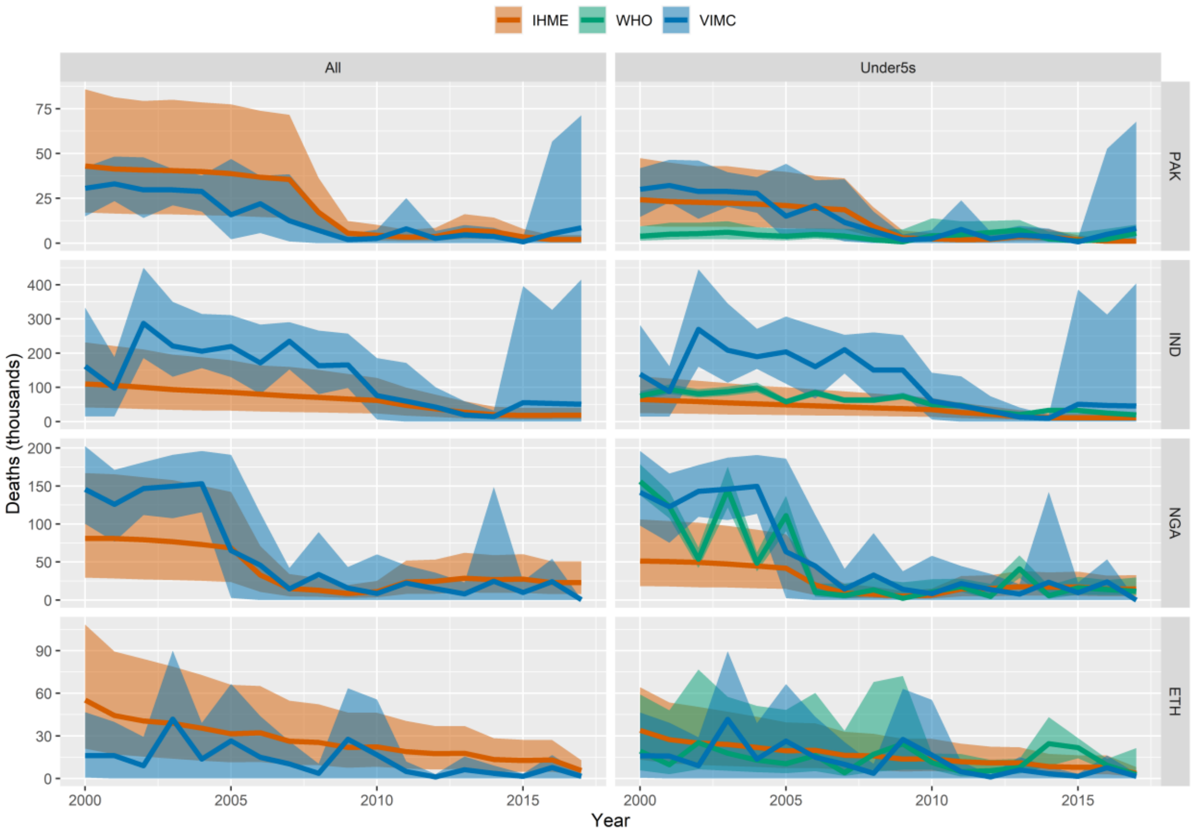

Figure ii: Comparison of the total number of deaths attributed to measles among VIMC, IHME and WHO across the PINE countries for both all ages and under5s for the years 2000-2017

Table ii: Total number of deaths (in thousands) from VIMC, IHME and WHO across PINE and all 91 countries (Global) for the periods 2000-2010 and 2011-2017. The ranges associated with VIMC estimates show 95% credible intervals (2.5 and 97.5 quantiles

Location	Period	All ages		Under5s		
		VIMC	IHME	VIMC	IHME	WHO
Global	2000-2010	5500 (3200-7100)	3600	5200 (3100-6600)	2200	3300
Global	2011-2017	1000 (43-2500)	940	950 (42-2300)	570	970
ETH	2000-2010	200 (0.65-440)	370	200 (0.53-430)	230	170
ETH	2011-2017	26 (0-60)	99	26 (0-59)	62	76
IND	2000-2010	2000 (1500-2500)	940	1800 (1400-2500)	540	830
IND	2011-2017	300 (0.01-890)	190	240 (0.01-870)	110	210
NGA	2000-2010	900 (550-1200)	540	880 (540-1200)	330	680
NGA	2011-2017	100 (0-210)	180	100 (0-190)	110	110
PAK	2000-2010	210 (130-290)	340	210 (130-280)	190	44
PAK	2011-2017	34 (0-99)	28	32 (0-94)	15	29

References

1. IHME. Global Health Data Exchange. http://ghdx.healthdata.org/countries (accessed 21 May 2020).

2. WHO. Disease burden and mortality estimates - Child causes of death, 2000–2017. https://www.who.int/healthinfo/global_burden_disease/estimates/en/index2.html (accessed 21 May 2020).

Model review process

All VIMC models were reviewed against pre-defined model standards in early 2018. Three pre-defined minimum standards and seven desirable standards set out the criteria for models' inclusion in VIMC.

First, models were required to generate required outputs (deaths, cases and DALYs) for all countries of interest (up to 98 countries), by year of current age and year of chronological time, and for different vaccination coverage scenarios. Second, models were required to use standardised demographic data provided by VIMC. Third, models needed to have comprehensive documentation, including a full model description to enable replication of the results, details of how the model represents key aspects of the natural history and epidemiology of the disease, and details of model parameterisation or fitting.

The seven desirable criteria were: 1) rigorous fitting to epidemiological data (with a preference for likelihood-based methods); 2) appropriate model complexity for the data available; 3) suitable data used for model fitting (ideally using data from the 98 countries of interest, a full range of data types, and data on vaccine efficacy/effectiveness; 4) out-of-sample validation; 5) ability to capture quantifiable uncertainty; 6) representation of indirect effects of vaccination (herd immunity) where epidemiologically relevant; 7) shared model source code.

The 2018 reviews were led by the VIMC management group. These reviews have been repeated annually against the same standards, but with a move towards light-touch peer reviews.

Detailed model descriptions

Hepatitis B - Center for Disease Analysis

PRoGReSs is a compartmental, deterministic, dynamic disease progression model of Hepatitis B virus (HBV) infection. It models the HBV-infected population in a country or region from infection (vertically or horizontally acquired) to progression of liver disease and eventually death. Population susceptible to HBV infection excludes anyone with a history of at least three doses of HBV vaccine or a history of previous exposure to HBV.

Country- or region-specific inputs of the model are divided into two major groups: demographic and epidemiological. Demographic inputs include population, background mortality, births, and male-to-female sex ratios at birth. Epidemiological inputs include the prevalence of Hepatitis B e antigen (HBeAg) among Hepatitis B surface antigen (HBsAg)-positive women of childbearing age, and intervention coverage (diagnosis and antiviral treatment of HBV infection in the general population, peripartum antiviral treatment of mothers, vaccination of infants (timely birth dose and ≥3 doses), catch-up vaccination, and liver transplantation).

Country-specific data for all epidemiological inputs are utilized when available which are described in the Polaris Observatory dataset [1]. When country-specific data is not available, regional averages are created from the aforementioned data and applied by GBD (Global Burden of Disease) regions, IMHE GBD Codebook 2017 [2].

Other epidemiological inputs are assumed to be constant across all countries or regions: progression rates of liver disease (specified by stage, serologic status, sex, and age group); mother-to-child transmission rates of HBV (specified by the serologic status of mother and the vaccination status of infant); proportions of HBeAg-negative and HBeAg-positive cases with a high viral load; and risk for developing a chronic HBV infection. The values and sources of inputs and assumptions of the model are described in past publications [1].

The secant method was used to calculate the sex- and age group-specific distribution of quinquennial incident cases of horizontally acquired HBV infection to fit modelled sex- and age group-specific HBsAg prevalence to reported prevalence in a given year.

The primary outputs of the model (stratified by gender and age) are the annual prevalence of HBsAg and HBV-related deaths by stage of liver disease, and serologic status (low-viral load, high-viral load, and on-treatment). The model has been validated by calibrating the model to historical empirical prevalence data, taking into account historical vaccination coverage, and then comparing the modelled outputs to more recent empirical prevalence data [1]. This validation focused on the HBsAg prevalence among those that would have been impacted by vaccination, but also examined HBsAg prevalence by other age and sex cohorts.

The total disease burden of HBV infection is generated by aggregating the low-level outputs described above. The key source of uncertainty in the model result from uncertainties in the reported prevalence of HBsAg (as described in the calibration procedure above). For the other sources of uncertainty, their importance depends on the output being examined. For example, if the uncertainty around prevalence among individuals aged 5 years old is being examined then the primary driver of uncertainty is the reported prevalence, followed by the mother to child transmission probability for the predominant form of prophylaxis. For liver-related deaths it is the

uncertainty around prevalence followed by the uncertainty surrounding disease progression and liver related deaths.

We calculated uncertainty intervals (UIs) and did sensitivity analyses using Crystal Ball release 11.1.2.3.500. β-PERT distributions were used for all uncertain inputs. Monte Carlo simulation was used to estimate 95% UIs, with 200 simulations run per country per scenario. UIs for prevalence estimates in all countries were assumed to be independent. The UIs for each country were calculated on the basis of published range inputs for prevalence, transmission rates, transition rates, and mortality rates.

Hepatitis B – Imperial College London

This population-level, deterministic, dynamic transmission model stratified the population by age and gender [3]. The model contains both acute (Severe Acute and Non-severe Acute) and chronic (Immune Tolerant, Immune Reactive, Asymptomatic Carrier, Chronic Hepatitis B, Compensated Cirrhosis, Decompensated Cirrhosis and Liver Cancer) mutually exclusive disease states. Of the chronic states, the Immune Tolerant and the Immune Reactive states are assumed to contain HBsAg+ HBeAg+ individuals, which are assumed to be 15 times more infectious than the HBsAg+ HBeAg- individuals in the other chronic and acute states [4-6]. The model also contains state variables for susceptible, recovered/vaccinated individuals. HBV-related deaths can occur from the Severe Acute, Compensated Cirrhosis, Decompensated Cirrhosis and Liver Cancer states.

Infection is spread in the population by both vertical and horizontal transmission, the rates of which are informed through fitting. The risk of acute infection becoming chronic is highest in the younger age groups and controlled by an exponential function that ranges in value from 88·5% for infants that contract HBV at birth from their infected mother to less than 5% risk for acute sufferers that are over 30 year of age [7]. Background mortality and migration are applied equally to all states. Younger age groups are assumed to undergo seroconversion from being HBsAg+ HBeAg+ to HBsAg+ HBeAg- at a faster rate than older age groups. In contrast, older age groups are considered at greater risk of developing liver cancer than are younger age groups. Transition rates and other model parameters such as vaccine efficacies (see next paragraph) are hard-coded into the model, and the number of infected cases and deaths is therefore driven by HBsAg and HBeAg prevalence in the population, which are controlled by model parameters that are calibrated separately for each country (see following paragraph). Since new HBV cases predominantly occur among the younger age groups (infants and 1 to 5 year olds), population make-up and fertility rates, which are dictated by the demographic data, heavily influence the rate of spread of the disease in the population.

The model takes into account the effects of the birth-dose (BD) and the infant vaccines on populations. The BD vaccine reduces the chances of an infant born to an infected mother contracting HBV at birth. The BD vaccine is assumed to be 95% effective in infants of mothers that are HBsAg+ HBeAg- and 83% effective in infants of mothers that are HBsAg+ HBeAg+. The infant vaccine is assumed to be 95% effective in conferring life-long protection to vaccinated individuals. Individuals are assumed to be either unvaccinated or have been given all infant vaccine doses necessary in their first six months of life to confer the full protection specified in the model. Herd immunity, determined by the proportion of immune individuals in the population, also provides some protection to unvaccinated individuals against horizontal transmission.

The model is calibrated to country-level, age-specific HBsAg+ prevalence data and HBeAg+/HBsAg+ prevalence data in pregnant women, which is obtained from the Polaris Observatory in

Boulder, Colorado, USA and other sources [8,9]. The model parameters that are calibrated include the risk of horizontal transmission to susceptible one to five year-olds and the risk of vertical transmission from HBsAg+ HBeAg- mothers to their infants at birth. Hence, the model calibrations determine the relative strengths of horizontal transmission to young children and vertical transmission within each country. Calibration is performed by the Nelder-Mead simplex algorithm [10]. Uncertainty in the impact of the model was represented by re-running the model with independently re-sampled parameter values for: (i) the underlying transmission rates between children under five years and the probability of transmission from mother to child (each value induced in the fitting is multiplied by a factor that is sampled randomly in the uniform distribution between 0.8 and 1.2), (ii) the efficacy of the vaccine in reducing the acquisition of infection (sampling randomly in the uniform distribution between 95% and 100%), and (iii) the efficacy of the birth dose in reducing acquisition of infection for those born to HbeAg+, HbsAg+ mothers (sampling randomly in the uniform distribution between 70% and 90%).

Other data sources include demographic data (female and male population sizes of one-year age group, total immigration, female fertility rates of five-year age groups, sex ratio of infants, female and male life expectancy of five-year age groups, female and male mortality rates of five-year age groups) from the United Nations World Population Prospects (UNWPP) 2017 Revision and infant and BD vaccine coverage data from the WHO/UNICEF Estimates of National Immunization Coverage (WUENIC) and Gavi, the Vaccine Alliance. These data are provided in the form of annual estimates on Montagu (VIMC's digital delivery platform).

Uncertainty in model estimates are due to uncertainties in the prevalence data that are used in the model calibration, as well as uncertainties in demographic data, historical coverage data and vaccine efficacies. The model structure is regularly updated to reflect the latest understanding of the natural history of HBV.

Hepatitis B – Goldstein

The model was developed by Susan Goldstein, Fangjun Zhou, Stephen Hadler, Beth Bell, Eric Mast and Harold Margolis at the US Centers for Disease Control and Prevention (CDC) [11]. It is a static deterministic model that estimates the global burden of hepatitis B and the impact of hepatitis B immunization programs. The model examines the mortality outcomes due to hepatitis B virus (HBV) infection, including deaths of fulminant hepatitis, and deaths of liver cirrhosis and hepatocellular carcinoma as results of chronic hepatitis B.

The model assumes infections occur in three age periods with different probabilities of developing symptomatic infections and progressing to chronic hepatitis B, which are: perinatal period, early childhood period (under 5 years), and the period over 5 years of age.

The rate of perinatal infection was determined by the prevalence of hepatitis B surface antigen (HBsAg) and hepatitis B e antigen (HBeAg) among pregnant women. Infants born to HBsAg positive and HBeAg positive mothers had a 90% chance of perinatal infection, while infants born to HBsAg positive and HBeAg negative mothers had a 10% chance of perinatal infection. The rate of infection in early childhood was determined by the prevalence of antibody to hepatitis B core antigen (anti-HBc) at age 5 after excluding perinatal infections, and the rate of infections between age 5 and 30 was determined by anti-HBc prevalence at 5 and 30 years of age. The prevalence at 30 years of age was assumed to have reached its peak in lifetime. A literature review was conducted on the prevalence of the hepatitis B seromarkers worldwide, and countries were grouped into 15 strata

with stratum-specific prevalence based on the reported prevalence in literature and the geographic proximity of the countries.

The model assumes 99% of the infants infected perinatally were asymptomatic during the acute infection phase, and 90% progressed to chronic hepatitis B, regardless of whether they were symptomatic or not. The model assumed 90% of children infected horizontally before the age of 5 had asymptomatic infection and 70% progressed to chronic hepatitis B. After the age of five, the chance of progressing to chronic hepatitis B was much lower: 70% of infections that occurred after the age of 5 were asymptomatic and only 6% progressed to chronic hepatitis B. Of the acute symptomatic infections, the risks of developing fulminant hepatitis B were 0·1% for perinatal infections, and 0·6% for horizontal infections. The case-fatality rate of fulminant hepatitis was 70% for all ages. Starting from 20 years of age, a small percentage of chronically infected persons (0·5% annually) seroconverted from HBsAg positive to negative, and were no longer at risk of complications related to chronic hepatitis B.

Liver cirrhosis and hepatocellular carcinoma account for the majority of hepatitis B deaths worldwide. The age-specific liver cirrhosis mortality rates were derived from mortality statistics from the United States and Taiwan (China). The age-specific hepatocellular carcinoma incidence was derived by fitting a polynomial function to data from populations with high HBV prevalence, including Alaska Natives, China, the Gambia and Taiwan (China). Given the low survival rates of hepatocellular carcinoma, the death rate of hepatocellular carcinoma was assumed to be the same as the incidence. The rates were adjusted by the prevalence of HBeAg in each country: populations who were HBeAg positive had 6 times higher the risk of developing hepatocellular carcinoma. The background all-cause mortality rates were from the life table published in the United Nations World Population Prospects.

The lives saved by hepatitis B vaccine were calculated as the difference between predicted deaths of hepatitis B in an unvaccinated cohort and a vaccinated cohort born in a certain year in one country. The vaccination coverages, namely the coverage of the timely birth dose (HepB birth dose within 24 hours of birth) and the coverage of the complete series of at least three doses of hepatitis B vaccine (HepB3) were from the WHO-UNICEF Estimates of National Immunization Coverage of the past years, and the coverage projection provided by the VIMC secretariat. 95% of infants who received the timely birth dose were assumed to be protected from perinatal infection, and 95% of infants who received the complete series of hepatitis B vaccine (indicated by HepB3 coverage) were assumed to be protected from horizontal infection in their lifetime. The model does not include herd immunity, or the effect of partial vaccination series.

The model used a sensitivity-to-parameters test, rather than a true uncertainty test. It was run with a spread of six parameters that are normally distributed around the original values, with a range of +/- 5%. Two vaccine efficacy parameters (for HepB3 and birth dose respectively) were originally set to 0.95 and then varied together (with the same value for both) between 0.9 and 1.0, normally distributed. These were not country-specific. Four prevalence parameters (HBsAg prevalence, HBeAg prevalence, anti-HBc prevalence at age 5, anti-HBc prevalence at age 30) all had country group-specific central values, and were varied in unison by +/- 5% around their central values.

Hib, PCV and Rotavirus – Johns Hopkins University

The Lives Saved Tool (LiST) is a deterministic linear mathematical model for estimating the health impact of changes in health intervention coverage in low- and middle-income countries (LMICs)

described in Walker, Tam, and Friberg [12]. LiST is a publicly available module within the Spectrum suite, a policy modeling system comprised of several software components. LiST contains over 80 health interventions, including vaccines, and has been used for over a decade to assist in public health decision-making and program evaluation. Evidence-based interventions included in the model have been demonstrated to reduce stillbirths, neonatal deaths, deaths among children aged 1-59 months, maternal mortality or risk factors.

The model describes fixed relationships between inputs (intervention coverage) and outputs (cause-specific mortality or risk factor prevalence) specified in terms of the effectiveness of the intervention for reducing the probability of that outcome under the assumptions that 1) country-specific mortality rates and cause of death structure will not change dynamically, 2) changes in mortality occur in response to changes in intervention coverage, and 3) distal factors, such as improvements in wealth, affect mortality by increasing intervention coverage or reducing risk factors.

The model is built on an underlying demographic projection derived from the United Nations Population Division (UNPD) and age structure for children (0-1, 1-5, 6-11, 12-23, 24-59 months) which serves as a theoretical cohort. Each model uses country-specific inputs of demographic growth [13], under-five mortality rates [14], and cause of death structure [15,16]. Together, these values are used to calculate cause-specific mortality and the potential deaths averted by increasing coverage of interventions. LiST attributes lives saved to changes in coverage of specific interventions, attributing impact first to preventative and then curative interventions, ordered sequentially from periconception, through pregnancy, delivery, followed by the specific age group. By using cause-specific efficacy and applying each intervention to the residual deaths remaining after the previous intervention, *LiST* ensures that double counting is avoided, and the potential impact of multiple interventions is not erroneously inflated.

Estimates of intervention efficacy are derived from existing reviews, many of which were published in five journal supplements [17-21]. National and subnational level impact estimates modelled by LiST have been validated against measured mortality reduction in various LMIC settings and for various packages of interventions [8,22-27].

LiST is used to generate estimates of cases and deaths averted for 0, 1, 2, 3 and 4 years of age due to coverage scale-up of pneumococcal conjugate vaccines (PCV), *Hemophilus influenza* type b (Hib) vaccine, and rotavirus vaccines. Deaths and cases are calculated separately in LiST. Incidence of diseases (number of cases per child per year) is used instead of cause-specific mortality as a baseline input to calculate cases. Cases and deaths averted by vaccination were calculated by applying estimates of scale-up in coverage in each of the countries. The model accounts for impact of other interventions in each country that could lower the risk of pneumonia, meningitis, or diarrhea incidence (e.g. clean water and sanitation), or reduce mortality from pneumonia, meningitis, or diarrhea (e.g. antibiotic treatment) using country-specific coverage of interventions drawing primarily on data from the Demographic and Health Survey (dhsprogram.com) and/or Multiple Indicator Cluster Survey (mics.unicef.org). The specific associations between interventions, risk factors, and mortality within LiST can be accessed via an interactive tool (LiSTVisualizer.org). Deaths and cases averted were calculated holding coverage of all other interventions constant.

The LiST uncertainty bounds are produced using a Monte Carlo approach. For each of the key assumptions in the model we have developed distributions around those values. These include efficacy of interventions, mortality rates, causes of death, relative risks of risk factor for mortality and incidence for severe pneumonia, meningitis and diarrhea. In general, beta distributions were used for effectiveness of interventions, correlated normal distribution for mortality rates, Dirichlet

distribution for death causes, and log-normal distribution for relative risks. Further information regarding rationale for sampling distributions can be provided upon request.

For the estimates presented here we were told to only vary efficacy of interventions in our uncertainty analysis. So, for each scenario we did 1,000 runs where efficacy values of all interventions were varied for each run based on a random draw from the distribution around the efficacy value of that intervention. The distribution of model outputs from the 1,000 runs were then used to produce the uncertainty bounds, which here were set to capture 95% of the distribution of results.

PCV-specific assumptions

LiST generates estimates of pneumococcal pneumonia and meningitis cases and deaths averted and non-pneumonia non-meningitis deaths averted by the coverage scale-up of PCV. The potential envelope of deaths averted by PCV was derived by applying a proxy for the proportion of pneumonia (proportion of *S. pneumoniae* among chest x-ray positive episodes of pneumonia: 32·96%) and meningitis (proportion of *S. pneumoniae* among severe bacterial meningitis cases: African meningitis A belt 46%; other 52%) deaths due *to S. pneumoniae* in the pre-vaccine era to the country-specific estimates of pneumonia and meningitis mortality [28,29]. Country-specific incidence of severe pneumonia pre-vaccine introduction was derived from an analysis by Rudan and colleagues [28]. The country-specific incidence of bacterial meningitis was calculated using the proportions of bacterial meningitis deaths due to *S. pneumoniae* and Hib from Davis et al [29], the *S. pneumoniae* case-fatality rates from O'Brien et al [30], and *Hib* case-fatality rates from Watt et al [31], divided by the total population 1-59 months of age. The proportion of pneumonia and meningitis cases and deaths averted was calculated by applying the 3-dose coverage of PCV scaled by the 58% efficacy of PCV in preventing all serotypes of invasive pneumococcal disease [32] to the fraction of deaths due to *S. pneumoniae*. The model includes only the direct effect of complete three-dose vaccination coverage.

Hib vaccine-specific assumptions

LiST generates estimates of Hib pneumonia and meningitis cases and deaths averted by the coverage scale-up of Hib vaccine. The potential envelope of deaths averted by Hib vaccine was derived by applying proxy estimates of proportion of pneumonia (proportion of Hib among chest x-ray positive episodes of pneumonia: 21·6%) and meningitis (proportion of Hib among severe bacterial meningitis cases: African meningitis A belt 40%; other 46%) deaths due to Hib in the pre-vaccine era to the country-specific estimates of pneumonia and meningitis mortality [28,29]. The same country-specific estimates of the incidence of severe pneumonia and meningitis for the PCV impact analysis were used. The proportion of pneumonia and meningitis cases and deaths averted was calculated by applying the three-dose coverage of Hib scaled the 93% efficacy of Hib in preventing invasive Hib disease [33] to the fraction of deaths due to Hib. The model includes only the direct effect of complete three-dose vaccination coverage.

Rotavirus vaccine-specific assumptions

LiST generates estimates of rotavirus diarrhea cases and deaths averted by the coverage scale-up of rotavirus vaccine. The potential envelope of deaths averted by rotavirus vaccine was derived by applying region-specific estimates of the proportion of rotavirus among severe diarrhea cases and deaths in the pre-vaccine era to the country-specific estimates of diarrhea mortality [34]. Region-specific estimates of the incidence of severe diarrhea were derived from the same source. The proportion of diarrhea cases and deaths averted was calculated by applying the complete dose coverage of rotavirus vaccine scaled by the region-specific efficacy of rotavirus vaccine in reducing

severe rotavirus gastroenteritis [35] to the fraction of deaths due to rotavirus. The model includes only the direct effect of complete rotavirus vaccination coverage.

Hib, PCV and Rotavirus – London School of Hygiene and Tropical Medicine (LSHTM)

UNIVAC (universal vaccine decision support model) is an Excel-based static cohort model with a finely disaggregated age structure (weeks of age <5 years, single years of age 5-99 years). A detailed description of the model and the methods for estimating vaccine impact are available in Clark *et al.* [36,37]. As a decision-support model, the primary purpose of UNIVAC is to provide national Ministries of Health in low- and middle-income countries (LMICs) with a framework to estimate the potential impact and cost-effectiveness of alternative vaccine policy options (www.paho.org/provac-toolkit). In the context of the Vaccine Impact Modelling Consortium (VIMC), UNIVAC was used to generate transparent desk-based estimates of the impact (% reduction in cases, clinic visits, hospitalisations, lifelong sequelae, deaths and DALYs) of three vaccines (haemophilus influenza type b - Hib, pneumococcal and rotavirus) over the period 2000-2030 in 98 LMICs (UNIVAC version 1.2.70, January 2018).

Interpolated 1-year time and age estimates [13] were used to calculate the number of life-years between birth and age 5·0 years for each of the 31 births cohorts (2000-2030) in each of the 98 countries. Life-years <5 yrs were multiplied by rates of disease cases and deaths (per 100,000 aged <5 yrs) to estimate numbers of cases and deaths expected to occur without vaccination between birth and age 5·0 years. The rates of disease cases and deaths due to Hib and Pneumococcal were based on estimates generated by Wahl *et al* [38] for the year 2015. For Hib, these included estimates for non-severe Hib pneumonia, severe Hib pneumonia, Hib meningitis and Hib non-pneumonia/non-meningitis (NPNM) in children aged <5 years. For Pneumococcal, they included estimates for non-severe Pneumococcal pneumonia, severe Pneumococcal pneumonia, Pneumococcal meningitis and severe Pneumococcal non-pneumonia/non-meningitis (NPNM) in children aged <5 years. In addition, the model includes cases of Pneumococcal acute otitis media (AOM) based on estimates by the CDC [39] and Monasta *et al* [40]. A large proportion of the total Pneumococcal disease cases estimated by the model represent Pneumococcal AOM. For both Hib and Pneumococcal infections, the risk of meningitis sequelae was estimated from a systematic review and meta-analysis by Edmond *et al* [41]. For rotavirus, country-specific estimates of rotavirus deaths <5 years were based on three independent sources of international burden estimates, recently compared in Clark *et al* [42]. Estimates of rotavirus disease cases (non-severe and severe) were based on systematic reviews and meta-analyses by Bilcke *et al* [43] and Fischer Walker et al [34]. Granular rotavirus disease age distributions (by week of age <5 years) were based on a recent systematic review and statistical analyses by Hasso-Agopsowicz *et al* [44].

Historical time-series estimates of pneumonia and diarrhoea deaths have declined in the absence of vaccination [16]. To avoid over-stating the impact of vaccination, we assume the disease-specific mortality rate will decrease without vaccination at the same rate as the overall under-five mortality rate [13]. For consistency, we make the same assumption for Hib/Pneumococcal meningitis and NPNM. We do not assume any decline in the incidence of disease cases, so case fatality ratios (CFRs) decline in each successive year.

Life expectancy estimates by age and year [13] were used to calculate YLLs (years of life lost due to premature mortality) from the age/year of disease death. YLDs (years of life with disease) were

calculated by multiplying disability weights by the average duration of illness. DALYs (YLLs + YLDs) were attributed to the year of disease onset.

For all three vaccines, estimates of vaccination impact were restricted to children aged <5 years. The impact was calculated by multiplying the expected number of disease events (cases, clinic visits, hospitalisations, deaths) in each week of age <5 years, by the expected coverage of vaccination in each week of age (adjusted for realistic vaccine delays/timeliness) and the expected efficacy of vaccination in each week of age (adjusted for the waning vaccine protection). The model accounted for partial vaccination by calculating the incremental impact of each dose of vaccination in each week of age. Rotavirus was modelled as a two-dose vaccine co-administered with DTP1 and DTP2 without age restrictions. Hib and Pneumococcal vaccines were modelled as a three-dose vaccine co-administered with DTP1, 2 and 3. For each vaccine, coverage projections by country and year were provided by Gavi, the Vaccine Alliance, over the period 2000-2030, via VIMC's digital delivery platform. Estimates of the timeliness of vaccination (coverage by week of age) were based on the timeliness of DTP1, 2 and 3 reported in USAID Demographic and Health Surveys (DHS) (dhsprogram.com) and Multiple Indicator Cluster Surveys (MICS) (mics.unicef.org). Methods for estimating vaccine timeliness have been described previously in Clark and Sanderson [45]. For Hib vaccination, dose-specific efficacy was based on a global systematic review and meta-analysis of RCTs by Griffiths *et al* [33]. For rotavirus, vaccine efficacy by dose and duration of follow-up (year 1 and year 2) was based on a meta-analysis of RCTs by Patel et al [46]. For Pneumococcal, efficacy against all serotypes of pneumococcal disease (vaccine type and non-vaccine type) was based on a global meta-analysis by Lucero *et al* [47].

UNIVAC is not a transmission dynamic model, and thus excludes indirect effects (both positive and negative). This is likely to lead to substantial under-estimates of impact in some countries, particularly for Hib vaccine. More detailed validation against real-world post-introduction evidence of impact is needed. However, the available data in many of the countries included in this desk-based analysis are insufficient to allow validation of modelled estimates (against real-world estimates of post-introduction vaccine impact) and/or parameterisation of a country-specific transmission dynamic model. As such, there is a good deal of uncertainty in the predicted estimates for many countries.

For simplicity, the parameters were assumed to be independent and were sampled from PERT-Beta distributions [48]. For each parameter, the best available central input estimate represented the mean of the distribution and the low and high input estimates represented the range. Each vaccine scenario was run 200 times. The following parameters were varied in each probabilistic run: (a) population size by single year of age; (b) annual disease incidence rate aged <5 years; (c) annual disease mortality rate aged <5 years; and, (d) vaccine efficacy.

HPV – Harvard University

The Center for Health Decision Science companion model is a flexible tool that has been developed to reflect the main features of HPV vaccines, and to project the potential impact (health and economic consequences) of HPV vaccination at the population level in settings where data are very limited [49]. The model is constructed as a static cohort simulation model based on a structure similar to a simple decision tree and is programmed using Microsoft® Excel and Visual Basic for Applications (Microsoft Corporation, Redmond, WA). The model tracks a cohort of girls at a target age (e.g. 9

years) through their lifetimes, comparing health and cost outcomes with and without HPV vaccination programs.

Unlike more complex empirically-calibrated micro-simulation models [50-52], the companion model does not fully simulate the natural history of HPV infection and cervical carcinogenesis. Instead, based on simplifying assumptions (i.e. duration and stage distribution of, and mortality from, cervical cancer), which rely on insights from analyses performed with the micro-simulation model, and using the best available data on local age-specific incidence of cervical cancer and HPV 16,18 type distribution and assumed vaccine efficacy and coverage, the model estimates reduction in cervical cancer risk at different ages. By applying this reduction to country-specific, age-structured population projections incorporating background mortality [13], the model calculates averted cervical cancer cases and deaths, and transforms them into aggregated population health outcomes, years of life saved and disability-adjusted life years (DALYs) averted. DALYs are calculated using the approach adopted by the Global Burden of Disease (GBD) study [53].

The companion model captures the burden of HPV infection by estimating the number of cervical cancer cases caused by HPV infection based on epidemiological data obtained from various sources [49]. In the absence of vaccination, women may develop HPV infections and cervical cancer based on epidemiologic estimates specific to each country. The model assumes that age-specific cervical cancer incidence, average age of sexual debut, and the level of other risk factors remain constant over the time horizon of the model. It assumes that girls are fully immunized and that girls effectively immunized against HPV16/18 can be infected with non-16/18 type HPV (e.g., no cross-protection is assumed). Vaccine-induced immunity is assumed to be lifelong. All assumptions are varied in sensitivity analyses.

Three key parameters were identified for probabilistic sensitivity analysis (PSA): HPV-16/18 type distribution, vaccine efficacy, and age-specific cervical cancer incidence. Each parameter was assigned a beta distribution for probabilistic sampling, with the bounds determined by: (1) empirical data for type distribution; (2) an assumed 75% lower bound from the 100% base case/upper bound for vaccine efficacy; and (3) confidence intervals estimated from cervical cancer registry data from India for incidence. Of five identified focus countries – Democratic Republic of Congo, Ethiopia, India, Nigeria, and Pakistan – the parameter sets for the country identified with the widest uncertainty (i.e., Nigeria) were transformed into relative multipliers and extrapolated to the remaining countries in the analysis.

HPV – LSHTM

The Papillomavirus Rapid Interface for Modelling and Economics (PRIME) is a static, proportional impact model that can estimate the impact of HPV vaccination on cervical cancer cases and deaths, as well as the cost-effectiveness of vaccination programmes, in 179 countries [54].

The PRIME model was developed in collaboration with the World Health Organization (WHO), with inputs from investigators at Laval University and Johns Hopkins University as well as LSHTM. It is designed to estimate the impact and cost-effectiveness of HPV vaccination in low- and middle-income countries (LMICs). In addition to its application in the Vaccine Impact Modelling Consortium (VIMC) it is used to support vaccination planning at a country-level. It has been validated against 17 published studies using HPV vaccine economic models set in LMICs [54]. It was also endorsed by the WHO's expert advisory committee, the Immunization and Vaccines Implementation Research Advisory Committee (IVIR-AC) to provide a conservative estimate of the cost effectiveness of

vaccinating girls prior to sexual debut. The Excel-based version of the model and documentation are publicly accessible at http://primetool.org/ for use by country program managers and planners to facilitate country-specific decision-making in LMICs. The R package of the model (prime) is available upon request and has additional functionality such as multiple cohorts and probabilistic sensitivity analysis. It can be used for research, global analyses and to generate the vaccine impact estimates used by VIMC.

Data inputs include country and age-specific cervical cancer incidence, prevalence, and mortality among females. The model estimates vaccination impact in terms of reduction in age-dependent incidence of cervical cancer and mortality in direct proportion to vaccine efficacy against HPV 16/18, vaccine coverage and HPV type distribution. It assumes that vaccinating girls prior to infection with HPV types 16 and 18 fully protects them from developing cervical cancer caused by HPV 16 and 18, in accordance with vaccine trials [55]. The model assumes a two-dose schedule with perfect timeliness at the target ages given in the coverage estimates. Herd effects are not considered meaning that the vaccine impact estimates produced are conservative. The impact of vaccinating multiple age cohorts is estimated by using the most conservative assumption that 9-14 year old girls who have sexually debuted are not protected, although these assumptions do not change the overall impact estimates significantly [56].

Probabilistic sensitivity analysis was conducted by varying incidence and mortality estimates of cervical cancer from GLOBOCAN database of the International Agency for Research on Cancer. They were varied uniformly by ± 20% and ± 50% of the mean estimates for each country of high and low data quality respectively.

JE – University of Oxford

This deterministic dynamic model uses a basic catalytic model for the force of infection (FOI), in which individuals become infected and are then immune. Vaccination is modelled as a removal of susceptibles from the susceptible class. As humans are dead-end hosts for Japanese encephalitis (JE), infection comes from animal reservoirs via mosquitoes. This simple model successfully captures the natural history and transmission dynamics of JE.

A systematic review of all published studies and publicly available JE surveillance data was undertaken to collate a dataset of age-stratified case data. The FOI model is fit to age-stratified national surveillance data that were publicly available and data identified via a systematic review of age-stratified JE case data [57]. Data from a total of 10 countries and 17 studies was used, which gave estimates of a wide range of force of infection parameters, as expected for the wide geographical locations. The model is fit in a Bayesian framework using RStan. This gave distributions for the FOI estimates for all locations in which data is available. For areas in which data was not available we extrapolated from areas in which it was, using the WHO groupings of transmission intensity [59]. In order to generate uncertainty in the case burden estimates, all model parameters were sampled from the posterior distributions of the FOI estimates. In addition, the symptomatic rate was sampled from uniform distribution (0·002, 0·004) [60] and the proportion of these symptomatic cases that died was from uniform distribution (0·2, 0·3). There is limited information collated currently on these proportions so uniform distributions were used. On-going work is estimating the distributions of these currently. The distributions were assumed to be independent. More detail on model and model parameters is available [57]. The vaccine was assumed to be 100% effective, and protection lifelong.
Disease burden was generated from the 'bottom up': i.e. from infection rates applying parameters governing the proportion of infections that are symptomatic and the proportion that die (case

fatality ratio). The key uncertainties which affect disease burden estimates are the method of extrapolation of FOI from areas in which there is data to areas in which there is not. Spatial modelling work is on-going to improve this extrapolation and to make estimates on smaller spatial scales. The case fatality ratio is also uncertain, and further work undertaking a systematic review of this is ongoing.

JE – University of Notre Dame

A stochastic model of Japanese encephalitis virus (JEV) transmission with a constant force of infection (FOI) was used to estimate the burden of JE and the potential impact of vaccination in JE-endemic countries. JEV is a mosquito-transmitted, zoonotic pathogen that requires an animal host for ongoing transmission, given humans are believed to be a dead-end host [61]. Therefore, JE incidence is limited to geographic regions where there are suitable hosts and vectors to sustain both ongoing transmission in animal hosts and spill-over to humans. To estimate the number of JEV infections the model first estimates the number of people at risk of infection and then estimates the transmission intensity in each country. JE burden (including cases, deaths, and DALYs) was then estimated from the number of JEV infections.

To identify the areas suitable for sustained JEV transmission, and the size of the population living in at-risk areas, a spatial analysis of the risk factors associated with JEV was conducted. Potential JEV-endemic areas were identified using large-scale spatiotemporal datasets related to suitable climate conditions for the vector species, suitable habitat conditions for the vector, and the presence of potential zoonotic hosts. Transmission was assumed to occur only in areas occupied by the primary vector, *Culex tritaeniorhynchus* [62], or where the annual minimum temperature exceeded 20˚C and annual precipitation exceeded 150 cm. Suitable habitat conditions included areas with rice cultivation or nearby wetlands, excluding urban areas [63]. Within these suitable areas, people were considered at risk of infection if the density of domestic pigs or fowl exceeded 1 per km (with uncertainty represented by varying the animal threshold from 0-10 per km) [64]. Risk maps were validated using seroprevalence and surveillance data.

Next, the FOI in each country was estimated from age-specific incidence data using a catalytic model. FOI represents the per-capita rate at which susceptible individuals are infected. Age-specific incidence data was obtained from a literature search, restricted to studies conducted in areas with no history of vaccination (or prior to documented vaccination) to simplify the estimation process. For several countries where no age-specific incidence data was available, FOI estimates were drawn from the posterior estimate of a neighbouring country. FOI estimates for each study were estimated using a maximum likelihood approach, using the observed numbers of JE cases per age class in each year. Study-specific FOI values were estimated using a Bayesian framework via a Markov chain Monte Carlo (MCMC) approach implemented in the software package STAN. The FOI was given an uninformative truncated normal prior with a mean of zero and a standard deviation of ten. The probability of asymptomatic infection and the case fatality ratio for symptomatic infections (see below) were assumed to be independent of the FOI.

The annual number of JEV infections for a given study area were then calculated from the FOI estimate and the size of the at-risk population. In the absence of vaccination, the number of infections in age class was calculated by multiplying the age-specific probability of infection by the number of at-risk individuals in the age class.

Vaccination reduced the number of at-risk individuals in each targeted age class based on provided coverage estimates. The number of JE cases and deaths were then estimated from the number of JEV infections based on the proportion of infections that are symptomatic or fatal. The distribution of encephalitis cases *C* observed from *I* JEV infections was modelled assuming the asymptomatic to symptomatic (A:S) ratio is represented by a gamma distribution with shape parameter = 3·58 and a rate parameter = 0·011. This distribution produces an A:S ratio with a median of 295:1 (95% CI: 83:1 to 717:1), similar to the range of published estimates [61]. The case fatality ratio (CFR) from JE, *D*, was assumed to follow a Beta distribution with parameters α = 1·88, β = 3·50, resulting in a median symptomatic CFR of 0·33 (95% CI: 0·05-0·75), which reflects the large uncertainty in this parameter [61]. The annual burden of JE at the national-level was calculated using disability-adjusted life years (DALYs) with disability weights taken from the Global Burden of Disease 2016 report [65].

Measles – LSHTM

The Dynamic Measles Immunization Calculation Engine (DynaMICE) is a dynamic age-stratified population model of measles transmission dynamics to estimate the public health impact and cost-effectiveness of routine vaccination programmes and supplementary immunisation activities (SIAs) in low- and middle-income countries [66]. It was originally developed for work with the World Health Organization and received inputs from investigators at Harvard University and Montreal University as well as LSHTM. It was subsequently used to inform vaccine impact projections for Gavi, the Vaccine Alliance and the Bill & Melinda Gates Foundation. The model provides a flexible framework that can be adapted to different countries with the aim to study several vaccination scenarios based on available data sources. For example, the model has been adapted to characterise measles transmission and dynamics in India, based on measles data from the Million Death Study [67], as well as to quantify the impact of adding interventions to measles SIAs in India by interfacing with the Lives Saved Tool (LiST) [68].

As measles is a highly transmissible childhood infection, disease dynamics are inextricably linked to population structure and demographic parameters. To enable precision in the estimation of disease burden and the contact processes that drive transmission, the model is age-stratified to include weekly age groups from birth to 3 years of age, and yearly age-groups from 3-100 years of age. The underlying epidemic model is a compartmental SIR model, where individuals can either be susceptible (S) to measles, infected (I) or recovered (R) with life-long immunity. After a certain duration of maternal immunity, births replenish the pool of susceptibles that in the absence of vaccination fuel periodic outbreaks of measles driven by the magnitude of the birth rate and the strength of seasonality in transmission parameters. Susceptibles get infected through contact with infected individuals, with mixing determined by age-dependent contact patterns. The contact matrix used for this exercise is the POLYMOD contact matrix for Great Britain [69] as it was best able to reproduce transmission dynamics across a range of countries, but this can be updated to represent local population age structure. Following infection, individuals either recover and gain lifelong immunity, or die as described by country-specific age-dependent case fatality ratios (CFRs).

Routine vaccination is modelled through first- and second-dose measles-containing vaccine (MCV1 and MCV2) schedules (corrected for the right cohorts) with the additional option of including SIAs. Vaccines are assumed to be “all or nothing” with effectiveness equal to 84% for the first dose among children under the age of one year, 93% for the first dose among children over the age of one year, and 99% for both doses [70], with life-long vaccine-induced immunity.

A probabilistic sensitivity analysis was run by varying vaccine take and mortality parameters. There are three different vaccine take parameters in the model (first dose given at less than 12 months, first dose given at more than 12 months of age, and second dose), and each of those was allowed to vary by drawing parameters from a uniform distribution allowing for 5% difference in take. Mortality was varied by drawing parameters from a uniform distribution allowing for 25% difference in mortality (so, generating numbers between 0.75-1.25 which then multiplied the original CFR value).

Measles – Pennsylvania State University

The PSU measles model is a semi-mechanistic, age-structured, discrete time-step, annual SIR model. Unlike conventional SIR models, which describe dynamics at the scale of an infectious generation (TSIR REF) [71] or finer (basic REF) [72], it models the aggregate number of cases over one-year time steps. While this is coarse relative to the time scale of measles transmission, it matches the annual reporting of measles cases available for all countries since approximately 1980 for all countries through the WHO Joint Reporting Form (JRF). To account for the fine-scale dynamics that are being summed over a full year, the model describes the number of infections ($I_{i,t}$) in country *i* and year *t*, and age class *a* as an increasing function of the fraction, $p_{i,t}$, of the population susceptible in age class *a* at the start of year *t*, $S_{i,t}$:

$$E[I_{a,i,t}] = p_{i,t} * S_{a,i,t} ,$$

where $E[\cdot]$ indicates the expectation and $p_{i,t}$ is a country and year specific annualized attack rate modeled as:

$$p_{i,t} = invlogit(-\beta_{0,i} + \beta_{1,i} * \frac{\sum_a S_{i,t}}{N_{i,t}} + e_t) ,$$

where invlogit() indicates the inverse logit function, $N_{i,t}$ is the total population size in country *i* and year *t* over all age classes, and e_t is a Gaussian random variable with mean 0 and variance σ^2. The parameters $\beta_{0,i}$, $\beta_{1,i}$, and σ^2 are fit to each country independently using a state-space model fitted to observed annual cases reported through the JRF from 1980-2016 as described by Eilertson et al [73]. Historical population and vaccination coverage values are provided by WHO as described by Simons et al [74].

The number of susceptible individuals in each single-year age class *a* (*a*=2,…, 25) is equal to the number not infected in the previous year, nor immunized through supplemental immunization activities (SIAs). The number susceptible is further deprecated by the crude death rate. The efficacy of doses administered through SIAs is assumed to be 99%. The number of susceptible individuals in age class *a*=1 is assumed to be 50% of the annual live birth cohort; this assumes that all children have protective maternal immunity until 6 months of age. Age class *a*=2 and *a*=m is assumed to receive a first and second dose (respectively) of routine measles vaccination before the start of the time step; thus the number susceptible is further reduced by the product of the coverage and the efficacy. Efficacy is assumed to be 85% and 93% for the first dose in countries delivering at 9m and 12m of age, respectively, and assumed to be 99% for the second dose.

Deaths are calculated by applying an age- and country-specific case fatality ratio (CFR) to each country. CFRs for cases below 59 months of age for all countries were taken from Wolfson et al [75]; CFR for cases above 59 months of age are assumed to be 50% lower than those applying to under-5s.

Forward simulations of this model assume random variation in the annual attack rate according to the parameter σ^2. Further, each forward simulation draws $\beta_{0,i}$, $\beta_{1,i}$ at random from the joint 95% interval estimate of each parameter. Future vaccination coverage values, for routine and SIAs, are assumed known and future birth and death rates are assumed known.

MenA – University of Cambridge

This model is a compartmental transmission dynamic model of *Neisseria meningitidis* group A (NmA) carriage and disease to investigate the impact of immunisation with a group A meningococcal conjugate vaccine, known as MenAfriVac, as published in 2015 [76]. The model is age-structured (1 year age groups up to age 100) with continuous ageing between groups. Model parameters were based on the available literature and African data wherever possible, with the model calibrated on an ad-hoc basis as described below.

The population is divided into four states, which represent their status with respect to meningitis infection. Individuals may be susceptible, carriers, ill or recovered and in each of these states be vaccinated or unvaccinated, with vaccinated individuals having lower risks of infection (carriage acquisition) and disease (rate of invasion). We assume that both carriers and ill individuals are infectious and can transmit the bacteria to susceptible individuals. The model captures the key features of meningococcal epidemiology, including seasonality, which is implemented by forcing the transmission rate, the extent of which varies stochastically every year.

Since only a small proportion of infected individuals develop invasive disease, disease-induced deaths are not included in the model. From each compartment there is a natural death rate from all causes. Carriage prevalence and disease incidence varies with age, and the model parameterises these distributions using a dataset from Niger [77]; the case:carrier ratio consequently varies with age. The duration of 'natural immunity' is an important driver of disease dynamics in the absence of vaccination but good data on this parameter is lacking; instead prior estimates are used [78].

The model assumes that mass vaccination campaigns occur as discrete events whereas routine immunisation takes place continuously. After vaccination, individuals are assumed to be protected for an average of 10 years, consistent with experience with other meningococcal conjugate vaccines and observations of persisting protection to at least 5 years in trial participants [79]. Vaccine efficacy against carriage and disease is 90%.

Disease surveillance is not comprehensive across the meningitis belt, so disease burden is uncertain in several countries. The model therefore classifies the countries into three categories, based on the incidence levels using historical data. This classification defines the transmission dynamic parameters. The model generates estimates of case incidence, to which a 10% case-fatality ratio [80,81] is applied to estimate mortality. To estimate DALYs it is assumed that 7·2% of survivors have major disabling sequelae [41] with a disability weight of 0·26.

Countries were stratified into high and medium risk, and different infection risk applied based on this stratification. As there was insufficient information to define infection risk on a country-by-country basis, the approach/stratification was agreed with experts in the WHO meningitis team. For countries only partly within the meningitis belt, only the (subnational) area at risk was included. For example, in Ghana only the northern regions were included. Areas outside of the meningitis belt were not included.

To produce estimates on the impact of vaccination, 200 simulation runs were generated by stochastically varying the baseline transmission rate to reflect between-year climactic or other external variability. Although each individual simulation reflects the reality of irregular and periodic epidemics, as visually compared to time series from Chad and Burkina Faso and analysis of inter-epidemic periods, the resulting averaged estimates give a fairly stable expected burden of disease over time. Uncertainty in other model parameters is currently not quantified.

MenA – Kaiser Permanente Washington

This model is a stochastic, age-structured, compartmental model of the transmission of serogroup A *Neisseria meningitidis* (MenA) [82]. Model compartments track hosts' status with respect to MenA exposure (as Susceptible, Colonized, and Invasive Disease) and adaptive immunity to infection/disease (as High, Low, or No immunity). Exposure to MenA through colonization leads to the "low immunity" state, in which individuals are still susceptible to colonization but have a reduced risk of developing invasive disease if colonized. MenA colonization among individuals with low immunity leads to a "high immunity" state, which is highly protective against both colonization and disease.

Model parameters are defined, where possible, from published data [82]. Estimates of the age-specific force of infection were obtained by fitting the model to longitudinal studies of the prevalence of MenA colonization in Burkina Faso [83]; model fitting was through an iterative numeric algorithm. After estimating parameters for the age-specific force of infection, the age-specific per-carrier rate of progression to invasive disease was estimated by fitting the model to longitudinal data on the incidence of MenA disease in Burkina Faso [82] and the expected age distribution of MenA cases [77].

In the simulations, mortality burden estimates are obtained in a "bottom-up" manner, in that case fatality ratios (CFRs) [77,84-89] are applied to simulated case counts.

For estimating the impact of serogroup A polysaccharide conjugate vaccine (MenAfriVac), two versions of the model were developed – a "Base" model, in which vaccination was assumed to be equivalent to naturally-induced "high immunity", and a "Vaccination-Plus" model, in which vaccination is assumed to be superior to natural immunity, based on estimates of vaccine effectiveness and serum bactericidal antibody (SBA) concentrations [90]. Subsequent model validation has shown that the Vaccination-Plus model better captured the dynamics of NmA in Burkina Faso following mass vaccination campaigns with MenAfriVac, and only the Vaccination-Plus model is used in the present work [91].

Countries were stratified based on risk (hyper-endemic vs. not), and different forces of infection used based on risk group. For countries only partly within the meningitis belt, the model was restricted to the area at risk.

Variability in infections rates is represented by randomly sampling values for the force of infection parameters within ±20% of their estimated values; new values are sampled annually to reflect annual variation in climate or other external factors. Mean estimates of incidence are obtained from 100 iterations of the stochastic model using a different random seed for each run. Probabilistic sensitivity analysis (PSA) is used to characterise uncertainty in the age-specific CFR and vaccine effectiveness (VE) against colonization and disease. For this, 200 iterations of the model are run, with randomly sampled values for CFR and VE in each iteration. Sampling distributions for CFR and VE were defined based on estimated CFR and VE from previously published studies [82,91]. Values for other model parameters were held fixed at their estimated value from model fitting. Other key

sources of uncertainty not presently included in PSA are the expected duration of vaccine-induced protection and the force of infection in countries for which NmA surveillance data are lacking.

Rubella – Public Health England

This is an age and sex-structured, deterministic, compartmental model of the transmission dynamics of rubella [92-94]. The population is stratified into those with maternal immunity (lasting 6 months), susceptible, pre-infectious (infected but not yet infectious), infectious and immune, using annual age bands and a "Realistic Age Structure"[95]. Country-specific birth and age-specific death rates were fixed at 2010 levels and calculated from UN population survival data for 2010-15 [96] respectively. The supplement to reference [92] provides the model's differential equations.

The force of infection (rate at which susceptibles are infected) changes over time and is calculated using the number of infectious individuals and the effective contact rate (rate at which infectious and susceptible individuals come into effective contact). Contact is described using the following matrix of "Who Acquires Infection From Whom":

$$\begin{pmatrix} \beta_1 & 0.7\beta_2 \\ 0.7\beta_2 & \beta_2 \end{pmatrix}$$

The effective contact rate differs between <13 and ≥13 year olds, with its relative size based on contact survey data [97]. β_1 and β_2 are calculated from the average force of infection in <13 and ≥13 year olds, estimated from age-stratified rubella seroprevalence data, which had been collected before rubella containing vaccine (RCV) was introduced [93]. Seroprevalence data were available for 28 countries (see [94]). For countries lacking seroprevalence data, we used data from countries in the same WHO region [93,94]. Confidence intervals (CI) on the force of infection were calculated using 1000 bootstrap-derived-seroprevalence datasets [93,94].

Country-specific numbers of congenital rubella syndrome (CRS) cases in year *y* during 2001-2080 were calculated by summing the number of CRS cases born each day to women aged 15-49 years. As assumed elsewhere [92-94,98], infection during the first 16 weeks of pregnancy carries a 65% risk of the newborn having CRS. The number of CRS deaths in year *y* was calculated by multiplying the number of CRS cases born in year *y* by the assumed case fatality rate (30%). The number of DALYs for cases in year *y* was calculated by multiplying the number of CRS cases in year *y* by the corresponding DALY [99], which was based on the country-specific World Bank Income group for 2017 [100]. Both the DALYs and the assigned World Bank income group remained fixed over time.

Confidence intervals on the outputs for each setting were calculated as the 95% range of the outputs obtained by running the model using 200 combinations of 5 randomly-sampled parameters. The parameters were the pre-vaccination force of infection which was used to calculate the contact parameters (see above), the risk of a child being born with CRS if his/her mother had been infected during pregnancy, the CRS-related case-fatality rate, the vaccine coverage and the vaccine efficacy. The pre-vaccination force of infection was sampled from 1000 bootstrap-derived force of infection estimates, obtained by fitting catalytic models to bootstrap-derived seroprevalence data for that setting, or, if that setting lacked seroprevalence data, from bootstrap-derived force of infection estimates from countries in the same WHO region as the country of interest [93,94]. The remaining parameters were randomly sampled from distributions reflecting their plausible range, as implied by published studies, wherever possible[92]. The sampling was conducted assuming that the parameters were independent.

Rubella – Johns Hopkins University

This is a discrete-time stochastic age-structured compartmental rubella transmission model, building from previous work describing rubella dynamics [101,102]. The key feature of the model is a matrix that at every time-step defines transitions from every combination of epidemiological stage (maternally immune 'M', susceptible 'S', infected 'I', recovered 'R', and vaccinated 'V', taken to indicate the effectively vaccinated) and age group (1 month age groups up to 20 years old, then 1 year age groups up to 100 years old) to every other possible combination of epidemiological stage and age group. The discrete time-step was set to about two weeks (i.e., 24 time steps in a year), the approximate generation time of rubella.

People are born either directly into the 'susceptible' class or move there as the passively-acquired 'maternal immunity' wanes over the first year of life. As individuals age, they can be exposed to either vaccination, which if successful moves them permanently into the 'vaccinated' class, or to natural infection, moving them to 'infected' for a time-step (or rubella generation) then permanently into the 'recovered' class. In addition to these epidemiological transitions, there are demographic transitions including birth, death, and ageing.

Vaccination coverage was adjusted based on the assumptions that repeated vaccination activities are not completely independent and a portion of the population may always remain inaccessible to vaccination. The age- and time-specific proportion inaccessible were assumed to correspond those not covered by DTP vaccination, as reported by the WUENIC DTP routine vaccination rate estimates [103]. Duration of maternal immunity [104] and vaccine efficacy [105] were assumed from published literature and are constant across time and country.

Country-specific transmission to individuals in age group a from individuals in age group j for each time-step t is defined by $\beta_{a,j,t} = \overline{\beta_{a,J,}}(1 + \alpha\, cos(2\pi t))$, where $\overline{\beta_{a,J}}$ is mean transmission from individuals in age group j to age group a, and α is a parameter controlling the magnitude of seasonal fluctuations (assumed 0·15 [101] and constant over time and country). Mean transmission from individuals in age class j to age class a, $\overline{\beta_{a,J}}$, was estimated by rescaling population-adjusted age-contact rates (time constant and country-specific [106]) to reflect the assumed basic reproductive number (R_0) of rubella. R_0 distributions were country-specific and estimated by fitting a dampened exponential model [107] with likelihood-based MCMC to published rubella immunoglobulin G (IgG) seroprevalence data. Model parameters (i.e., R_0) were fit to empirical data, however the large transmission model itself is not directly fit to data. Model uncertainty includes process uncertainty for all epidemiological and demographic transition and uncertainty on the value of R_0.

Age- and time-specific CRS cases were estimated from each country's model output by multiplying the age-specific number of susceptible individuals, the sex ratio of the population, the age-specific fertility rate, the probability of becoming infected over 16 week period, and finally the probability of CRS following rubella infection during the first 16 weeks of pregnancy (estimated 0·59 [108-112]). Fetal and child deaths were estimated directed from the number of CRS cases as 9·3 per 100 live births, and 1·4 per 100 live births, respectively [113].

The model includes process and parameter stochasticity. All epidemiological and demographic processes at each discrete time point are drawn from either a Binomial or Poisson distribution. Parameter stochasticity is based solely on uncertainty in the country-specific rubella basic reproductive number, where in each simulation (i.e., 200 simulations for each country) has a unique

basic reproductive number value drawn from a normal distribution with country-specific log mean and standard deviation.

Yellow Fever – Imperial College London

The Imperial College yellow fever transmission model is a static force of infection (FOI) epidemiological model which was originally published by Garske et al [114]. The model is fitted at the first administrative level or province level for the 34 countries considered endemic for yellow fever in Africa. In each administrative unit, the force of infection is assumed to be constant across the observation period and across age groups. This is analogous to assuming that all yellow fever transmission occurs as a result of spillover events from the sylvatic reservoir. As a result, this model variant includes no herd immunity effects.

The model is estimated from multiple data sources which inform separate components. A generalised linear model, based on environmental covariates, is informed by presence/absence of yellow fever reports between 1984 and 2017 at province level. Reports of yellow fever are based on outbreak reports published by the WHO and on cases reported in the Yellow Fever Surveillance Database (YFSD) managed by WHO-AFRO, to which 21 countries in West and Central Africa contribute [115,116]. The environmental covariates include temperature, enhanced vegetation index, land cover classifications, rainfall estimates and altitude [117-120]. The regression model provides estimates of the probability of yellow fever reports across the endemic zone.

These estimates are then translated to the number of infections by further fitting to data obtained from 15 serological surveys performed in 14 countries in East and Central Africa [121-125]. In each survey location, a static, age-independent force of infection is fitted. This is also informed by estimates of demography and vaccination coverage including historic vaccination campaigns [126-129].

Model components are estimated within a Bayesian framework with adaptive Markov Chain Monte Carlo sampling. In the case of the serological surveys, a log-transform is used to ensure efficient mixing. All estimation was performed in R and convergence of the chains was checked visually. To produce the burden estimates, 200 samples of the posterior predictive distributions of the FOI in each province were taken which we then use to calculate the incidence of infections in each province. The model was validated with external serological surveys conducted in three countries [130].

We use published values of the proportion of infections which are severe and of the CFR to calculate the burden of disease. These proportions, estimated by Johansson, Vasconcelos, and Staples [131], are that 12% [5%, 26%] of infections are severe and that 47% [31%, 62%] of severe infections result in death. However, these estimates remain uncertain since the disease is notoriously misdiagnosed and under-reported. Another area resulting in uncertainty in burden estimates is the heterogeneity in data availability, specifically serological surveys which are not currently available in West Africa. The result of these uncertainties is that burden estimates in West African countries have wide credible intervals and that the burden in general is dominated by that in CFR estimates.

Table iii. Comparison of vaccine related parameters and model types for models of the same pathogen. The pathogen name is given in the first row and column of each table with the different modelling groups' names in the subsequent columns in the first row.

**HepB**	**Goldstein**	**Center for Disease Analysis**		**Imperial College London**
Vaccine efficacy	Birth dose: 95%<br>Second dose: 95%<br>Third dose: 95%	For infants born to mothers with high viral load: - 20% for timely birth dose, 71% for 3 doses with no birth dose and 88% for timely birth dose with three doses for developing chronic HepB. Efficacy against developing acute infection is 10% for timely birth dose, 67% for 3 doses without birth dose and 86% timely birth dose with 3 doses		Birth dose: 83% if mother is HBeAg+; 95% if mother is HBeAg-, HBsAg+<br>Infant vaccine (all three doses): 95%
Vaccine duration	Lifelong	Lifelong for infants receiving timely birth dose with follow-up 3 doses or receiving three doses.		Lifelong
Case fatality ratio	70% from fulminant hepatitis	0·335% for 0-14 years<br>0·45% for 15 years and above (these are deaths only from fulminant hepatitis B)		Age dependant varying from a minimum to maximum of 21·2 – 58·1%
Type of model / Herd effects	Static /no herd effects	Dynamic / herd effects included		Dynamic / herd effects included

**Hib**	**Johns Hopkins University**	**LSHTM**
Vaccine efficacy	93% after third dose (full course of three doses assumed)	First dose: 59%,<br>Second dose: 92%,

		Third dose: 93%
Vaccine duration	Until age 5 as the model only considers up to 5 years	No vaccine waning
Case fatality ratio	N/A – uses overall <5 mortality envelope and disease specific mortality estimates	Country-specific estimates based on Wahl et al [38].
Type of model / Herd effects	Static / no herd effects	Static / no herd effects

**HPV**	**Harvard University**	**LSHTM**
Vaccine efficacy	100% with full dose schedule. Partial course not modeled	100%
Vaccine duration	Lifelong with full dose schedule	Lifelong when doses given 6 months apart
Case fatality ratio	80%	Age and country specific
Type of model / Herd effects	Static / no herd effects	Static / no herd effects

**JE**	**University of Oxford**	**University of Notre Dame**
Vaccine efficacy	100% (single dose)	99·3% (single dose)
Vaccine duration	Lifelong	Lifelong
Case fatality ratio	20 – 30% (for symptomatic cases)	32·9% (95% CI: 5·1%-75·0%)
Type of model / Herd effects	Dynamic / no herd effects	Dynamic /no herd effects

**Measles**	**LSHTM**	**Pennsylvania State University**
Vaccine efficacy	First dose: 85% when given before 1 year and 95% when given after 1 year.<br>After two doses: 98%	85% when dose is administered at 9 months, 93% when administered at 12 months and 99% thereafter
Vaccine duration	Lifelong	Lifelong
Case fatality ratio	Age and country specific [75]. These will be changed to time-varying CFRs informed by Portnoy et al [132]	Age and country specific [75] with CFRs for age above 59 months assumed to be 50% lower than those below 59 months

Type of model / Herd effects	Dynamic / herd effects included	Dynamic / herd effects included

**MenA**	**University of Cambridge**	**Kaiser Permanente Washington**
Vaccine efficacy	90% against disease<br>90% against carriage	75% against colonization<br>100% against invasive disease for the first stage of waning.<br>For the second stage it was<br>25% against effective colonization and<br>90% against disease.
Vaccine duration	10 years	45 years
Case fatality ratio	10%	Age dependant varying from a minimum to maximum 8·6 – 12·2%.
Type of model / Herd effects	Dynamic / herd effects included	Dynamic / herd effects included

**PCV**	**Johns Hopkins University**	**LSHTM**
Vaccine efficacy	58%	First dose: 29%,<br>Second dose: 58%,<br>Third dose: 58%
Vaccine duration	Until age 5 as the model only considers up to 5 years	No vaccine waning
Case fatality ratio	N/A – uses overall <5 mortality envelope and disease specific mortality estimates	Country-specific estimates based on Wahl et al [38]
Type of model / Herd effects	Static / no herd effects	Static / no herd effects

**Rota**	**Johns Hopkins University**	**LSHTM**
Vaccine efficacy	Region specific:<br>87·9% Asia,<br>87·9% North Africa,<br>49·7% Southern Africa, West Africa and East Africa,	Varies by number of doses, under-five mortality strata and duration of follow-up [46]

	82% Eastern Europe,<br>81% Latin America. [35]	
Vaccine duration	Until age 5 as the model only considers up to 5 years	Efficacy decreases over time to be consistent with the year 1 and year 2 estimates [46]
Case fatality ratio	N/A – uses overall <5 mortality envelope and disease specific mortality estimates	Country-specific estimates based on three sources of international estimates [42].
Type of model / Herd effects	Static / no herd effects	Static / no herd effects

**Rubella**	**Public Health England**	**Johns Hopkins University**
Vaccine efficacy	95% (varied 85-99%)	Age dependent with a maximum of 97%
Vaccine duration	Lifelong	Lifelong
Case fatality ratio	30%	9·3% for fetal death<br>1·4% for children
Type of model / Herd effects	Dynamic / herd effects	Dynamic / herd effects

**YF**	**Imperial College London**
Vaccine efficacy	97·5% (95% CI: 82·9-99·7%)
Vaccine duration	Lifelong
Case fatality ratio	47% (95% CI: 31 - 62%)
Type of model / Herd effects	Static / no herd effects

References

1. Polaris Observatory C. Global prevalence, treatment, and prevention of hepatitis B virus infection in 2016: a modelling study. *Lancet Gastroenterol Hepatol* 2018; **3**(6): 383-403.
2. GBD. Global Burden of Disease Collaborative Network. Global Burden of Disease Study 2016 (GBD 2016). Seattle, United States: Institute for Health Metrics and Evaluation (IHME); 2017.
3. Nayagam S, Thursz M, Sicuri E, et al. Requirements for global elimination of hepatitis B: a modelling study. *Lancet Infect Dis* 2016; **16**(12): 1399-408.

4. Mendy ME, Fortuin M, Hall AJ, Jack AD, Whittle HC. Hepatitis B virus DNA in relation to duration of hepatitis B surface antigen carriage. *Br J Biomed Sci* 1999; **56**(1): 34-8.
5. Mendy ME, McConkey SJ, Sande van der MA, et al. Changes in viral load and HBsAg and HBeAg status with age in HBV chronic carriers in The Gambia. *Virol J* 2008; **5**: 49.
6. Keane E, Funk AL, Shimakawa Y. Systematic review with meta-analysis: the risk of mother-to-child transmission of hepatitis B virus infection in sub-Saharan Africa. *Aliment Pharmacol Ther* 2016; **44**(10): 1005-17.
7. Edmunds WJ, Medley GF, Nokes DJ, Hall AJ, Whittle HC. The influence of age on the development of the hepatitis B carrier state. *Proc Biol Sci* 1993; **253**(1337): 197-201.
8. Ott JJ, Stevens GA, Groeger J, Wiersma ST. Global epidemiology of hepatitis B virus infection: new estimates of age-specific HBsAg seroprevalence and endemicity. *Vaccine* 2012a; **30**(12): 2212-9.
9. Ott JJ, Stevens GA, Wiersma ST. The risk of perinatal hepatitis B virus transmission: hepatitis B e antigen (HBeAg) prevalence estimates for all world regions. *BMC Infect Dis* 2012b; **12**: 131.
10. Lagarias JC, Reeds JA, Wright MH, Wright PE. Convergence properties of the Nelder-Mead simplex method in low dimensions. *Siam J Optimiz* 1998; **9**(1): 112-47.
11. Goldstein ST, Zhou F, Hadler SC, Bell BP, Mast EE, Margolis HS. A mathematical model to estimate global hepatitis B disease burden and vaccination impact. *Int J Epidemiol* 2005; **34**(6): 1329-39.
12. Walker N, Tam Y, Friberg IK. Overview of the Lives Saved Tool (LiST). *BMC Public Health* 2013; **13 Suppl 3**: S1.
13. UNWPP. World Population Prospects - Population Division - United Nations. 2017.
14. CME. Child mortality estimates. 18 March 2019. https://childmortality.org/.
15. GHO. Causes of child death. Global Health Observatory data repository. WHO. http://apps.who.int/gho/data/node.main.COCD?lang=en (accessed 18 March 2019).
16. Liu L, Oza S, Hogan D, et al. Global, regional, and national causes of under-5 mortality in 2000-15: an updated systematic analysis with implications for the Sustainable Development Goals. *Lancet* 2016; **388**(10063): 3027-35.
17. Sachdev HPS, Hall A, Walker N. Development and use of the Lives Saved Tool (LiST): A model to estimate the impact of scaling up proven interventions on maternal, neonatal and child mortality. *Int J Epidemiol* 2010; (39 Suppl 1).
18. Fox MJ, Marterell R, Van den Broek N, Walker N. Technical inputs, enhancements and applications of the Lives Saved Tool (LiST). *BMC Public Health* 2011; **11** (Suppl 3).
19. Walker N. The Lives Saved Tool in 2013: new capabilities and applications. *BMC Public Health* 2013; **11**(Suppl 3): S1.
20. Clemont A, Walker N. Nutrition Interventions in the Lives Saved Tool (LiST). *J Nutr* 2017; **147**(11): 2132S-40S.
21. Walker N, Friberg I. The Lives Saved Tool in 2017: Updates, Applications, and Future Directions. *BMC Public Health* 2017; **17**(Suppl 4).
22. Amouzou A, Richard SA, Friberg IK, et al. How well does LiST capture mortality by wealth quintile? A comparison of measured versus modelled mortality rates among children under-five in Bangladesh. *Int J Epidemiol* 2010; **39 Suppl 1**: i186-92.
23. Friberg IK, Bhutta ZA, Darmstadt GL, et al. Comparing modelled predictions of neonatal mortality impacts using LiST with observed results of community-based intervention trials in South Asia. *Int J Epidemiol* 2010; **39 Suppl 1**: i11-20.

24. Hazel E, Gilroy K, Friberg I, Black RE, Bryce J, Jones G. Comparing modelled to measured mortality reductions: applying the Lives Saved Tool to evaluation data from the Accelerated Child Survival Programme in West Africa. *Int J Epidemiol* 2010; **39 Suppl 1**: i32-9.
25. Larsen DA, Friberg IK, Eisele TP. Comparison of Lives Saved Tool model child mortality estimates against measured data from vector control studies in sub-Saharan Africa. *BMC Public Health* 2011; **11 Suppl 3**: S34.
26. Ricca J, Prosnitz D, Perry H, et al. Comparing estimates of child mortality reduction modelled in LiST with pregnancy history survey data for a community-based NGO project in Mozambique. *BMC Public Health* 2011; **11 Suppl 3**: S35.
27. Victora CG, Barros AJ, Malpica-Llanos T, Walker N. How within-country inequalities and co-coverage may affect LiST estimates of lives saved by scaling up interventions. *BMC Public Health* 2013; **13 Suppl 3**: S24.
28. Rudan I, O'Brien KL, Nair H, et al. Epidemiology and etiology of childhood pneumonia in 2010: estimates of incidence, severe morbidity, mortality, underlying risk factors and causative pathogens for 192 countries. *J Glob Health* 2013; **3**(1): 010401.
29. Davis S, Feikin D, Johnson HL. The effect of Haemophilus influenzae type B and pneumococcal conjugate vaccines on childhood meningitis mortality: a systematic review. *BMC Public Health* 2013; **13 Suppl 3**: S21.
30. O'Brien KL, Wolfson LJ, Watt JP, et al. Burden of disease caused by Streptococcus pneumoniae in children younger than 5 years: global estimates. *Lancet* 2009; **374**(9693): 893-902.
31. Watt JP, Wolfson LJ, O'Brien KL, et al. Burden of disease caused by Haemophilus influenzae type b in children younger than 5 years: global estimates. *Lancet* 2009; **374**(9693): 903-11.
32. Lucero MG, Dulalia VE, Parreno RN, et al. Pneumococcal conjugate vaccines for preventing vaccine-type invasive pneumococcal disease and pneumonia with consolidation on x-ray in children under two years of age. *Cochrane Database Syst Rev* 2004; (4): CD004977.
33. Griffiths UK, Clark A, Gessner B, et al. Dose-specific efficacy of Haemophilus influenzae type b conjugate vaccines: a systematic review and meta-analysis of controlled clinical trials. *Epidemiol Infect* 2012; **140**(8): 1343-55.
34. Fischer Walker CL, Rudan I, Liu L, et al. Global burden of childhood pneumonia and diarrhoea. *Lancet* 2013; **381**(9875): 1405-16.
35. Fischer Walker CL, Black RE. Rotavirus vaccine and diarrhea mortality: quantifying regional variation in effect size. *BMC Public Health* 2011; **11 Suppl 3**: S16.
36. Clark AD, Tate J, Parashar U, Jit M. Mortality reduction benefits and intussusception risks of rotavirus vaccination in 135 low- and middle-income countries: evaluation of current and alternative schedules *(submitted)* submitted.
37. Clark A, Jauregui B, Griffiths U, et al. TRIVAC decision-support model for evaluating the cost-effectiveness of Haemophilus influenzae type b, pneumococcal and rotavirus vaccination. *Vaccine* 2013; **31 Suppl 3**: C19-29.
38. Wahl B, O'Brien KL, Greenbaum A, et al. Burden of Streptococcus pneumoniae and Haemophilus influenzae type b disease in children in the era of conjugate vaccines: global, regional, and national estimates for 2000-15. *Lancet Glob Health* 2018; **6**(7): e744-e57.

39. CDC. Pneumococcal disease - clinicial features - acute otitis media. https://www.cdc.gov/pneumococcal/clinicians/clinical-features.html (accessed December 2017).
40. Monasta L, Ronfani L, Marchetti F, et al. Burden of disease caused by otitis media: systematic review and global estimates. *PLoS One* 2012; **7**(4): e36226.
41. Edmond K, Clark A, Korczak VS, Sanderson C, Griffiths UK, Rudan I. Global and regional risk of disabling sequelae from bacterial meningitis: a systematic review and meta-analysis. *Lancet Infect Dis* 2010; **10**(5): 317-28.
42. Clark A, Black R, Tate J, et al. Estimating global, regional and national rotavirus deaths in children aged <5 years: Current approaches, new analyses and proposed improvements. *PLoS One* 2017; **12**(9): e0183392.
43. Bilcke J, Van Damme P, Van Ranst M, Hens N, Aerts M, Beutels P. Estimating the incidence of symptomatic rotavirus infections: a systematic review and meta-analysis. *PLoS One* 2009; **4**(6): e6060.
44. Hasso-Agopsowicz M, Ladva CN, Lopman B, et al. Global review of the age distribution of rotavirus disease in children aged <5 years before the introduction of rotavirus vaccination. *Clin Infect Dis* 2019.
45. Clark A, Sanderson C. Timing of children's vaccinations in 45 low-income and middle-income countries: an analysis of survey data. *Lancet* 2009; **373**(9674): 1543-9.
46. Patel MM, Clark AD, Sanderson CF, Tate J, Parashar UD. Removing the age restrictions for rotavirus vaccination: a benefit-risk modeling analysis. *Plos Med* 2012; **9**(10): e1001330.
47. Lucero MG, Dulalia VE, Nillos LT, et al. Pneumococcal conjugate vaccines for preventing vaccine-type invasive pneumococcal disease and X-ray defined pneumonia in children less than two years of age. *Cochrane Database Syst Rev* 2009; (4): CD004977.
48. Davis R. Teaching Note—Teaching Project Simulation in Excel Using PERT-Beta Distributions. *INFORMS Transactions on Education* 2008; **8**(3): 139-48.
49. Goldie SJ, O'Shea M, Campos NG, Diaz M, Sweet S, Kim SY. Health and economic outcomes of HPV 16,18 vaccination in 72 GAVI-eligible countries. *Vaccine* 2008; **26**(32): 4080-93.
50. Goldie SJ, Kim JJ, Kobus K, et al. Cost-effectiveness of HPV 16, 18 vaccination in Brazil. *Vaccine* 2007; **25**(33): 6257-70.
51. Kim JJ, Kuntz KM, Stout NK, et al. Multiparameter calibration of a natural history model of cervical cancer. *Am J Epidemiol* 2007; **166**(2): 137-50.
52. Campos NG, Kim JJ, Castle PE, et al. Health and economic impact of HPV 16/18 vaccination and cervical cancer screening in Eastern Africa. *Int J Cancer* 2012; **130**(11): 2672-84.
53. Murray CJL, Lopez AD. Estimating causes of death: new methods and global and regional applications for 1990. In: Murray CJL, Lopez AD, eds. The Global Burden of Disease. Cambridge, MA: harvard University Press; 1996: 117-200.
54. Jit M, Brisson M, Portnoy A, Hutubessy R. Cost-effectiveness of female human papillomavirus vaccination in 179 countries: a PRIME modelling study. *Lancet Glob Health* 2014; **2**(7): e406-14.
55. Schiller JT, Castellsague X, Garland SM. A review of clinical trials of human papillomavirus prophylactic vaccines. *Vaccine* 2012; **30 Suppl 5**: F123-38.

56. Jit M, Brisson M. Potential lives saved in 73 countries by adopting multi-cohort vaccination of 9-14-year-old girls against human papillomavirus. *Int J Cancer* 2018; **143**(2): 317-23.
57. Quan TM, Thao TTN, Duy NM, Nhat TM, Clapham H. Estimates of the global burden of Japanese encephalitis and the impact of vaccination from 2000-2015. *Elife* 2020; **9**.
58. Quan TM, Thao TTN, Duy NM, Nhat TM, Clapham H. Estimates of the global burden of Japanese Encephalitis and the impact of vaccination from 2000-2015. *medRxiv* 2019: 19006940.
59. Campbell GL, Hills SL, Fischer M, et al. Estimated global incidence of Japanese encephalitis: a systematic review. *Bull World Health Organ* 2011; **89**(10): 766-74, 74A-74E.
60. SAGE Working Group on Japanese encephalitis vaccines. Background Paper on Japanese Encephalitis Vaccines. Geneva WHO, 2014.
61. van den Hurk AF, Ritchie SA, Mackenzie JS. Ecology and Geographical Expansion of Japanese Encephalitis Virus. *Annu Rev Entomol* 2009; **54**: 17-35.
62. Longbottom J, Browne AJ, Pigott DM, et al. Mapping the spatial distribution of the Japanese encephalitis vector, Culex tritaeniorhynchus Giles, 1901 (Diptera: Culicidae) within areas of Japanese encephalitis risk. *Parasite Vector* 2017; **10**.
63. Gumma MK, Nelson A, Thenkabail PS, Singh AN. Mapping rice areas of South Asia using MODIS multitemporal data. *J Appl Remote Sens* 2011; **5**.
64. Robinson TP, Wint GRW, Conchedda G, et al. Mapping the Global Distribution of Livestock. *Plos One* 2014; **9**(5).
65. Hay SI, Abajobir AA, Abate KH, et al. Global, regional, and national disability-adjusted life-years (DALYs) for 333 diseases and injuries and healthy life expectancy (HALE) for 195 countries and territories, 1990-2016: a systematic analysis for the Global Burden of Disease Study 2016. *Lancet* 2017; **390**(10100): 1260-344.
66. Verguet S, Johri M, Morris SK, Gauvreau CL, Jha P, Jit M. Controlling measles using supplemental immunization activities: A mathematical model to inform optimal policy. *Vaccine* 2015; **33**(10): 1291-6.
67. Verguet S, Jones EO, Johri M, et al. Characterizing measles transmission in India: a dynamic modeling study using verbal autopsy data. *Bmc Med* 2017; **15**.
68. Johri M, Verguet S, Morris SK, et al. Adding interventions to mass measles vaccinations in India. *B World Health Organ* 2016; **94**(10): 718-27.
69. Mossong J, Hens N, Jit M, et al. Social contacts and mixing patterns relevant to the spread of infectious diseases. *Plos Med* 2008; **5**(3): 381-91.
70. Uzicanin A, Zimmerman L. Field Effectiveness of Live Attenuated Measles-Containing Vaccines: A Review of Published Literature. *J Infect Dis* 2011; **204**: S133-S48.
71. Finkenstadt BF, Grenfell BT. Time series modelling of childhood diseases. *Applied Statistics*
2000; **49**(Part 2): 187-205.
72. Anderson RM, May RM. Infectious Disease of Humans: Dynamics and Control: Oxford Univerity Press; 1991.
73. Eilertson KE, Fricks J, Ferrari MJ. Estimation and prediction for a mechanistic model of measles transmission using particle filtering and maximum likelihood estimation. *Stat Med* 2019; **(In press)**.
74. Simons E, Ferrari M, Fricks J, et al. Assessment of the 2010 global measles mortality reduction goal: results from a model of surveillance data. *Lancet* 2012; **379**(9832): 2173-8.

75. Wolfson LJ, Grais RF, Luquero FJ, Birmingham ME, Strebel PM. Estimates of measles case fatality ratios: a comprehensive review of community-based studies. *Int J Epidemiol* 2009; **38**(1): 192-205.
76. Karachaliou A, Conlan AJ, Preziosi MP, Trotter CL. Modeling Long-term Vaccination Strategies With MenAfriVac in the African Meningitis Belt. *Clin Infect Dis* 2015; **61 Suppl 5**: S594-600.
77. Campagne G, Schuchat A, Djibo S, Ousseini A, Cisse L, Chippaux JP. Epidemiology of bacterial meningitis in Niamey, Niger, 1981-96. *Bull World Health Organ* 1999; **77**(6): 499-508.
78. Irving TJ, Blyuss KB, Colijn C, Trotter CL. Modelling meningococcal meningitis in the African meningitis belt. *Epidemiol Infect* 2012; **140**(5): 897-905.
79. Tapia MD, Findlow H, Idoko OT, et al. Antibody Persistence 1-5 Years Following Vaccination With MenAfriVac in African Children Vaccinated at 12-23 Months of Age. *Clin Infect Dis* 2015; **61 Suppl 5**: S514-20.
80. Stephens DS, Greenwood B, Brandtzaeg P. Epidemic meningitis, meningococcaemia, and Neisseria meningitidis. *Lancet* 2007; **369**(9580): 2196-210.
81. Lingani C, Bergeron-Caron C, Stuart JM, et al. Meningococcal Meningitis Surveillance in the African Meningitis Belt, 2004-2013. *Clin Infect Dis* 2015; **61 Suppl 5**: S410-5.
82. Tartof S, Cohn A, Tarbangdo F, et al. Identifying optimal vaccination strategies for serogroup A Neisseria meningitidis conjugate vaccine in the African meningitis belt. *PLoS One* 2013; **8**(5): e63605.
83. Kristiansen PA, Diomande F, Wei SC, et al. Baseline meningococcal carriage in Burkina Faso before the introduction of a meningococcal serogroup A conjugate vaccine. *Clin Vaccine Immunol* 2011; **18**(3): 435-43.
84. Boisier P, Mainassara HB, Sidikou F, Djibo S, Kairo KK, Chanteau S. Case-fatality ratio of bacterial meningitis in the African meningitis belt: we can do better. *Vaccine* 2007; **25 Suppl 1**: A24-9.
85. Belcher DW, Sherriff AC, Nimo KP, et al. Meningococcal meningitis in northern Ghana: epidemiology and control measures. *Am J Trop Med Hyg* 1977; **26**(4): 748-55.
86. Traore Y, Tameklo TA, Njanpop-Lafourcade BM, et al. Incidence, seasonality, age distribution, and mortality of pneumococcal meningitis in Burkina Faso and Togo. *Clin Infect Dis* 2009; **48 Suppl 2**: S181-9.
87. Varaine F, Caugant DA, Riou JY, et al. Meningitis outbreaks and vaccination strategy. *Trans R Soc Trop Med Hyg* 1997; **91**(1): 3-7.
88. Epidemics of meningococcal disease. African meningitis belt, 2001. *Wkly Epidemiol Rec* 2001; **76**(37): 282-8.
89. World Health Organization G. Enhanced surveillance of epidemic meningococcal meningitis in Africa: a three-year experience. *Wkly Epidemiol Rec* 2005; **80**(37): 313-20.
90. Goldschneider I, Gotschlich EC, Artenstein MS. Human immunity to the meningococcus. II. Development of natural immunity. *J Exp Med* 1969; **129**(6): 1327-48.
91. Jackson ML, Diallo AO, Medah I, et al. Initial validation of a simulation model for estimating the impact of serogroup A Neisseria meningitidis vaccination in the African meningitis belt. *PLoS One* 2018; **13**(10): e0206117.
92. Vynnycky E, Yoshida LM, Huyen DT, et al. Modeling the impact of rubella vaccination in Vietnam. *Hum Vaccin Immunother* 2016; **12**(1): 150-8.

93. Vynnycky E, Adams EJ, Cutts FT, et al. Using Seroprevalence and Immunisation Coverage Data to Estimate the Global Burden of Congenital Rubella Syndrome, 1996-2010: A Systematic Review. *PLoS One* 2016; **11**(3): e0149160.
94. Vynnycky E, Papadopoulos T, Angelis K. The impact of Measles-Rubella vaccination on the morbidity and mortality from Congenital Rubella Syndrome in 92 countries. *Hum Vaccin Immunother* 2019; **15**(2): 309-16.
95. Schenzle D. An age-structured model of pre- and post-vaccination measles transmission. *IMA J Math Appl Med Biol* 1984; **1**(2): 169-91.
96. UN Statistics Division UNPD. World Population Prospects. 2017.
97. Mossong J, Hens N, Jit M, et al. Social contacts and mixing patterns relevant to the spread of infectious diseases. *Plos Med* 2008; **5**(3): e74.
98. Vynnycky E, Gay NJ, Cutts FT. The predicted impact of private sector MMR vaccination on the burden of Congenital Rubella Syndrome. *Vaccine* 2003; **21**(21-22): 2708-19.
99. Simons EA, Reef SE, Cooper LZ, Zimmerman L, Thompson KM. Systematic Review of the Manifestations of Congenital Rubella Syndrome in Infants and Characterization of Disability-Adjusted Life Years (DALYs). *Risk Anal* 2016; **36**(7): 1332-56.
100. Bank W. World Development Indicators. 2017. https://data.worldbank.org/data-catalogue/world-development-indicators.
101. Metcalf CJ, Lessler J, Klepac P, Cutts F, Grenfell BT. Impact of birth rate, seasonality and transmission rate on minimum levels of coverage needed for rubella vaccination. *Epidemiol Infect* 2012a; **140**(12): 2290-301.
102. Metcalf CJ, Lessler J, Klepac P, Morice A, Grenfell BT, Bjornstad ON. Structured models of infectious disease: inference with discrete data. *Theor Popul Biol* 2012b; **82**(4): 275-82.
103. WHO-UNICEF. World Health Organization & UNICEF. Estimates of National Immunization Coverage (WUENIC), estimates for 1980 to 2016. 2017.
104. Nicoara C, Zach K, Trachsel D, Germann D, Matter L. Decay of passively acquired maternal antibodies against measles, mumps, and rubella viruses. *Clin Diagn Lab Immunol* 1999; **6**(6): 868-71.
105. Boulianne N, De Serres G, Ratnam S, Ward BJ, Joly JR, Duval B. Measles, mumps, and rubella antibodies in children 5-6 years after immunization: effect of vaccine type and age at vaccination. *Vaccine* 1995; **13**(16): 1611-6.
106. Prem K, Cook AR, Jit M. Projecting social contact matrices in 152 countries using contact surveys and demographic data. *PLoS Comput Biol* 2017; **13**(9): e1005697.
107. Farrington CP. Modeling Forces of Infection for Measles, Mumps and Rubella. *Stat Med* 1990; **9**(8): 953-67.
108. Enders G, Nickerl-Pacher U, Miller E, Cradock-Watson JE. Outcome of confirmed periconceptional maternal rubella. *Lancet* 1988; **1**(8600): 1445-7.
109. Ghidini A, Lynch L. Prenatal diagnosis and significance of fetal infections. *West J Med* 1993; **159**(3): 366-73.
110. Grillner L, Forsgren M, Barr B, Bottiger M, Danielsson L, De Verdier C. Outcome of rubella during pregnancy with special reference to the 17th-24th weeks of gestation. *Scand J Infect Dis* 1983; **15**(4): 321-5.
111. Miller E, Cradock-Watson JE, Pollock TM. Consequences of confirmed maternal rubella at successive stages of pregnancy. *Lancet* 1982; **2**(8302): 781-4.

112. Munro ND, Sheppard S, Smithells RW, Holzel H, Jones G. Temporal relations between maternal rubella and congenital defects. *Lancet* 1987; **2**(8552): 201-4.
113. Siegel M, Fuerst HT, Peress NS. Comparative fetal mortality in maternal virus diseases. A prospective study on rubella, measles, mumps, chicken pox and hepatitis. *N Engl J Med* 1966; **274**(14): 768-71.
114. Garske T, Van Kerkhove MD, Yactayo S, et al. Yellow Fever in Africa: estimating the burden of disease and impact of mass vaccination from outbreak and serological data. *Plos Med* 2014; **11**(5): e1001638.
115. WHO. Disease Outbreak News Since 1996. 2014. http://www.who.int/csr/don/en/.
116. WHO. Weekly Epidemiological Record, 2009.
117. Garske T, Ferguson NM, Ghani AC. Estimating air temperature and its influence on malaria transmission across Africa. *PLoS One* 2013; **8**(2): e56487.
118. Hijmans RJ, Cameron SE, Parra JL, Jones PG, Jarvis A. The Worldclim Interpolated Global Terrestrial Climate Surfaces. Version 1.3. 2004. http://biogeo.berkeley.edu/ WORLDCLIM.
119. NASA. NASA Land Processes Distributed Active Archive Center (LP DAAC) USGS/Earth Resources Observation and Science (EROS) Center. 2001. https://earthdata.nasa.gov/about/daacs/daac-lpdaac.
120. Xie PP, Arkin PA. Analyses of global monthly precipitation using gauge observations, satellite estimates, and numerical model predictions. *J Climate* 1996; **9**(4): 840-58.
121. Kuniholm MH, Wolfe ND, Huang CY, et al. Seroprevalence and distribution of Flaviviridae, Togaviridae, and Bunyaviridae arboviral infections in rural Cameroonian adults. *Am J Trop Med Hyg* 2006; **74**(6): 1078-83.
122. Tsai TF, Lazuick JS, Ngah RW, Mafiamba PC, Quincke G, Monath TP. Investigation of a possible yellow fever epidemic and serosurvey for flavivirus infections in northern Cameroon, 1984. *Bull World Health Organ* 1987; **65**(6): 855-60.
123. Werner GT, Huber HC, Fresenius K. Prevalence of Antibodies against Yellow-Fever in Northern Zaire. *Ann Soc Belg Med Tr* 1985; **65**(1): 91-3.
124. Merlin M, Josse R, Kouka-Bemba D, et al. [Evaluation of immunological and entomotological indices of yellow fever in Pointe-Noire, People's Republic of Congo]. *Bull Soc Pathol Exot Filiales* 1986; **79**(2): 199-206.
125. Staples JE, Diallo M, Janusz KB, et al. Yellow fever risk assessment in the Central African Republic. *Trans R Soc Trop Med Hyg* 2014; **108**(10): 608-15.
126. Moreau JP, Girault G, Drame I, Perraut R. [Reemergence of yellow fever in West Africa: lessons from the past, advocacy for a control program]. *Bull Soc Pathol Exot* 1999; **92**(5): 333-6.
127. WHO/UNICEF. Estimates of National Immunization Coverage (WUENIC) 2015. 2015. http://apps.who.int/immunization_monitoring/globalsummary/timeseries/tswucoverageyfv.html.
128. Hamlet A, Jean K, Yactayo S, et al. POLICI: A web application for visualising and extracting yellow fever vaccination coverage in Africa. *Vaccine* 2019; **37**(11): 1384-8.
129. Durieux C. Mass yellow fever vaccination in French Africa south of the Sahara. *Monograph Series World Health Organization* 1956: 115-22.
130. Jean K, Hamlet A, Benzler J, et al. Eliminating yellow fever epidemics in Africa: Vaccine demand forecast and impact modelling. *PLoS Negl Trop Dis* 2020; **14**(5): e0008304.

131. Johansson MA, Vasconcelos PF, Staples JE. The whole iceberg: estimating the incidence of yellow fever virus infection from the number of severe cases. *Trans R Soc Trop Med Hyg* 2014; **108**(8): 482-7.
132. Portnoy A, Jit M, Helleringer S, Verguet S. Impact of measles supplementary immunization activities on reaching children missed by routine programs. *Vaccine* 2018; **36**(1): 170-8.

Sensitivity of impact estimates to projected coverage

We performed a sensitivity analysis to show how impact estimates differ under different coverage assumptions. We compare the impact ratios (deaths averted/fully vaccinated people) under the reported and projected coverage scenario used in this study, and the impact ratios calculated under an alternative coverage scenario called best-case, which assumes 90% of routine coverage (or historical highest if greater than 90%), and a one-off campaign with 90% coverage for the 2020-2030 period. The comparison was done for the PINE countries, Ethiopia (ETH), India (IND), Nigeria (NGA) and Pakistan (PAK).

Best-case coverage scenario:

- Routine coverage: was assumed to be 90% (or historical highest if greater than 90%)
- Campaign coverage: a one-off campaign assumed to be 90%

Table iv: Estimated total deaths averted per thousand individuals vaccinated across the 2020-2030 period in all ages under the reported and projected coverage and best-case coverage scenarios. Estimates for both calendar year and year of birth views are shown. Not all pathogens are endemic to all the PINE countries, hence the NA values.

Disease	Scenario	Calendar year view				Lifetime view			
		ETH	IND	NGA	PAK	ETH	IND	NGA	PAK
HepB	Default	0.4	0.2	1.6	0.5	7.4	3.5	24.6	5.8
	Best-case	0.2	0.2	1.1	0.2	4.6	3.2	19.9	3.3
Hib	Default	3.2	2.1	3.5	2.9	3.2	2.1	3.7	2.9
	Best-case	3.2	2.1	3.7	2.9	3.2	2.1	3.8	2.9
HPV	Default	0.1	0	0	0	14.4	12.1	13.2	4.4
	Best-case	0.1	0.1	0	0	14.4	12.1	13.3	4.4
JE	Default	NA	0.2	NA	0	NA	0.4	NA	0.1
	Best-case	NA	0.3	NA	0.1	NA	0.4	NA	0.1
Measles	Default	12.6	7.7	17.2	5.2	15.2	7.7	19.1	5.9
	Best-case	16.3	7.5	22.5	7.6	17.1	7.5	22.5	7.7
MenA	Default	0.7	NA	0.9	NA	1.3	NA	1.3	NA
	Best-case	0.9	NA	0.6	NA	1.1	NA	1	NA
PCV	Default	2.9	1.9	3.4	2.9	2.9	2	3.7	2.9
	Best-case	2.9	2	3.7	2.9	2.9	2	3.7	2.9
Rota	Default	0.5	0.4	2.1	0.6	0.5	0.4	2.4	0.6
	Best-case	0.5	0.4	2.3	0.6	0.5	0.4	2.4	0.6
Rubella	Default	0.1	0.2	NA	0	0.1	0.2	NA	0.1
	Best-case	0.1	0.2	0.2	0.1	0.1	0.2	0.2	0.1
YF	Default	0.1	NA	1.5	NA	0.7	NA	9.4	NA
	Best-case	0.5	NA	5.1	NA	0.8	NA	10.2	NA

Tables

Table S1. List of 98 low and middle income countries included in the analysis, including the 73 countries currently eligible for Gavi support. WHO region corresponds to the WHO Regional Offices in Africa (AFRO), Eastern Mediterranean (EMRO), Europe (EURO), South East Asia (SEARO) and Western Pacific (WPRO).

Country code	Country name	Gavi 73	WHO region
AFG	Afghanistan	TRUE	EMRO
AGO	Angola	TRUE	AFRO
ALB	Albania	FALSE	EURO
ARM	Armenia	TRUE	EURO
AZE	Azerbaijan	TRUE	EURO
BDI	Burundi	TRUE	AFRO
BEN	Benin	TRUE	AFRO
BFA	Burkina Faso	TRUE	AFRO
BGD	Bangladesh	TRUE	SEARO
BIH	Bosnia and Herzegovina	FALSE	EURO
BLZ	Belize	FALSE	PAHO
BOL	Bolivia	TRUE	PAHO
BTN	Bhutan	TRUE	SEARO
CAF	Central African Republic	TRUE	AFRO
CHN	China	FALSE	WPRO
CIV	Cote d'Ivoire	TRUE	AFRO
CMR	Cameroon	TRUE	AFRO
COD	Democratic Republic of the Congo	TRUE	AFRO
COG	Congo	TRUE	AFRO
COM	Comoros	TRUE	AFRO
CPV	Cabo Verde	FALSE	AFRO
CUB	Cuba	TRUE	PAHO
DJI	Djibouti	TRUE	EMRO
EGY	Egypt	FALSE	EMRO
ERI	Eritrea	TRUE	AFRO
ETH	Ethiopia	TRUE	AFRO

FJI	Fiji	FALSE	WPRO
FSM	Federated States of Micronesia	FALSE	WPRO
GEO	Georgia	TRUE	EURO
GHA	Ghana	TRUE	AFRO
GIN	Guinea	TRUE	AFRO
GMB	Gambia	TRUE	AFRO
GNB	Guinea-Bissau	TRUE	AFRO
GTM	Guatemala	FALSE	PAHO
GUY	Guyana	TRUE	PAHO
HND	Honduras	TRUE	PAHO
HTI	Haiti	TRUE	PAHO
IDN	Indonesia	TRUE	SEARO
IND	India	TRUE	SEARO
IRQ	Iraq	FALSE	EMRO
KEN	Kenya	TRUE	AFRO
KGZ	Kyrgyzstan	TRUE	EURO
KHM	Cambodia	TRUE	WPRO
KIR	Kiribati	TRUE	WPRO
LAO	Laos	TRUE	WPRO
LBR	Liberia	TRUE	AFRO
LKA	Sri Lanka	TRUE	SEARO
LSO	Lesotho	TRUE	AFRO
MAR	Morocco	FALSE	EMRO
MDA	Moldova	TRUE	EURO
MDG	Madagascar	TRUE	AFRO
MHL	Marshall Islands	FALSE	WPRO
MLI	Mali	TRUE	AFRO
MMR	Myanmar	TRUE	SEARO
MNG	Mongolia	TRUE	WPRO
MOZ	Mozambique	TRUE	AFRO
MRT	Mauritania	TRUE	AFRO
MWI	Malawi	TRUE	AFRO

NER	Niger	TRUE	AFRO
NGA	Nigeria	TRUE	AFRO
NIC	Nicaragua	TRUE	PAHO
NPL	Nepal	TRUE	SEARO
PAK	Pakistan	TRUE	EMRO
PHL	Philippines	FALSE	WPRO
PNG	Papua New Guinea	TRUE	WPRO
PRK	North Korea	TRUE	SEARO
PRY	Paraguay	FALSE	PAHO
PSE	Palestine	FALSE	PAHO
RWA	Rwanda	TRUE	AFRO
SDN	Sudan	TRUE	EMRO
SEN	Senegal	TRUE	AFRO
SLB	Solomon Islands	TRUE	WPRO
SLE	Sierra Leone	TRUE	AFRO
SLV	El Salvador	FALSE	PAHO
SOM	Somalia	TRUE	EMRO
SSD	South Sudan	TRUE	AFRO
STP	Sao Tome and Principe	TRUE	AFRO
SWZ	Swaziland	FALSE	AFRO
SYR	Syria	FALSE	EMRO
TCD	Chad	TRUE	AFRO
TGO	Togo	TRUE	AFRO
TJK	Tajikistan	TRUE	EURO
TKM	Turkmenistan	FALSE	EURO
TLS	Timor-Leste	TRUE	SEARO
TON	Tonga	FALSE	WPRO
TUN	Tunisia	FALSE	EMRO
TUV	Tuvalu	FALSE	WPRO
TZA	Tanzania	TRUE	AFRO
UGA	Uganda	TRUE	AFRO
UKR	Ukraine	TRUE	EURO

UZB	Uzbekistan	TRUE	EURO
VNM	Viet Nam	TRUE	WPRO
VUT	Vanuatu	FALSE	WPRO
WSM	Samoa	FALSE	WPRO
XK	Kosovo	FALSE	EURO
YEM	Yemen	TRUE	EMRO
ZMB	Zambia	TRUE	AFRO
ZWE	Zimbabwe	TRUE	AFRO

Table S2. Number of countries eligible for Gavi support for routine vaccinations for each pathogen and each year from 2000 to 2018. MCV2: measles-containing vaccine, second dose; PCV3: pneumococcal conjugate vaccine 3rd dose; RCV2: rubella-containing vaccine, second dose.

Year	HepB	Hib3	HPV	JE	MCV2	MenA	PCV3	RCV2	Rota	Rubella	YF
2000	8	0	0	0	0	0	0	0	0	0	0
2001	23	1	0	0	0	0	0	0	0	0	1
2002	41	6	0	0	0	0	0	0	0	0	6
2003	43	6	0	0	0	0	0	0	0	0	9
2004	45	6	0	0	0	0	0	0	0	0	14
2005	50	13	0	0	0	0	0	0	0	0	14
2006	54	14	0	0	0	0	0	0	0	0	14
2007	56	18	0	0	1	0	0	0	0	0	14
2008	58	28	0	0	2	0	0	0	1	0	16
2009	63	48	0	0	2	0	0	0	2	0	16
2010	64	56	0	0	2	0	2	0	4	0	16
2011	63	58	0	0	2	0	15	0	5	0	16
2012	66	61	0	0	6	0	19	0	10	0	16
2013	68	67	0	0	8	0	30	0	15	0	16
2014	68	67	2	0	13	0	42	0	32	1	16
2015	68	68	2	0	17	0	53	0	37	12	16
2016	68	68	3	1	19	1	55	0	40	13	18
2017	67	67	13	2	20	8	57	21	43	21	16
2018	67	67	17	4	22	11	59	22	48	24	17

Table S3(a): Estimated total DALYs averted (in millions) by vaccination and DALYs averted per thousand individuals vaccinated in different time periods across the 98 countries considered, stratified by pathogen. Both all age and under-5 DALYs averted are shown. The values are mean estimates and ranges are 95% credible intervals (2.5 and 97.5 quantiles). Estimates quoted to two significant figures.

Disease	Time	DALYs averted (millions)	DALYs averted per 1000 vaccinated individuals	DALYs averted (millions), < 5	DALYs averted per 1000 vaccinated individuals, <5
HepB	2000-2019	61 (13-160)	35 (7.7-90)	29 (4.6-99)	17 (2.7-58)
HepB	2020-2030	130 (29-230)	77 (17-140)	24 (4.3-80)	15 (2.6-49)
HepB	2000-2030	190 (42-380)	56 (12-120)	53 (8.9-180)	16 (2.6-54)
Hib	2000-2019	100 (45-160)	170 (73-250)	100 (45-160)	170 (73-250)
Hib	2020-2030	140 (47-220)	160 (56-260)	140 (47-220)	160 (56-260)
Hib	2000-2030	240 (94-370)	160 (64-260)	240 (94-370)	160 (64-260)
HPV	2000-2019	0.0011 (0.00051-0.0021)	0 (0-0)	0 (0-0)	0 (0-0)
HPV	2020-2030	0.58 (0.27-0.93)	1.8 (0.8-2.8)	0 (0-0)	0 (0-0)
HPV	2000-2030	0.58 (0.27-0.93)	1.1 (0.5-1.8)	0 (0-0)	0 (0-0)
JE	2000-2019	8.5 (0.54-18)	19 (1.1-39)	6.1 (0.39-13)	21 (1.3-43)
JE	2020-2030	18 (1.4-38)	40 (3.1-81)	10 (0.75-21)	22 (1.7-46)
JE	2000-2030	27 (1.9-55)	29 (2.2-60)	16 (1.1-33)	22 (1.5-44)
Measles	2000-2019	2100 (1500-2700)	450 (330-560)	2100 (1500-2600)	510 (380-640)
Measles	2020-2030	1500 (550-1900)	600 (210-720)	1500 (590-1800)	590 (230-710)
Measles	2000-2030	3600 (2300-4500)	500 (320-610)	3600 (2400-4400)	540 (360-660)
MenA	2000-2019	4.6 (0.76-9.6)	17 (2.8-37)	1.3 (0.17-2.7)	9.8 (1.3-20)
MenA	2020-2030	8.8 (3.3-17)	35 (13-67)	2.5 (0.82-4.4)	9.7 (3.2-18)
MenA	2000-2030	13 (9-22)	26 (17-43)	3.8 (2.3-5.8)	9.8 (6-15)
PCV	2000-2019	40 (20-68)	160 (84-280)	40 (20-68)	160 (84-280)
PCV	2020-2030	110 (47-200)	150 (67-290)	110 (47-200)	150 (67-290)
PCV	2000-2030	150 (67-270)	160 (71-290)	150 (67-270)	160 (71-290)
Rota	2000-2019	9.9 (6.5-14)	55 (36-74)	9.9 (6.5-14)	55 (36-74)
Rota	2020-2030	39 (23-56)	53 (32-75)	39 (23-56)	53 (32-75)
Rota	2000-2030	49 (30-69)	53 (33-75)	49 (30-69)	53 (33-75)
Rubella	2000-2019	8.7 (6.3-15)	6.1 (4.4-11)	8.7 (6.3-15)	7.1 (5.2-12)
Rubella	2020-2030	29 (19-49)	13 (8.6-22)	29 (19-49)	13 (8.8-22)

Rubella	2000-2030	38 (26-63)	10 (7-17)	38 (26-63)	11 (7.5-18)
YF	2000-2019	77 (26-160)	200 (68-420)	32 (9.8-66)	150 (46-310)
YF	2020-2030	130 (41-270)	500 (160-1100)	34 (10-73)	130 (41-290)
YF	2000-2030	210 (67-430)	320 (100-670)	66 (20-140)	140 (43-300)
Total	2000-2019	2400 (1800-3000)	240 (180-290)	2300 (1700-2900)	260 (200-330)
Total	2020-2030	2100 (1100-2700)	210 (110-260)	1900 (950-2300)	190 (99-240)
Total	2000-2030	4600 (3200-5500)	230 (150-270)	4200 (2900-5000)	230 (160-280)

Table S3(b): Estimated total deaths averted (in thousands) by vaccination and deaths averted per thousand individuals vaccinated in annual birth cohort ranges across the 98 countries considered, stratified by pathogen. Both lifetime and under-5 deaths averted are shown. The values are mean estimates and ranges are 95% credible intervals (2.5 and 97.5 quantiles). Estimates quoted to two significant figures. .

**Disease**	**Birth cohorts**	**Deaths averted (1000s)**	**Deaths averted per 1000 vaccinated individuals**	**Deaths averted (1000s), < 5**	**Deaths averted per 1000 vaccinated individuals, <5**
HepB	2000-2019	22000 (15000-32000)	13 (8.8-19)	300 (63-1100)	0.2 (0-0.6)
HepB	2020-2030	16000 (10000-25000)	9.8 (6.2-15)	230 (61-820)	0.1 (0-0.5)
HepB	2000-2030	38000 (25000-52000)	11 (7.5-16)	530 (130-2000)	0.2 (0-0.6)
Hib	2000-2019	1700 (760-2600)	2.8 (1.1-4.1)	1700 (760-2600)	2.8 (1.1-4.1)
Hib	2020-2030	2000 (730-3200)	2.4 (0.9-3.7)	2000 (730-3200)	2.4 (0.9-3.7)
Hib	2000-2030	3700 (1500-5800)	2.6 (1-3.9)	3700 (1500-5800)	2.6 (1-3.9)
HPV	2000-2019	3000 (2400-3500)	16 (12-18)	0 (0-0)	0 (0-0)
HPV	2020-2030	4000 (3500-4500)	12 (11-14)	0 (0-0)	0 (0-0)
HPV	2000-2030	7000 (6100-7900)	14 (11-16)	0 (0-0)	0 (0-0)
JE	2000-2019	180 (19-450)	0.4 (0-1)	64 (5.3-160)	0.2 (0-0.5)
JE	2020-2030	220 (23-560)	0.5 (0.1-1.2)	89 (8.3-230)	0.2 (0-0.5)
JE	2000-2030	400 (43-1100)	0.4 (0-1.1)	150 (13-390)	0.2 (0-0.5)
Measles	2000-2019	34000 (27000-45000)	7.2 (5.7-9.4)	34000 (26000-44000)	8.2 (6.5-11)
Measles	2020-2030	24000 (10000-31000)	9.3 (4-13)	23000 (10000-31000)	9.1 (4.2-12)
Measles	2000-2030	58000 (39000-76000)	8 (5.4-11)	57000 (40000-75000)	8.5 (6-12)
MenA	2000-2019	240 (130-390)	0.9 (0.5-1.5)	24 (7.5-43)	0.2 (0.1-0.3)

MenA	2020-2030	190 (83-320)	0.7 (0.3-1.3)	33 (14-57)	0.1 (0.1-0.2)
MenA	2000-2030	430 (220-690)	0.8 (0.4-1.3)	58 (36-87)	0.1 (0.1-0.2)
PCV	2000-2019	710 (360-1300)	2.9 (1.5-5)	710 (360-1300)	2.9 (1.5-5)
PCV	2020-2030	1600 (730-3000)	2.3 (1.1-4.3)	1600 (730-3000)	2.3 (1.1-4.3)
PCV	2000-2030	2400 (1000-4300)	2.5 (1.1-4.5)	2400 (1000-4300)	2.5 (1.1-4.5)
Rota	2000-2019	180 (110-250)	1 (0.6-1.4)	180 (110-250)	1 (0.6-1.4)
Rota	2020-2030	600 (370-840)	0.8 (0.5-1.1)	600 (370-840)	0.8 (0.5-1.1)
Rota	2000-2030	780 (480-1100)	0.8 (0.5-1.2)	780 (480-1100)	0.8 (0.5-1.2)
Rubella	2000-2019	80 (38-200)	0.1 (0-0.1)	80 (38-200)	0.1 (0-0.2)
Rubella	2020-2030	260 (130-590)	0.1 (0.1-0.3)	260 (130-590)	0.1 (0.1-0.3)
Rubella	2000-2030	340 (180-780)	0.1 (0-0.2)	340 (180-780)	0.1 (0.1-0.2)
YF	2000-2019	6000 (1900-12000)	16 (5-32)	590 (180-1300)	2.8 (0.9-5.7)
YF	2020-2030	4600 (1500-9400)	18 (5.9-37)	530 (160-1200)	2.1 (0.7-4.4)
YF	2000-2030	11000 (3400-22000)	17 (5.5-34)	1100 (340-2400)	2.4 (0.7-5)
Total	2000-2019	66000 (53000-80000)	6.4 (5.2-7.8)	37000 (29000-47000)	4.2 (3.4-5.4)
Total	2020-2030	52000 (37000-64000)	5.2 (3.7-6.3)	28000 (15000-36000)	2.9 (1.6-3.7)
Total	2000-2030	120000 (93000-150000)	5.8 (4.5-7)	65000 (48000-83000)	3.5 (2.7-4.5)

Table S3(c): Estimated total DALYs averted (in millions) by vaccination and deaths averted per thousand individuals vaccinated in annual birth cohort ranges across the 98 countries considered, stratified by pathogen. Both lifetime and under-5 DALYs averted are shown. The values are mean estimates and ranges are 95% credible intervals (2.5 and 97.5 quantiles). Estimates quoted to two significant figures.

**Disease**	**Birth cohorts**	**DALYs averted (millions)**	**DALYs averted per 1000 vaccinated individuals**	**DALYs averted (millions), < 5**	**DALYs averted per 1000 vaccinated individuals, <5**
HepB	2000-2019	870 (520-1400)	510 (300-800)	32 (5.4-110)	18 (3.2-62)
HepB	2020-2030	700 (390-1100)	430 (230-610)	25 (4.3-81)	15 (2.7-49)
HepB	2000-2030	1600 (910-2400)	470 (270-690)	57 (9.8-190)	17 (2.8-56)
Hib	2000-2019	110 (48-180)	180 (76-280)	110 (48-180)	180 (76-280)
Hib	2020-2030	140 (47-220)	160 (56-260)	140 (47-220)	160 (56-260)
Hib	2000-2030	250 (96-400)	170 (65-270)	250 (96-400)	170 (65-270)
HPV	2000-2019	67 (55-77)	350 (280-410)	0 (0-0)	0 (0-0)

HPV	2020-2030	110 (96-120)	330 (290-370)	0 (0-0)	0 (0-0)
HPV	2000-2030	170 (150-200)	340 (290-380)	0 (0-0)	0 (0-0)
JE	2000-2019	19 (1.5-39)	42 (3.4-85)	7.5 (0.49-16)	25 (1.7-53)
JE	2020-2030	23 (1.9-47)	49 (4.2-100)	10 (0.77-21)	23 (1.7-47)
JE	2000-2030	41 (3.4-84)	45 (3.7-92)	18 (1.2-37)	24 (1.7-49)
Measles	2000-2019	2200 (1600-2700)	470 (340-570)	2200 (1600-2700)	530 (390-650)
Measles	2020-2030	1600 (600-1900)	610 (230-740)	1500 (640-1800)	600 (250-710)
Measles	2000-2030	3800 (2300-4600)	520 (320-620)	3700 (2400-4500)	560 (360-670)
MenA	2000-2019	13 (8.4-20)	50 (32-75)	1.7 (0.54-3)	13 (4-22)
MenA	2020-2030	11 (5.8-18)	43 (23-71)	2.5 (1-4.2)	9.9 (4.2-17)
MenA	2000-2030	24 (14-37)	46 (28-71)	4.2 (2.7-6.3)	11 (7-17)
PCV	2000-2019	47 (22-82)	190 (94-340)	47 (22-82)	190 (94-340)
PCV	2020-2030	110 (48-210)	160 (68-300)	110 (48-210)	160 (68-300)
PCV	2000-2030	160 (70-290)	170 (75-310)	160 (70-290)	170 (75-310)
Rota	2000-2019	12 (7.1-17)	64 (39-93)	12 (7.1-17)	64 (39-93)
Rota	2020-2030	40 (24-57)	54 (33-77)	40 (24-57)	54 (33-77)
Rota	2000-2030	51 (32-74)	56 (34-80)	51 (32-74)	56 (34-80)
Rubella	2000-2019	8.7 (6.3-15)	6.1 (4.4-11)	8.7 (6.3-15)	7.1 (5.2-12)
Rubella	2020-2030	29 (19-49)	13 (8.6-22)	29 (19-49)	13 (8.8-22)
Rubella	2000-2030	38 (26-63)	10 (7-17)	38 (26-63)	11 (7.5-18)
YF	2000-2019	260 (83-530)	670 (210-1400)	38 (11-79)	180 (55-370)
YF	2020-2030	220 (70-450)	850 (270-1800)	36 (11-77)	140 (44-300)
YF	2000-2030	480 (150-970)	740 (240-1600)	74 (22-160)	160 (49-330)
Total	2000-2019	3500 (2600-4300)	340 (260-420)	2400 (1700-2900)	270 (200-330)
Total	2020-2030	2900 (1900-3500)	290 (190-350)	1900 (1000-2300)	200 (100-240)
Total	2000-2030	6400 (4600-7700)	320 (230-380)	4300 (2900-5100)	230 (160-280)

Table S4(a): Estimated total deaths averted (in thousands) by vaccination and deaths averted per thousand individuals vaccinated in different time period ranges across the 73 Gavi countries, stratified by pathogen. Both all age and under-5 deaths averted are shown. The values are mean estimates and ranges are 95% credible intervals (2.5 and 97.5 quantiles). Estimates quoted to two significant figures.

Disease	Time	Deaths averted (1000s)	Deaths averted per 1000 vaccinated individuals	Deaths averted (1000s), < 5	Deaths averted per 1000 vaccinated individuals, <5
HepB	2000-2019	380 (110-810)	0.2 (0.1-0.5)	160 (38-590)	0.1 (0-0.3)
HepB	2020-2030	1100 (290-2100)	0.6 (0.2-1.2)	160 (44-560)	0.1 (0-0.3)
HepB	2000-2030	1400 (410-2500)	0.4 (0.1-0.7)	320 (85-1200)	0.1 (0-0.3)
Hib	2000-2019	1500 (700-2300)	2.4 (1.1-3.5)	1500 (700-2300)	2.4 (1.1-3.5)
Hib	2020-2030	1900 (700-3100)	2.3 (0.8-3.6)	1900 (700-3100)	2.3 (0.8-3.6)
Hib	2000-2030	3500 (1400-5300)	2.4 (1-3.6)	3500 (1400-5300)	2.4 (1-3.6)
HPV	2000-2019	0.013 (0-0.031)	0 (0-0)	0 (0-0)	0 (0-0)
HPV	2020-2030	7.4 (1.4-15)	0 (0-0)	0 (0-0)	0 (0-0)
HPV	2000-2030	7.4 (1.4-15)	0 (0-0)	0 (0-0)	0 (0-0)
JE	2000-2019	26 (2.3-62)	0.1 (0-0.1)	11 (1.1-29)	0 (0-0.1)
JE	2020-2030	75 (7.9-200)	0.2 (0-0.4)	34 (4-110)	0.1 (0-0.2)
JE	2000-2030	100 (9.8-260)	0.1 (0-0.3)	45 (5-130)	0.1 (0-0.2)
Measles	2000-2019	31000 (25000-41000)	6.6 (5.2-8.6)	31000 (24000-41000)	7.5 (6-9.8)
Measles	2020-2030	22000 (9000-30000)	8.8 (3.6-12)	22000 (9700-29000)	8.6 (3.7-12)
Measles	2000-2030	54000 (37000-70000)	7.4 (5.2-9.6)	53000 (38000-69000)	8 (5.9-11)
MenA	2000-2019	73 (12-160)	0.3 (0-0.6)	19 (2.4-38)	0.1 (0-0.3)
MenA	2020-2030	140 (52-270)	0.5 (0.2-1.1)	33 (11-59)	0.1 (0-0.2)
MenA	2000-2030	210 (130-360)	0.4 (0.3-0.7)	52 (32-81)	0.1 (0.1-0.2)
PCV	2000-2019	600 (310-1000)	2.5 (1.3-4.1)	600 (310-1000)	2.5 (1.3-4.1)
PCV	2020-2030	1600 (710-2900)	2.2 (1-4)	1600 (710-2900)	2.2 (1-4)
PCV	2000-2030	2200 (1000-3900)	2.3 (1.1-4.1)	2200 (1000-3900)	2.3 (1.1-4.1)
Rota	2000-2019	150 (97-200)	0.8 (0.5-1.1)	150 (97-200)	0.8 (0.5-1.1)
Rota	2020-2030	580 (360-810)	0.8 (0.5-1.1)	580 (360-810)	0.8 (0.5-1.1)
Rota	2000-2030	730 (460-1000)	0.8 (0.5-1.1)	730 (460-1000)	0.8 (0.5-1.1)
Rubella	2000-2019	48 (25-130)	0 (0-0.1)	48 (25-130)	0 (0-0.1)
Rubella	2020-2030	220 (110-540)	0.1 (0.1-0.2)	220 (110-540)	0.1 (0.1-0.2)
Rubella	2000-2030	270 (140-660)	0.1 (0-0.2)	270 (140-660)	0.1 (0-0.2)
YF	2000-2019	1300 (450-2800)	3.5 (1.1-7.1)	500 (150-1100)	2.3 (0.7-4.9)

YF	2020-2030	2300 (740-4800)	9 (2.8-19)	510 (150-1100)	2 (0.6-4.2)
YF	2000-2030	3600 (1100-7500)	5.7 (1.8-12)	1000 (310-2200)	2.1 (0.7-4.5)
Total	2000-2019	35000 (28000-45000)	3.5 (2.7-4.4)	34000 (27000-44000)	3.9 (3.1-4.9)
Total	2020-2030	30000 (16000-38000)	3 (1.7-3.8)	27000 (14000-35000)	2.8 (1.5-3.5)
Total	2000-2030	66000 (49000-83000)	3.2 (2.5-4.1)	61000 (46000-77000)	3.3 (2.5-4.2)

Table S4(b): Estimated total DALYs averted (in millions) by vaccination and DALYs averted per thousand individuals vaccinated in different time period ranges across the 73 Gavi countries, stratified by pathogen. Both all age and under-5 DALYs averted estimates are shown. The values are mean estimates and ranges are 95% credible intervals (2.5 and 97.5 quantiles). Estimates quoted to two significant figures.

Disease	Time	DALYs averted (millions)	DALYs averted per 1000 vaccinated individuals	DALYs averted (millions), < 5	DALYs averted per 1000 vaccinated individuals, <5
HepB	2000-2019	35 (7.4-82)	20 (4.2-48)	16 (2.7-55)	9.6 (1.6-32)
HepB	2020-2030	82 (18-140)	50 (11-80)	17 (3-54)	10 (1.8-33)
HepB	2000-2030	120 (25-220)	34 (7.5-64)	33 (5.8-110)	9.9 (1.7-33)
Hib	2000-2019	100 (43-150)	160 (70-240)	100 (43-150)	160 (70-240)
Hib	2020-2030	130 (45-210)	160 (54-250)	130 (45-210)	160 (54-250)
Hib	2000-2030	230 (90-360)	160 (61-250)	230 (90-360)	160 (61-250)
HPV	2000-2019	0.0011 (0.00051-0.0021)	0 (0-0)	0 (0-0)	0 (0-0)
HPV	2020-2030	0.58 (0.27-0.93)	1.8 (0.8-2.8)	0 (0-0)	0 (0-0)
HPV	2000-2030	0.58 (0.27-0.93)	1.1 (0.5-1.8)	0 (0-0)	0 (0-0)
JE	2000-2019	2.7 (0.21-5.6)	6 (0.5-13)	1.2 (0.1-2.6)	4 (0.4-8.7)
JE	2020-2030	7.9 (0.69-17)	17 (1.5-37)	3.8 (0.37-9.4)	8.5 (0.8-21)
JE	2000-2030	11 (0.87-23)	12 (1-25)	5 (0.46-12)	6.7 (0.6-16)
Measles	2000-2019	2000 (1400-2500)	420 (310-520)	2000 (1400-2500)	480 (360-590)
Measles	2020-2030	1500 (520-1800)	570 (200-690)	1400 (560-1800)	560 (220-680)
Measles	2000-2030	3400 (2200-4200)	470 (300-580)	3400 (2200-4100)	510 (340-620)
MenA	2000-2019	4.6 (0.76-9.6)	17 (2.8-37)	1.3 (0.17-2.7)	9.8 (1.3-20)
MenA	2020-2030	8.8 (3.3-17)	35 (13-67)	2.5 (0.82-4.4)	9.7 (3.2-18)
MenA	2000-2030	13 (9-22)	26 (17-43)	3.8 (2.3-5.8)	9.8 (6-15)
PCV	2000-2019	39 (19-66)	160 (82-270)	39 (19-66)	160 (82-270)

PCV	2020-2030	110 (46-200)	150 (66-280)	110 (46-200)	150 (66-280)
PCV	2000-2030	150 (66-270)	150 (69-280)	150 (66-270)	150 (69-280)
Rota	2000-2019	9.5 (6.3-13)	53 (35-71)	9.5 (6.3-13)	53 (35-71)
Rota	2020-2030	39 (23-55)	52 (32-74)	39 (23-55)	52 (32-74)
Rota	2000-2030	48 (30-68)	52 (32-73)	48 (30-68)	52 (32-73)
Rubella	2000-2019	5.3 (3.9-9.2)	3.7 (2.7-6.4)	5.3 (3.9-9.2)	4.4 (3.2-7.4)
Rubella	2020-2030	24 (16-45)	11 (7.5-20)	24 (16-45)	11 (7.5-20)
Rubella	2000-2030	29 (21-54)	8 (5.7-15)	29 (21-54)	8.5 (6-16)
YF	2000-2019	77 (26-160)	200 (68-420)	32 (9.8-66)	150 (46-310)
YF	2020-2030	130 (41-270)	500 (160-1100)	34 (10-73)	130 (41-290)
YF	2000-2030	210 (67-430)	320 (100-670)	66 (20-140)	140 (43-300)
Total	2000-2019	2300 (1700-2800)	220 (170-270)	2200 (1600-2700)	250 (180-300)
Total	2020-2030	2000 (1000-2500)	200 (100-250)	1800 (900-2200)	180 (93-230)
Total	2000-2030	4200 (3000-5100)	210 (140-250)	3900 (2800-4700)	210 (150-260)

Table S4(c): Estimated total deaths averted (in thousands) by vaccination and deaths averted per thousand individuals vaccinated in different annual birth cohort ranges across the 73 Gavi countries, stratified by pathogen. Both lifetime and under-5 deaths averted are shown. The values are mean estimates and ranges are 95% credible intervals (2.5 and 97.5 quantiles). Estimates quoted to two significant figures.

**Disease**	**Birth cohorts**	**Deaths averted (1000s)**	**Deaths averted per 1000 vaccinated individuals**	**Deaths averted (1000s), < 5**	**Deaths averted per 1000 vaccinated individuals, <5**
HepB	2000-2019	13000 (8200-19000)	7.5 (4.7-11)	180 (48-630)	0.1 (0-0.4)
HepB	2020-2030	12000 (6800-21000)	7.2 (4.2-13)	170 (45-570)	0.1 (0-0.3)
HepB	2000-2030	25000 (15000-40000)	7.4 (4.5-12)	350 (95-1200)	0.1 (0-0.4)
Hib	2000-2019	1700 (730-2500)	2.7 (1.1-4)	1700 (730-2500)	2.7 (1.1-4)
Hib	2020-2030	1900 (700-3100)	2.3 (0.8-3.6)	1900 (700-3100)	2.3 (0.8-3.6)
Hib	2000-2030	3600 (1400-5600)	2.5 (1-3.8)	3600 (1400-5600)	2.5 (1-3.8)
HPV	2000-2019	3000 (2400-3500)	16 (12-18)	0 (0-0)	0 (0-0)
HPV	2020-2030	4000 (3500-4500)	12 (11-14)	0 (0-0)	0 (0-0)
HPV	2000-2030	7000 (6100-7900)	14 (11-16)	0 (0-0)	0 (0-0)

JE	2000-2019	89 (8.8-220)	0.2 (0-0.5)	14 (1.5-39)	0 (0-0.1)
JE	2020-2030	120 (13-310)	0.3 (0-0.7)	37 (4.4-120)	0.1 (0-0.3)
JE	2000-2030	210 (22-530)	0.2 (0-0.6)	52 (5.7-150)	0.1 (0-0.2)
Measles	2000-2019	33000 (25000-43000)	6.9 (5.4-8.9)	32000 (25000-42000)	7.8 (6-11)
Measles	2020-2030	23000 (9700-30000)	8.9 (3.7-12)	22000 (10000-29000)	8.8 (4-12)
Measles	2000-2030	56000 (37000-72000)	7.6 (5.2-9.8)	54000 (38000-71000)	8.2 (5.9-11)
MenA	2000-2019	240 (130-390)	0.9 (0.5-1.5)	24 (7.5-43)	0.2 (0.1-0.3)
MenA	2020-2030	190 (83-320)	0.7 (0.3-1.3)	33 (14-57)	0.1 (0.1-0.2)
MenA	2000-2030	430 (220-690)	0.8 (0.4-1.3)	58 (36-87)	0.1 (0.1-0.2)
PCV	2000-2019	700 (350-1200)	2.9 (1.5-4.9)	700 (350-1200)	2.9 (1.5-4.9)
PCV	2020-2030	1600 (720-3000)	2.3 (1-4.2)	1600 (720-3000)	2.3 (1-4.2)
PCV	2000-2030	2300 (1000-4200)	2.4 (1.1-4.4)	2300 (1000-4200)	2.4 (1.1-4.4)
Rota	2000-2019	170 (100-240)	0.9 (0.6-1.3)	170 (100-240)	0.9 (0.6-1.3)
Rota	2020-2030	590 (360-830)	0.8 (0.5-1.1)	590 (360-830)	0.8 (0.5-1.1)
Rota	2000-2030	760 (470-1100)	0.8 (0.5-1.2)	760 (470-1100)	0.8 (0.5-1.2)
Rubella	2000-2019	48 (25-130)	0 (0-0.1)	48 (25-130)	0 (0-0.1)
Rubella	2020-2030	220 (110-540)	0.1 (0.1-0.2)	220 (110-540)	0.1 (0.1-0.2)
Rubella	2000-2030	270 (140-660)	0.1 (0-0.2)	270 (140-660)	0.1 (0-0.2)
YF	2000-2019	6000 (1900-12000)	16 (5-32)	590 (180-1300)	2.8 (0.9-5.7)
YF	2020-2030	4600 (1500-9400)	18 (5.9-37)	530 (160-1200)	2.1 (0.7-4.4)
YF	2000-2030	11000 (3400-22000)	17 (5.5-34)	1100 (340-2400)	2.4 (0.7-5)
Total	2000-2019	55000 (44000-67000)	5.4 (4.2-6.5)	35000 (27000-45000)	4 (3.2-5)
Total	2020-2030	46000 (33000-59000)	4.6 (3.2-5.8)	27000 (15000-35000)	2.8 (1.6-3.5)
Total	2000-2030	100000 (78000-130000)	5 (3.9-6.1)	62000 (46000-78000)	3.4 (2.5-4.2)

Table S4(d): Estimated total DALYs averted (in millions) by vaccination and DALYs averted per thousand individuals vaccinated in different annual birth cohort ranges across the 73 Gavi countries, stratified by pathogen. Both lifetime and under-5 DALYs averted estimates are shown. The values are mean estimates and ranges are 95% credible intervals (2.5 and 97.5 quantiles). Estimates quoted to two significant figures.

**Disease**	**Birth cohorts**	**DALYs averted (millions)**	**DALYs averted per 1000 vaccinated individuals**	**DALYs averted (millions), < 5**	**DALYs averted per 1000 vaccinated individuals, <5**
HepB	2000-2019	510 (270-720)	290 (160-420)	32 (5.4-110)	18 (3.2-62)
HepB	2020-2030	510 (250-830)	310 (150-510)	25 (4.3-81)	15 (2.7-49)
HepB	2000-2030	1000 (530-1600)	300 (150-460)	57 (9.8-190)	17 (2.8-56)
Hib	2000-2019	110 (46-170)	180 (73-270)	110 (48-180)	180 (76-280)
Hib	2020-2030	130 (45-210)	160 (54-250)	140 (47-220)	160 (56-260)
Hib	2000-2030	240 (92-380)	160 (62-260)	250 (96-400)	170 (65-270)
HPV	2000-2019	67 (55-77)	350 (280-410)	0 (0-0)	0 (0-0)
HPV	2020-2030	110 (96-120)	330 (290-370)	0 (0-0)	0 (0-0)
HPV	2000-2030	170 (150-200)	340 (290-380)	0 (0-0)	0 (0-0)
JE	2000-2019	8.3 (0.64-17)	18 (1.3-37)	7.5 (0.49-16)	25 (1.7-53)
JE	2020-2030	12 (1-25)	26 (2.3-54)	10 (0.77-21)	23 (1.7-47)
JE	2000-2030	20 (1.7-41)	22 (1.8-44)	18 (1.2-37)	24 (1.7-49)
Measles	2000-2019	2100 (1500-2600)	440 (320-530)	2200 (1600-2700)	530 (390-650)
Measles	2020-2030	1500 (570-1800)	580 (220-710)	1500 (640-1800)	600 (250-710)
Measles	2000-2030	3600 (2200-4300)	490 (300-590)	3700 (2400-4500)	560 (360-670)
MenA	2000-2019	13 (8.4-20)	50 (32-75)	1.7 (0.54-3)	13 (4-22)
MenA	2020-2030	11 (5.8-18)	43 (23-71)	2.5 (1-4.2)	9.9 (4.2-17)
MenA	2000-2030	24 (14-37)	46 (28-71)	4.2 (2.7-6.3)	11 (7-17)
PCV	2000-2019	46 (22-80)	190 (91-330)	47 (22-82)	190 (94-340)
PCV	2020-2030	110 (47-210)	160 (66-290)	110 (48-210)	160 (68-300)
PCV	2000-2030	160 (69-290)	160 (73-300)	160 (70-290)	170 (75-310)
Rota	2000-2019	11 (6.8-16)	62 (38-90)	12 (7.1-17)	64 (39-93)
Rota	2020-2030	39 (24-57)	53 (32-76)	40 (24-57)	54 (33-77)
Rota	2000-2030	50 (31-73)	55 (34-79)	51 (32-74)	56 (34-80)
Rubella	2000-2019	5.3 (3.9-9.2)	3.7 (2.7-6.4)	8.7 (6.3-15)	7.1 (5.2-12)

Rubella	2020-2030	24 (16-45)	11 (7.5-20)	29 (19-49)	13 (8.8-22)
Rubella	2000-2030	29 (21-54)	8 (5.7-15)	38 (26-63)	11 (7.5-18)
YF	2000-2019	260 (83-530)	670 (210-1400)	38 (11-79)	180 (55-370)
YF	2020-2030	220 (70-450)	850 (270-1800)	36 (11-77)	140 (44-300)
YF	2000-2030	480 (150-970)	740 (240-1600)	74 (22-160)	160 (49-330)
Total	2000-2019	3000 (2200-3600)	290 (220-350)	2200 (1600-2700)	260 (190-310)
Total	2020-2030	2600 (1600-3200)	260 (160-320)	1800 (950-2200)	190 (98-230)
Total	2000-2030	5600 (4000-6700)	280 (190-330)	4000 (2800-4800)	220 (150-260)

Legends for Excel files

The following table legends describe the data in the Excel files with the corresponding file name as Table S-.xlsx. The two files are Table S5.xlsx and Table S6.xlsx each with sheets (a-l) showing estimates for different combinations of view (calendar year, year of birth), age (all ages, under 5s), and time period (2000-2019, 2020-2030, 2000-2030).

Table S5(a). Total deaths averted from all pathogens in each of the 98 countries among all age groups for 2000-2019 time periods, by calendar view (calendar year). The values are mean estimates and ranges are 95% credible intervals (2.5 and 97.5 quantiles).

Table S5(b). Total deaths averted from all pathogens in each of the 98 countries among all age groups for 2020-2030 time periods, by calendar view (calendar year). The values are mean estimates and ranges are 95% credible intervals (2.5 and 97.5 quantiles).

Table S5(c). Total deaths averted from all pathogens in each of the 98 countries among all age groups for 2000-2030 time periods, by calendar view (calendar year). The values are mean estimates and ranges are 95% credible intervals (2.5 and 97.5 quantiles).

Table S5(d). Total deaths averted from all pathogens in each of the 98 countries among all age groups for 2000-2019 time periods, by cohort view (year of birth). The values are mean estimates and ranges are 95% credible intervals (2.5 and 97.5 quantiles).

Table S5(e). Total deaths averted from all pathogens in each of the 98 countries among all age groups for 2020-2030 time periods, by cohort view (year of birth). The values are mean estimates and ranges are 95% credible intervals (2.5 and 97.5 quantiles).

Table S5(f). Total deaths averted from all pathogens in each of the 98 countries among all age groups for 2000-2030 time periods, by cohort view (year of birth). The values are mean estimates and ranges are 95% credible intervals (2.5 and 97.5 quantiles).

Table S5(g). Total deaths averted from all pathogens in each of the 98 countries among the under 5s age group for 2000-2019 time periods, by cohort view (year of birth). The values are mean estimates and ranges are 95% credible intervals (2.5 and 97.5 quantiles).

Table S5(h). Total deaths averted from all pathogens in each of the 98 countries among the under 5s age group for 2020-2030 time periods, by calendar view (calendar year). The values are mean estimates and ranges are 95% credible intervals (2.5 and 97.5 quantiles).

Table S5(i). Total deaths averted from all pathogens in each of the 98 countries among the under 5s age group for 2000-2030 time periods, by calendar view (calendar year). The values are mean estimates and ranges are 95% credible intervals (2.5 and 97.5 quantiles).

Table S5(j). Total deaths averted from all pathogens in each of the 98 countries among the under 5s age group for 2000-2019 time periods, by cohort view (year of birth). The values are mean estimates and ranges are 95% credible intervals (2.5 and 97.5 quantiles).

Table S5(k). Total deaths averted from all pathogens in each of the 98 countries among under 5s age group for 2020-2030 time periods, by cohort view (year of birth). The values are mean estimates and ranges are 95% credible intervals (2.5 and 97.5 quantiles).

Table S5(l). Total deaths averted from all pathogens in each of the 98 countries among under 5s age group for 2000-2030 time periods, by cohort view (year of birth). The values are mean estimates and ranges are 95% credible intervals (2.5 and 97.5 quantiles).

Table S6(a). Deaths averted per 1000 vaccinated individuals by pathogen for each country among all age groups for 2000-2019 time periods, by calendar view (calendar year). The values are mean estimates and ranges are 95% credible intervals (2.5 and 97.5 quantiles).

Table S6(b). Deaths averted per 1000 vaccinated individuals by pathogen for each country among all age groups for 2020-2030 time periods, by calendar view (calendar year). The values are mean estimates and ranges are 95% credible intervals (2.5 and 97.5 quantiles).

Table S6(c). Deaths averted per 1000 vaccinated individuals by pathogen for each country among all age groups for 2000-2030 time periods, by calendar view (calendar year). The values are mean estimates and ranges are 95% credible intervals (2.5 and 97.5 quantiles).

Table S6(d). Deaths averted per 1000 vaccinated individuals by pathogen for each country among all age groups for 2000-2019 time periods, by cohort view (year of birth). The values are mean estimates and ranges are 95% credible intervals (2.5 and 97.5 quantiles).

Table S6(e). Deaths averted per 1000 vaccinated individuals by pathogen for each country among all age groups for 2020-2030 time periods, by cohort view (year of birth). The values are mean estimates and ranges are 95% credible intervals (2.5 and 97.5 quantiles).

Table S6(f). Deaths averted per 1000 vaccinated individuals by pathogen for each country among all age groups for 2000-2030 time periods, by cohort view (year of birth). The values are mean estimates and ranges are 95% credible intervals (2.5 and 97.5 quantiles).

Table S6(g). Deaths averted per 1000 vaccinated individuals by pathogen for each country among the under 5s age group for 2000-2019 time periods, by calendar view (calendar year). The values are mean estimates and ranges are 95% credible intervals (2.5 and 97.5 quantiles).

Table S6(h). Deaths averted per 1000 vaccinated individuals by pathogen for each country among the under 5s age group for 2020-2030 time periods, by calendar view (calendar year). The values are mean estimates and ranges are 95% credible intervals (2.5 and 97.5 quantiles).

Table S6(i). Deaths averted per 1000 vaccinated individuals by pathogen for each country among the under 5s age group for 2000-2030 time periods, by calendar view (calendar year). The values are mean estimates and ranges are 95% credible intervals (2.5 and 97.5 quantiles).

Table S6(j). Deaths averted per 1000 vaccinated individuals by pathogen for each country among the under 5s age group for 2000-2019 time periods, by cohort view (year of birth). The values are mean estimates and ranges are 95% credible intervals (2.5 and 97.5 quantiles).

Table S6(k). Deaths averted per 1000 vaccinated individuals by pathogen for each country among the under 5s age group for 2020-2030 time periods, by cohort view (year of birth). The values are mean estimates and ranges are 95% credible intervals (2.5 and 97.5 quantiles).

Table S6(l). Deaths averted per 1000 vaccinated individuals by pathogen for each country among the under 5s age group for 2000-2030 time periods, by cohort view (year of birth). The values are mean estimates and ranges are 95% credible intervals (2.5 and 97.5 quantiles).

Table S7(a). Deaths averted per 1000 live births for each country among all age groups for 2019 birth cohorts. The values are mean estimates and ranges are 95% credible intervals (2.5 and 97.5 quantiles).

Table S7(b). Deaths averted per 1000 live births for each country among all age groups for 2000-2019 birth cohorts. The values are mean estimates and ranges are 95% credible intervals (2.5 and 97.5 quantiles).

Table S7(c). Deaths averted per 1000 live births for each country among all age groups for 2020-2030 birth cohorts. The values are mean estimates and ranges are 95% credible intervals (2.5 and 97.5 quantiles).

Table S7(d). Deaths averted per 1000 live births for each country among all age groups for 2000-2030 birth cohorts. The values are mean estimates and ranges are 95% credible intervals (2.5 and 97.5 quantiles).

Table S7(e). Deaths averted per 1000 live births for each country among under 5s age groups for 2019 birth cohorts. The values are mean estimates and ranges are 95% credible intervals (2.5 and 97.5 quantiles).

Table S7(f). Deaths averted per 1000 live births for each country among under 5s age groups for 2000-2019 birth cohorts. The values are mean estimates and ranges are 95% credible intervals (2.5 and 97.5 quantiles).

Table S7(g). Deaths averted per 1000 live births for each country among under 5s age groups for 2020-2030 birth cohorts. The values are mean estimates and ranges are 95% credible intervals (2.5 and 97.5 quantiles).

Table S7(h). Deaths averted per 1000 live births for each country among under 5s age groups for 2000-2030 birth cohorts. The values are mean estimates and ranges are 95% credible intervals (2.5 and 97.5 quantiles).

Figures

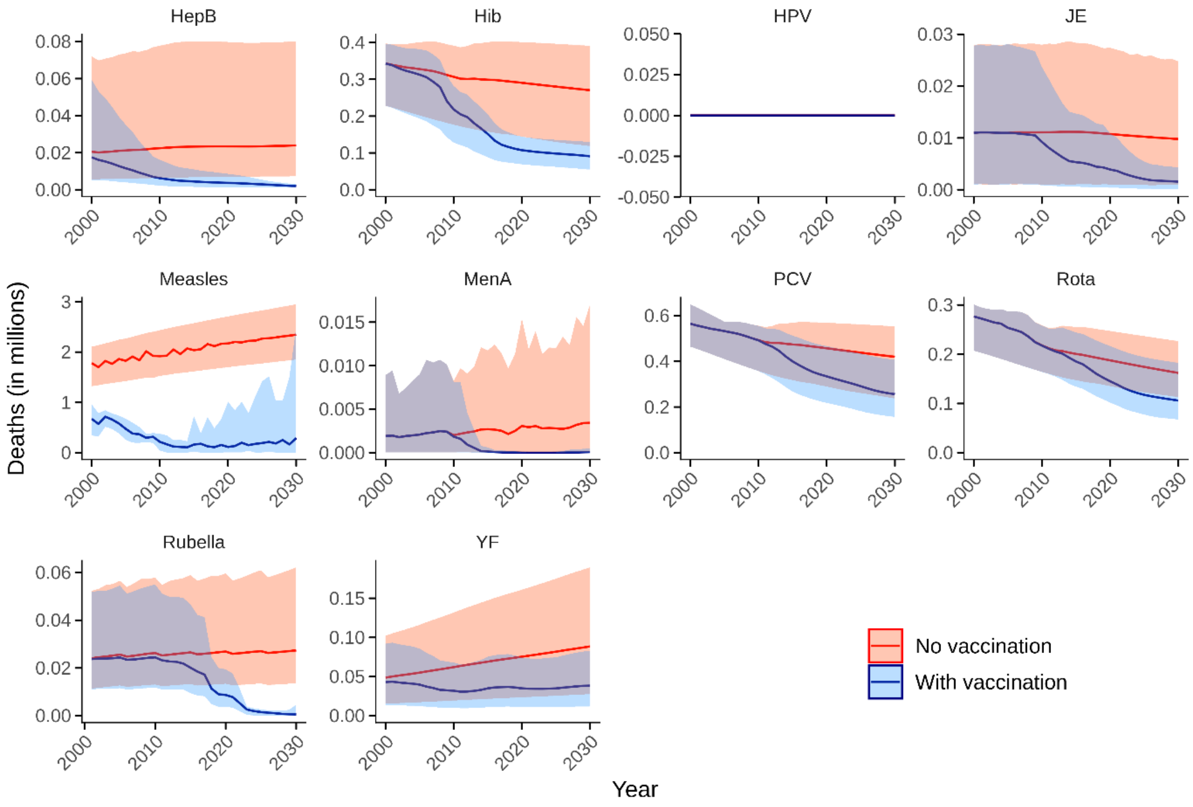

Figure S1 (a): Disease burden estimates in deaths by calendar year from 2000 to 2030 across all 98 countries for under-5s. Continuous red and blue lines show no vaccination and default vaccination coverage scenarios, respectively for the under 5s.

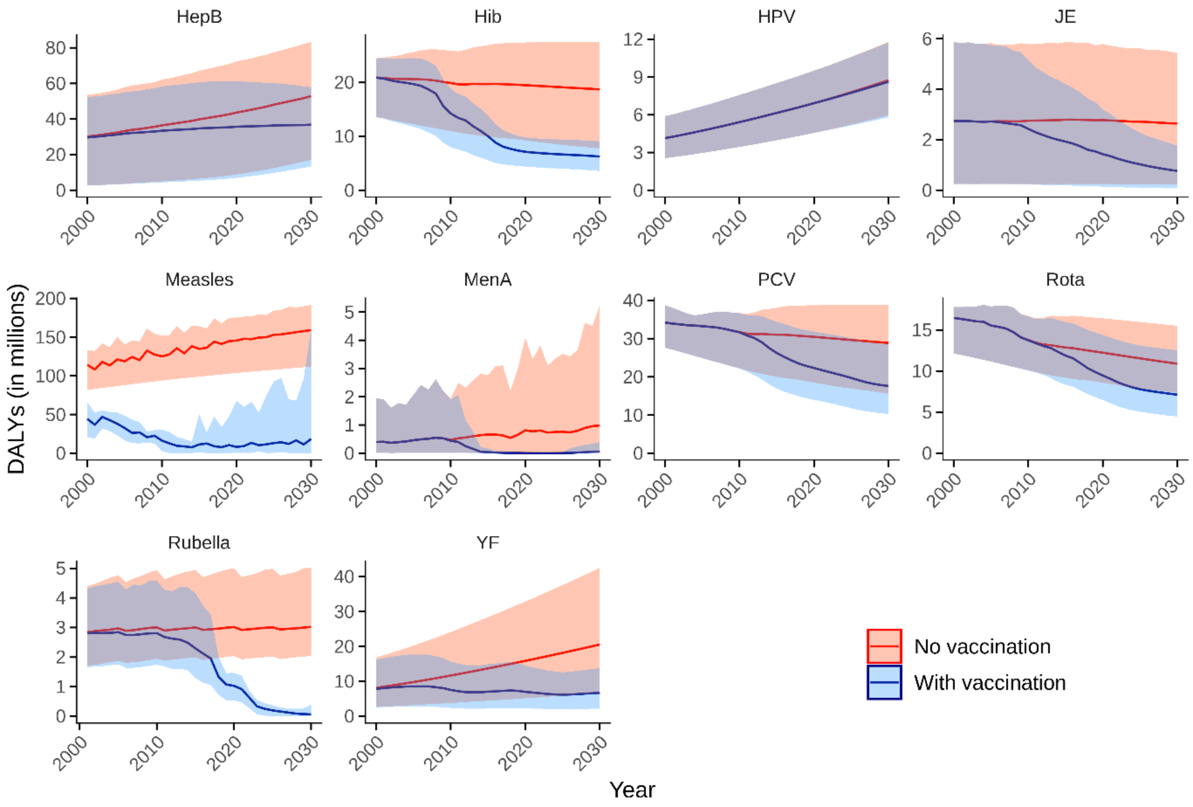

Figure S1(b): Disease burden estimates in DALYs by calendar year from 2000 to 2030 across all 98 countries for all ages. Continuous red and blue lines show no vaccination and default vaccination coverage scenarios, respectively, for all ages.

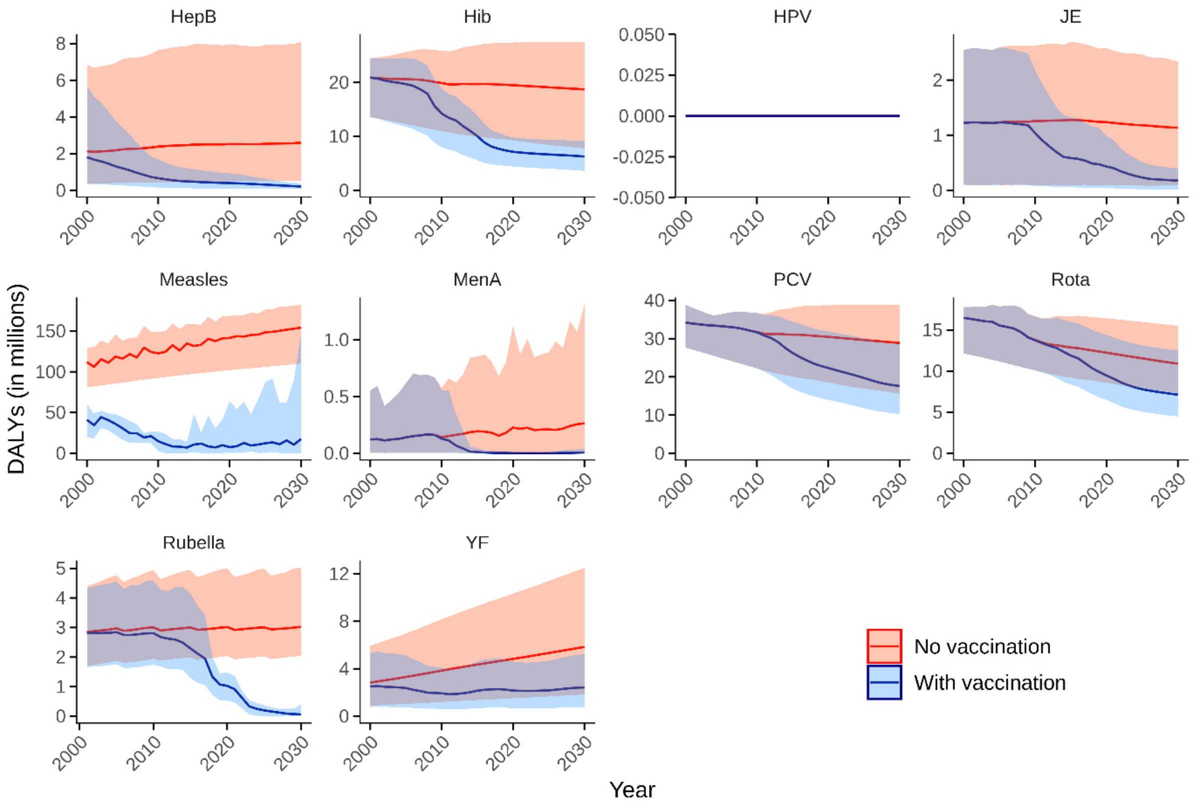

Figure S1(c): Disease burden estimates in DALYs by calendar year from 2000 to 2030 across all 98 countries for under-5s. Continuous red and blue lines show no vaccination and default vaccination coverage scenarios, respectively, for under 5s.

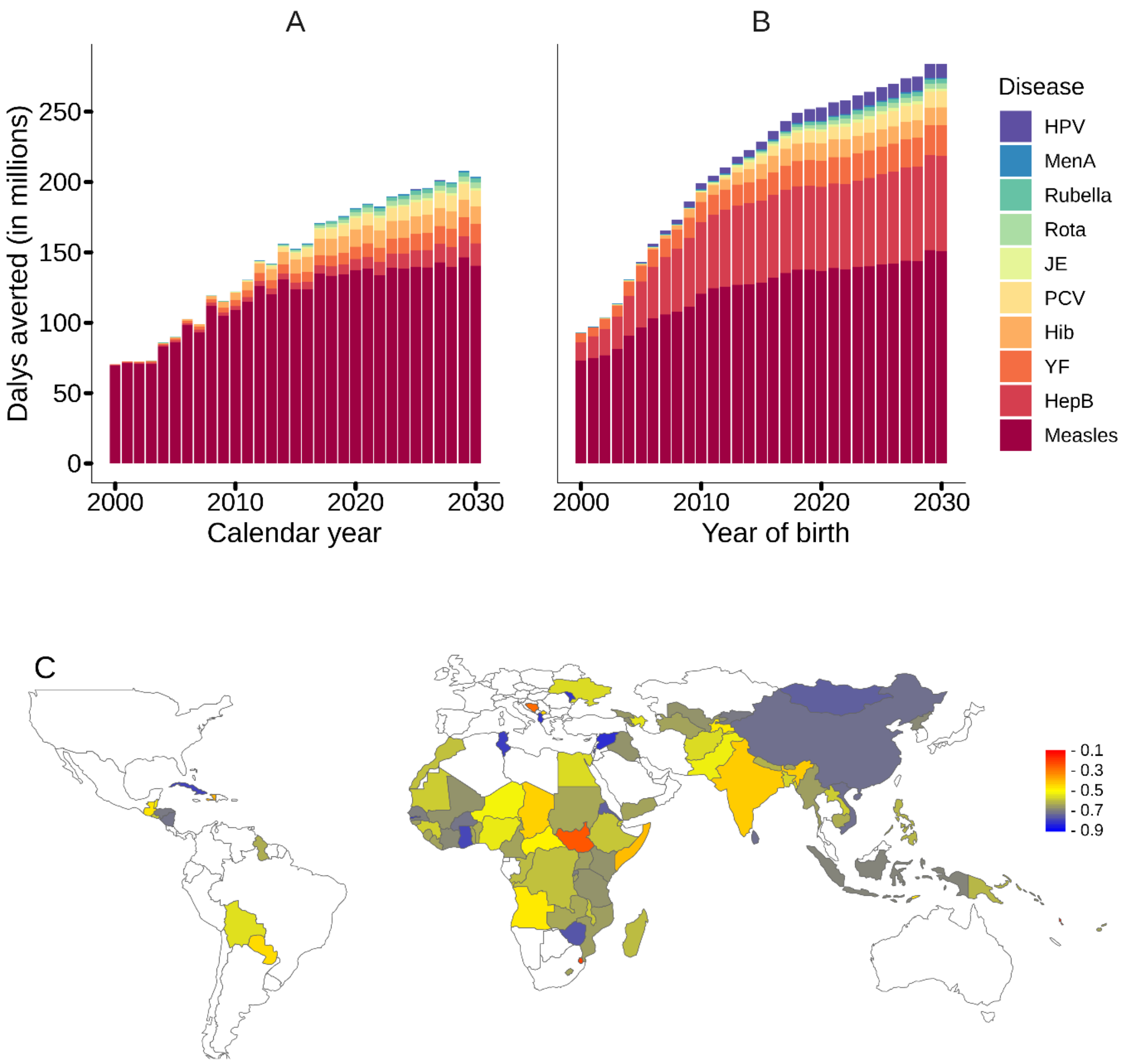

Figure S2: DALYs averted from 2000 to 2030 in the 98 countries: A) by calendar year (summing across all ages) and pathogen; B) by year of birth (summing across lifetime) and pathogen; C) proportion of lifetime DALYs due to the 10 pathogens in the no-vaccination counterfactual that are predicted to be averted by vaccination, by country across 2000-2018 birth cohorts.

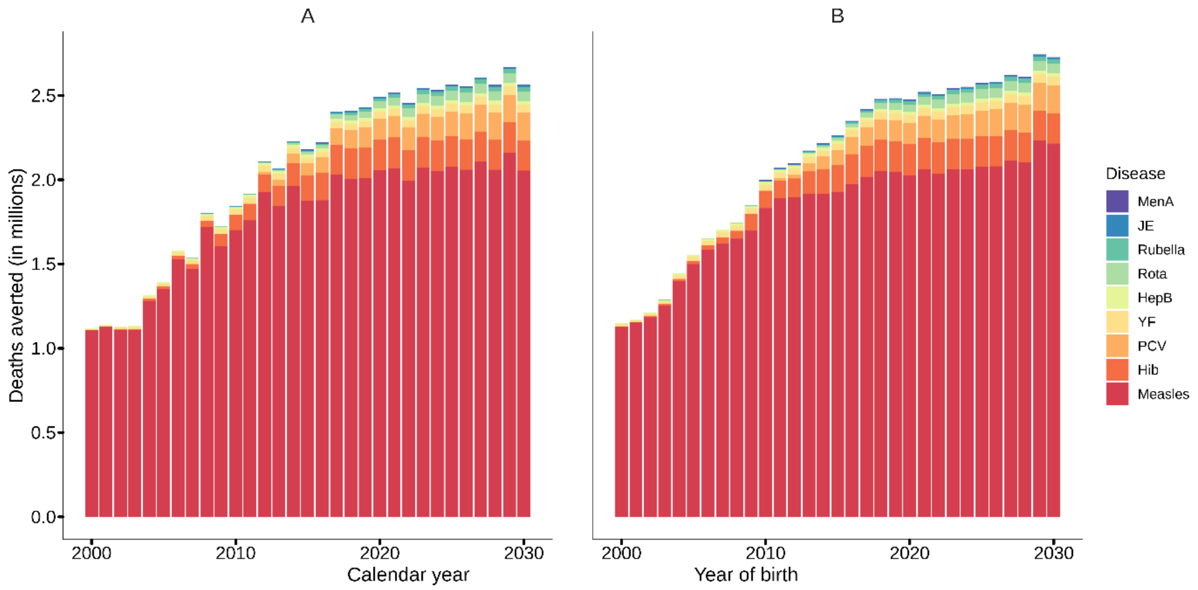

Figure S3 (a): Deaths averted from 2000 to 2030 in the 98 countries among the under 5s: A) by calendar year (summing across all ages) and pathogens; B) by year of birth (summing across lifetime) and pathogens.

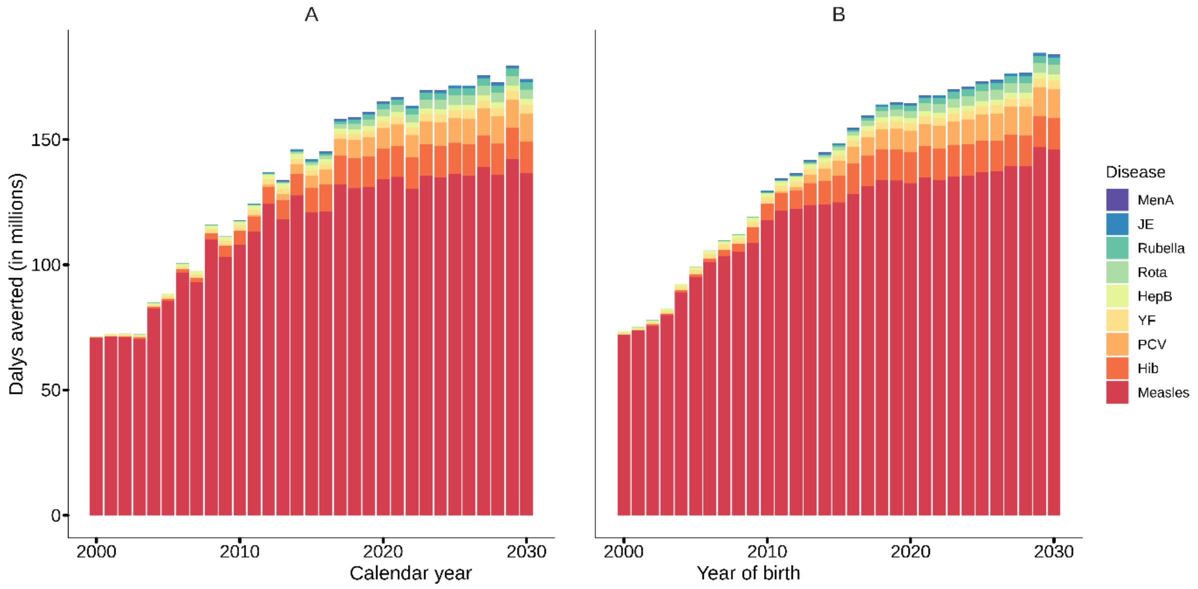

Figure S3 (b): DALYs averted from 2000 to 2030 in the 98 countries among the under 5s: A) by calendar year (summing across all ages) and pathogens; B) by year of birth (summing across lifetime) and pathogens.

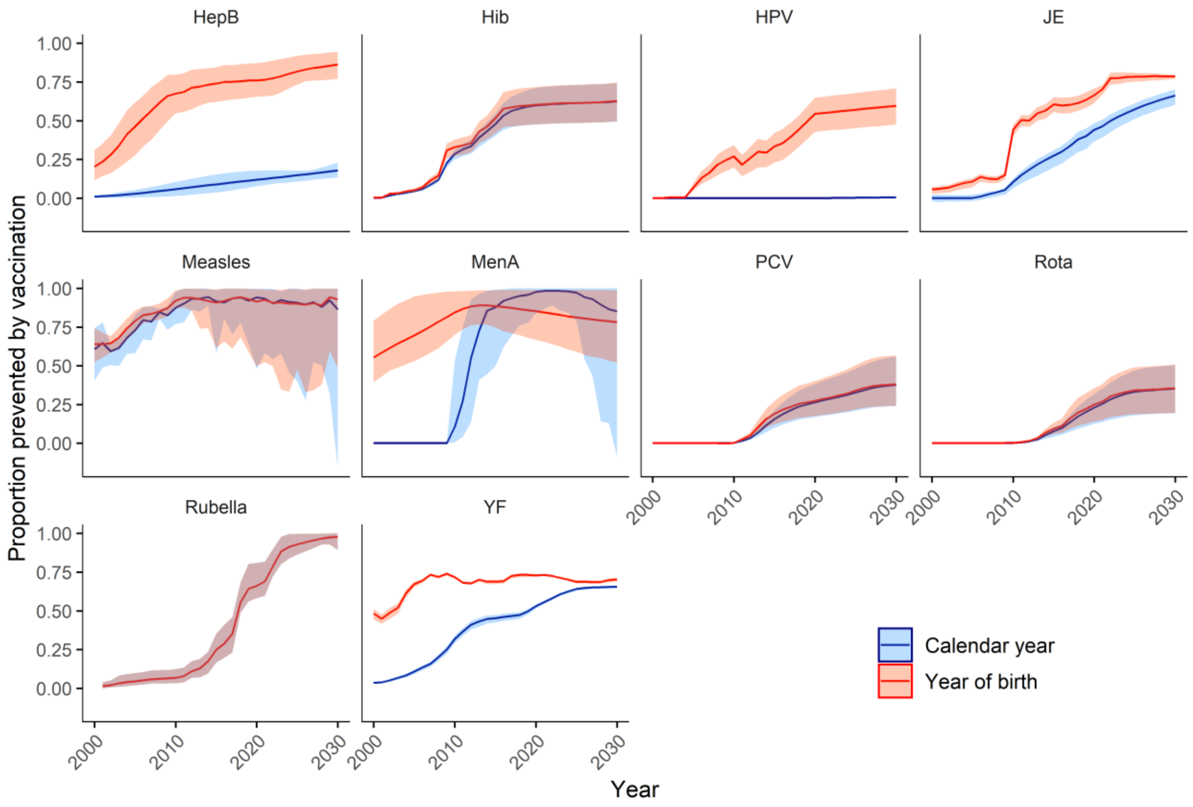

Figure S4 (a). Proportion of pathogen-specific deaths each year that would be prevented by vaccination by calendar year (blue) and year of birth (red). Solid lines show central estimates, shaded areas the 95% credible region.

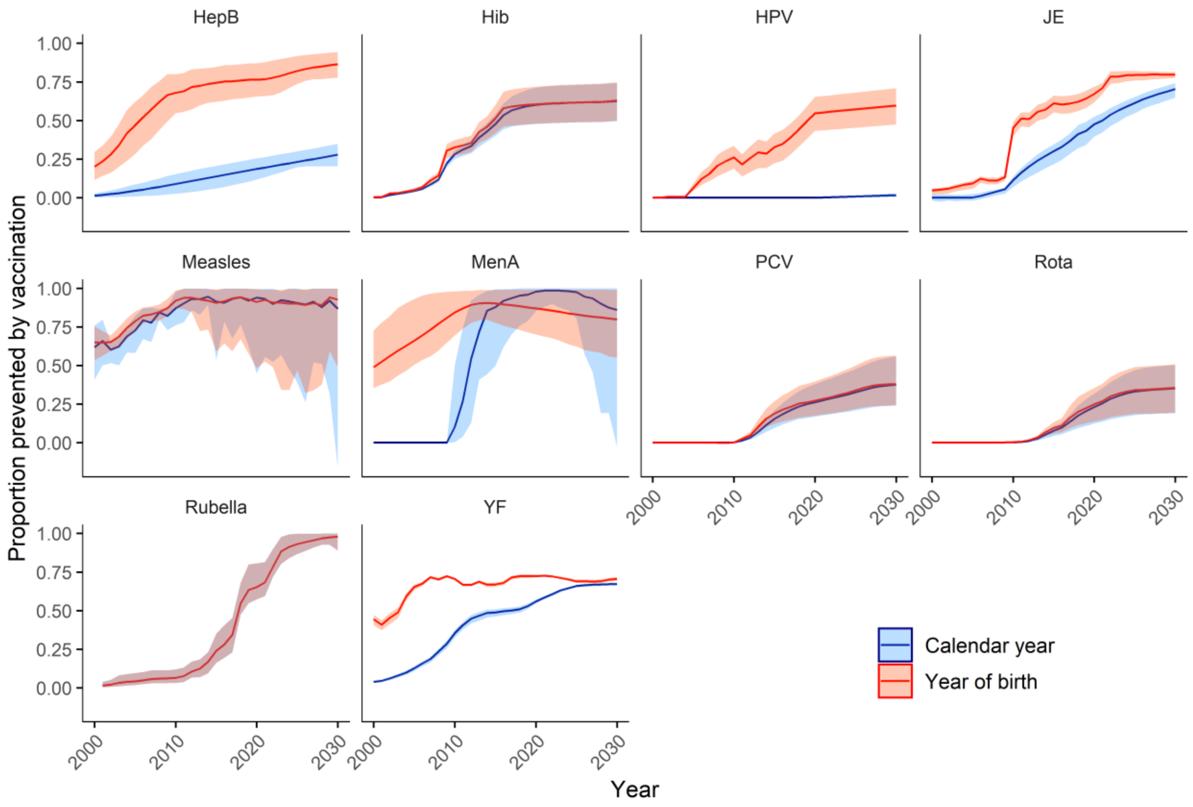

Figure S4 (b). Proportion of pathogen-specific DALYs each year that would be prevented by vaccination by calendar year (blue) and year of birth (red). Solid lines show central estimates, shaded areas the 95% credible region.
